# Human growth plates house resting zone sub-populations with features of quiescent stem cells

**DOI:** 10.1038/s41413-026-00564-y

**Published:** 2026-08-05

**Authors:** Mahtab Avijgan, Ana R. López-Pérez, Leire Alonso Galicia, Jose G. Marchan-Alvarez, Laura Sudupe, Ruihan Zhou, Amal Nazaraliyev, Žaneta Andrusivová, Ludvig Larsson, Pâmella Miranda, Doste R. Mamand, Yunhan Zhao, Farasat Zaman, Hong Qian, Klas Blomgren, Felipe Prosper, Oscar P. B. Wiklander, Jesper N. Tegner, Joakim Lundeberg, Lars Sävendahl, Reza Mirzazadeh, David Gomez-Cabrero, Phillip T. Newton

**Affiliations:** 1 Department of Women’s and Children’s Health, Karolinska Institutet, Stockholm, Sweden; 2 Astrid Lindgren Children’s hospital, Stockholm, Sweden; 3 Hematology and Oncology Program, Centre for Applied Medical Research (CIMA), Instituto de Investigaciones Sanitarias de Navarra (IdiSNA), Cancer Center Clínica Universidad de Navarra (CCUN), Pamplona, Navarra Spain; 4 Department of Gene Technology, KTH Royal Institute of Technology, Science for Life Laboratory, Stockholm, Sweden; 5 Division of Biomedical Sciences, King Abdullah University of Science and Technology KAUST, Thuwal, Saudi Arabia; 6 Department of Laboratory Medicine, Unit for Biomolecular and Cellular Medicine, Karolinska Institutet, Stockholm, Sweden; 7 Breast Center, Karolinska Comprehensive Cancer Center, Karolinska University Hospital, Stockholm, Sweden; 8 Center for Hematology and Regenerative Medicine (HERM), Department of Medicine Huddinge, Karolinska Institute, Stockholm, Sweden; 9 Paediatric Oncology, Karolinska University Hospital, Stockholm, Sweden; 10 Hematology-Oncology and Regenerative Medicine, Clínica Universidad de Navarra and Center for Applied Medical Research, University of Navarra, Pamplona, Spain; 11 Instituto de Investigación Sanitaria de Navarra (IdiSNA), Pamplona, Spain; 12 Centro de Investigación Biomédica en Red de Cáncer, CIBERONC, Instituto de Salud Carlos III, Madrid, Spain; 13 Computer, Electrical and Mathematical Sciences and Engineering Division, King Abdullah University of Science and Technology (KAUST), Thuwal, Saudi Arabia; 14 Unit of Computational Medicine, Department of Medicine, Center for Molecular Medicine, Karolinska Institutet, Karolinska University Hospital, Stockholm, Sweden; 15 Science for Life Laboratory, Solna, Sweden

**Keywords:** Physiology, Bone

## Abstract

Maintaining postnatal bone growth is crucial for humans to reach their final height. To determine transcriptional networks coordinating this process, we applied spatially resolved transcriptomics to growth plate biopsies obtained from healthy adolescents who underwent epiphysiodesis surgery for idiopathic tall stature. Spatial profiling revealed new markers for each zone of the human growth plate and identified genes associated with poorly understood growth disorders, including the novel hypertrophic zone marker SGMS2. We elaborated on this finding and established that Sgms2 is present in growth plate-derived matrix vesicles, and its activity facilitates mineralization - a process impaired in patients with SGMS2 mutations. By exploring the low transcriptional activity of resting zone chondrocytes, we found that a subset of these cells exists in a functionally quiescent state in vivo, as determined by their predominantly nuclear mRNA, abundant heterochromatin, and ability to exit the G0 phase under specific conditions - features shared with skeletal stem cells in mouse growth plates. Additionally, we identified distinct sub-populations of human resting zone chondrocytes; an exploration of their hierarchy determined that CHRDL2 and/or SFRP5-positive sub-populations were among the least quiescent resting zone cells. In summary, we generated a comprehensive map of gene expression within the human growth plate, revealing novel zone-specific markers, new primary growth disorders, candidate pharmacological targets, and sub-populations of resting zone chondrocytes with features of quiescent stem cells. These results contribute to a better understanding of the cellular and molecular mechanisms governing human height and can facilitate improved diagnosis and treatment strategies for patients with skeletal growth disorders.

## Introduction

During childhood and adolescence, humans grow taller because tubular bones, such as femora and tibiae, increase in length. These bones are elongated by narrow cartilage discs called (epiphyseal) growth plates, which are positioned between the bony primary and secondary ossification centers (POC and SOC, respectively).^1^ Histologically, each growth plate can be divided into three distinct zones: directly adjacent to the SOC, slowly dividing chondro-progenitors comprise the resting zone (RZ); some of these cells give rise to columns of flattened chondrocytes that undergo several cycles of cell division in the proliferating zone (PZ), and subsequently differentiate to form the hypertrophic zone (HZ), producing a mineralized matrix that is used as a scaffold for new bone tissue to be built on.^2^ Since bone elongation continues until the end of puberty, its regulation is crucial for humans to reach their expected height and body proportions.

Skeletal growth disorders are categorized as primary (directly affecting growth plates), secondary (indirectly affecting growth plates, via, for example, hormones) or idiopathic.^3^ However, distinguishing primary growth disorders from secondary and elucidating idiopathic ones remains challenging.^4^ Many growth plate markers are known only from experiments with model organisms, and their relevance to humans remains uncertain. This knowledge gap is also reflected by the unrefined nature of therapeutic approaches: the most common treatment for patients with idiopathic short stature with growth hormone (GH) sufficiency is recombinant GH, particularly in the US.^4^ For idiopathic tall stature adolescents, only invasive surgery to remove the growth plate has been proven to be a safe and effective treatment.^5^ Incomplete mapping of gene expression and the cell populations present within human growth plates contributes to a poor understanding of the mechanisms underpinning human skeletal growth and a lack of pharmacological targets to treat growth disorders with precision.

To better describe the human growth plate, we applied spatially resolved transcriptomics (SRT) with 10x Genomics’ Visium platform to sections obtained from healthy adolescents with idiopathic tall stature. This approach allowed us to obtain gene expression profiles while maintaining cellular positioning and growth plate architecture. We identified a panel of 138 genes specifically expressed in individual growth plate zones and SOC, allowing us to verify growth plate markers reported in other species, identify novel markers of each growth plate zone and uncover previously unknown primary growth disorders; an elaboration on novel HZ marker *SGMS2* revealed mechanistic insights into its role in mineralization - a process impaired in patients with *SGMS2* mutations. Furthermore, by elaborating upon their low transcriptional activity, we found that RZ chondrocytes reside in a functionally quiescent state in vivo and have similar properties to skeletal stem cells in murine growth plates. Finally, by exploring the expression of RZ markers in association with quiescence, we revealed a potential hierarchy among sub-populations of RZ cells.

## Results

### Resting zone chondrocytes contain relatively low amounts of mRNA

**Fig. 1:**
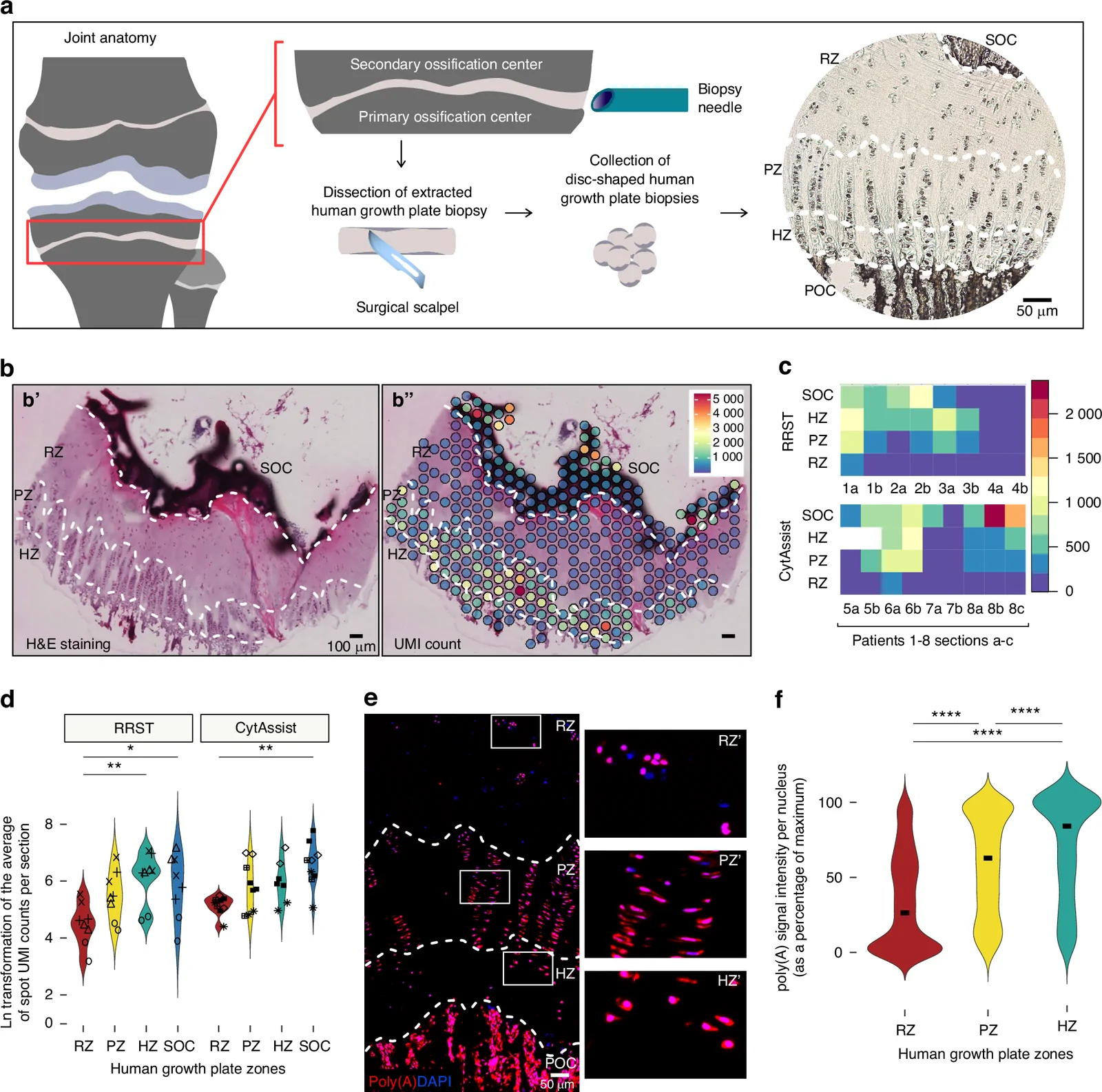
Resting zone chondrocytes contain relatively low amounts of mRNA. **a** The procedure for collecting human growth plate biopsies. **b** Hematoxylin and eosin (H&E)-stained tissue section (b´) and spot UMI counts from RRST profiling (b´´). **c** Level-plot of UMI counts per section. **d** Violin plot of spot UMI counts in each zone. **e** Representative image of RNAscope assay targeting poly(A) expression. **f** Mean signal intensity per nucleus in each zone converted to a percentage value using the maximum value for each patient

We obtained biopsies containing human growth plates from healthy adolescents aged 12 to 14 years who underwent epiphysiodesis surgery for idiopathic tall stature (Fig. 1a, Table 1). Biopsies spanning the growth plate and SOC were prepared for analysis by 10x Genomics’ Visium as per our established RNA-Rescue Spatial Transcriptomics (RRST) protocol, to examine spatial gene expression profiles within non-decalcified cartilage.^6^ First, we applied RRST to human growth plate biopsies (Fig. 1b), using histology to manually define four epiphyseal areas: SOC, RZ, PZ and HZ. Spots corresponding to cartilage had relatively low unique molecular identifier (UMI) counts (total number of transcripts), compared to areas containing bone marrow (Figs. 1b, c, S1a), which were consistent with other tissues.^6^

**Table 1:** SRT Exploration dataset

Patient ID	Platform	Genetic sex	Tanner pubertal stage PH^a^	Tanner pubertal stage B^b^	Tanner pubertal stage G^c^	Patient age at surgery
1	RRST	M	3	-	3	13 years 2 months
2	RRST	F	3	4	-	12 years 10 months
3	RRST	M	3	-	3	13 years 4 months
4	RRST	F	3 to 4	4	-	12 years 11 months
5	CytAssist	M	2	-	2	12 years 2 months
6	CytAssist	F	4	4	-	14 years 6 months
7	CytAssist	M	4	-	4	13 years 7 months
8	CytAssist	F	4	4	-	14 years 4 months

Metadata of the patient information for the tissues analyzed in Figs. 1c, d, 3a-c and Table 2

^a^Pubic hair.

^b^Female breast.

^c^Male genital.

To validate these findings, we utilized the Visium CytAssist Spatial Gene Expression protocol, where an average of three pairs of probes target each transcript (compared to only one pair using RRST), which provided comparable results to RRST experiments. Although the average number of cells captured in each Visium spot was similar throughout the growth plate (Fig. S1b), relatively few transcripts were captured in the RZ (Fig. 1b-d, Table 2). To understand the biological relevance of this transcription pattern, we used in situ hybridization to visualize polyadenylated (poly[A]) mRNA molecules within the human growth plate sections (Fig. 1e). This experiment confirmed that RZ cells contained less mRNA than other growth plate chondrocytes (Fig. 1f).

**Table 2:** Average number of UMI counts per spot from SRT

Platform	RRST								
PatientID_section	1_1	1_2	2_1	2_2	3_1	3_2	4_1	4_2	
SOC	879.51	506.78	897.15	1 405.67	324.05	213.84	57.53	111.12	
RZ	237.90	179.59	81.47	71.07	101.13	106.97	23.81	47.35	
PZ	893.27	369.98	157.56	190.88	551.75	237.83	68.01	83.96	
HZ	1 078.94	505.75	512.91	525.73	1 073.84	538.24	134.29	109.61	
POC	432.17	222	NA	NA	1 963.05	1 736.97	352.81	279.25	
Platform	CytAssist								
PatientID_section	5_1	5_2	6_1	6_2	7_1	7_2	8_1	8_2	8_3
SOC	453.61	1 007.11	790.70	1 061.44	629.73	90.81	1 007.11	2 652.26	1 863.61
RZ	194.33	170.33	259.01	158.69	71.97	82.98	217.63	205.93	139.76
PZ	118.80	504.45	1 037.14	417.06	115.76	126.02	298.45	257.08	237.64
HZ	NA	NA	936.38	1 429.23	188.69	148.57	299.28	422.53	633.30
POC	NA	NA	1 281.35	NA	428.18	285.77	1 639.79	2 252.82	3 721.41

The average of UMI counts per spot was calculated from SRT data from each area of every section. Where sections lacked specific areas, they are denoted NA

Consistent with our findings in human samples, mouse RZ cells contained low levels of mRNA molecules in comparison to their neighbors (Fig. S2a-c).

### Resting zone chondrocytes display hallmarks of quiescent stem cells

**Fig. 2:**
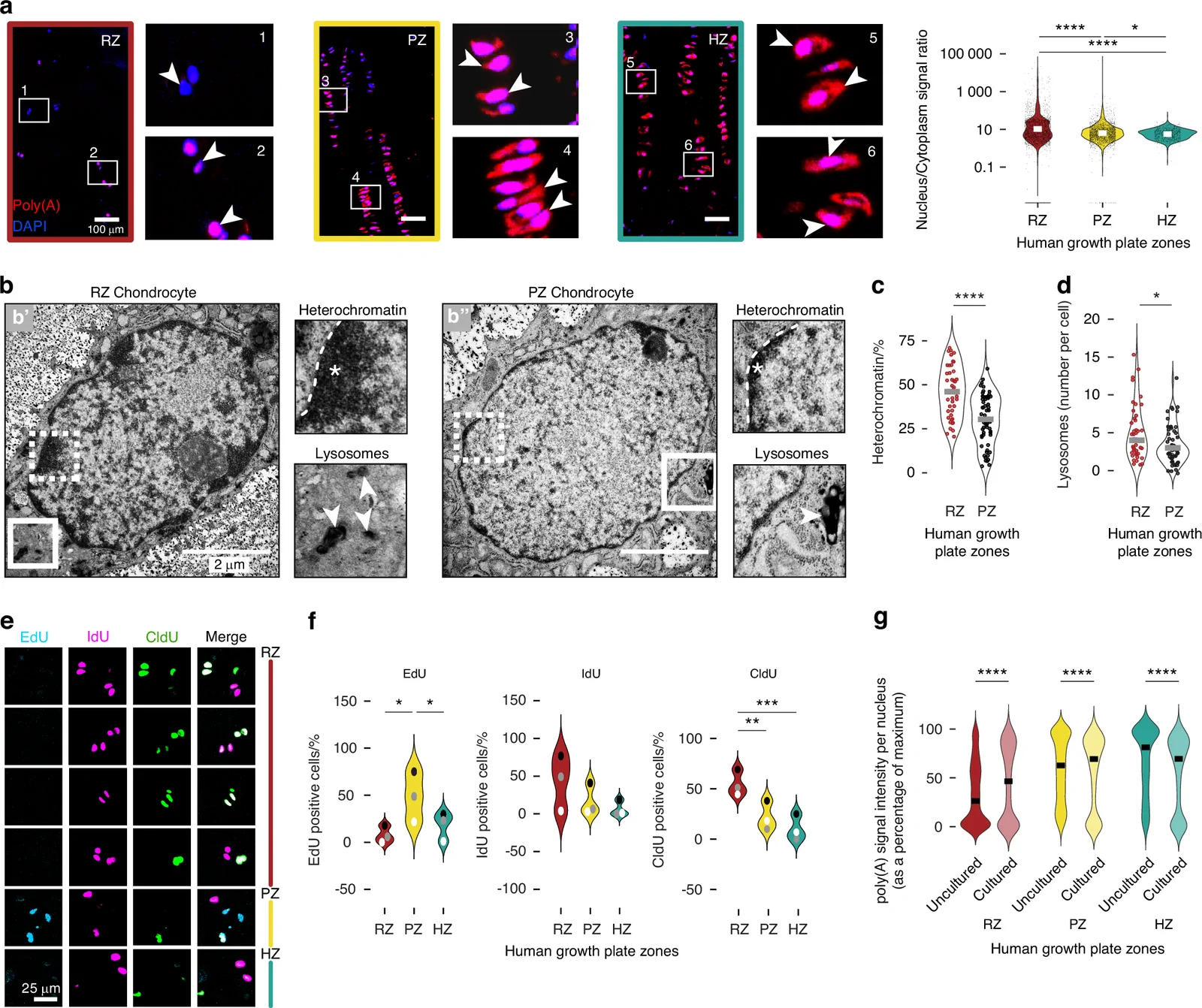
Resting zone chondrocytes display features of cellular quiescence in vivo. **a** Subcellular localization of poly(A) mRNA. Note the predominant nuclear localization in RZ (arrowheads). **b** TEM images of heterochromatin (asterisks) and lysosomes (arrowheads) in RZ and PZ chondrocytes. **c** The percentage of each nucleus comprising heterochromatin, and **d** the number of lysosomes per cell. **e** High magnification images (from Fig. S3d,e) indicating zone-specific thymidine-analogue labelling of chondrocytes, and **f** corresponding quantification of EdU, IdU and CldU incorporation. **g** Post-culture poly(A) RNA levels were compared with fresh-frozen slices (presented in Fig. 1f)

Having determined via independent methods, and in two species, that RZ cells contain less mRNA than other growth plate chondrocytes, we investigated the biological relevance of this finding. While quantifying polyadenylated mRNA, we noticed that it was predominantly localized to nuclei of RZ cells, whereas in other chondrocytes, mRNA was distributed between the nucleus and cytoplasm (Figs. 2a, S2b). Indeed, the relative amount of mRNA found in the nucleus was lower in the PZ and HZ chondrocytes than in the RZ (Figs. 2a, S2d). Predominantly nuclear localization of mRNA is a feature of cellular quiescence across multiple species and tissues, including deeply quiescent neural stem cells and hematopoietic stem and progenitor cells.^7^ Cellular quiescence (or more simply, quiescence) is a state of reversible cell cycle arrest, which is a characteristic of long-living adult stem cells, priming them in a “poised” state that enables them to act in tissue homeostasis or regeneration.^8,9^ While RZ cells are often referred to as quiescent, this description is based on their relatively low cell division rates in rodents.^10^

To elaborate upon cellular quiescence in the RZ, we conducted transmission electron microscopy (TEM) to compare the ultrastructure of RZ cells with neighboring PZ cells (Fig. 2b). Notably, RZ chondrocytes had a higher content of normally condensed, electron-dense, heterochromatin (Fig. 2c), typically located at the inner leaflet of the nuclear membrane (Fig. 2b’). In contrast, PZ chondrocytes exhibited discrete areas of heterochromatin within their nuclei (Fig. 2b”). Consistent with quiescent cells in multiple other tissues^11,12^, RZ chondrocytes contained more lysosomes than PZ chondrocytes (Fig. 2d). Altogether, these results indicate that, as a population, human RZ cells display features of cellular quiescence in vivo.

The murine RZ contains epiphyseal growth plate stem cells.^13,14^ A subset of slowly-dividing, label-retaining cells (LRCs) in the RZ have been identified by their retention of tritiated thymidine,^10,15^ BrdU analogues,^13,16^ or H2B-GFP^17,18^ in pulse-chase experiments. While it remains to be determined whether all LRCs are stem cells, progeny of some LRCs can give rise to chondrocyte columns, suggesting that some epiphyseal stem cells are among the LRCs.^17,18^ By combining RNAscope with EdU detection (Fig. S3a-c), we determined that LRCs had significantly lower mRNA levels than non-LRCs, suggesting that low mRNA levels are a strong indicator of LRCs. Despite this, some LRCs had elevated mRNA levels, either reflecting that not all LRCs are quiescent or that they were in the process of leaving quiescence at tissue collection.

### Human resting zone chondrocytes are functionally quiescent in vivo

Relatively low RNA content is an indicator of the G0 phase of the cell cycle in multiple cell types.^19^ To explore the cell dynamics of human growth plate chondrocytes, intact biopsies were cultured ex vivo over a time-course with a sequence of distinct thymidine analogues (Fig. S3d); freshly collected biopsies were cultured in EdU-containing medium overnight to capture ongoing proliferation, then EdU was replaced by IdU and then CldU over subsequent days. As anticipated, PZ cells were labelled with EdU using this strategy, whereas few RZ cells incorporated EdU (Figs. 2e, f, S3e). However, IdU and CldU were present in RZ cells (Fisg. 2e, f, S3e), suggesting that some RZ cells are capable of reversible cell cycle arrest. These findings align with quiescent muscle satellite cells,^20^ hematopoietic stem cells,^21^ and neural stem cells,^22^ which begin dividing when isolated from their milieu and cultured ex vivo.

Cultured human biopsies contained a proportion of RZ cells with more mRNA than uncultured biopsies (Figs. S3f, 2g), supporting the notion that fewer RZ cells remained quiescent after culture. Although we cannot exclude a prolonged G1 phase in our thymidine-analogue labelling experiments, our combined observations of mRNA localization and cellular ultrastructure lead us to propose that a subset of human RZ cells exists in a functionally quiescent state in vivo as they retain the capacity to become functionally active and re-enter the cell cycle.

### Spatially resolved transcriptomics reveals novel human growth plate markers

**Fig. 3:**
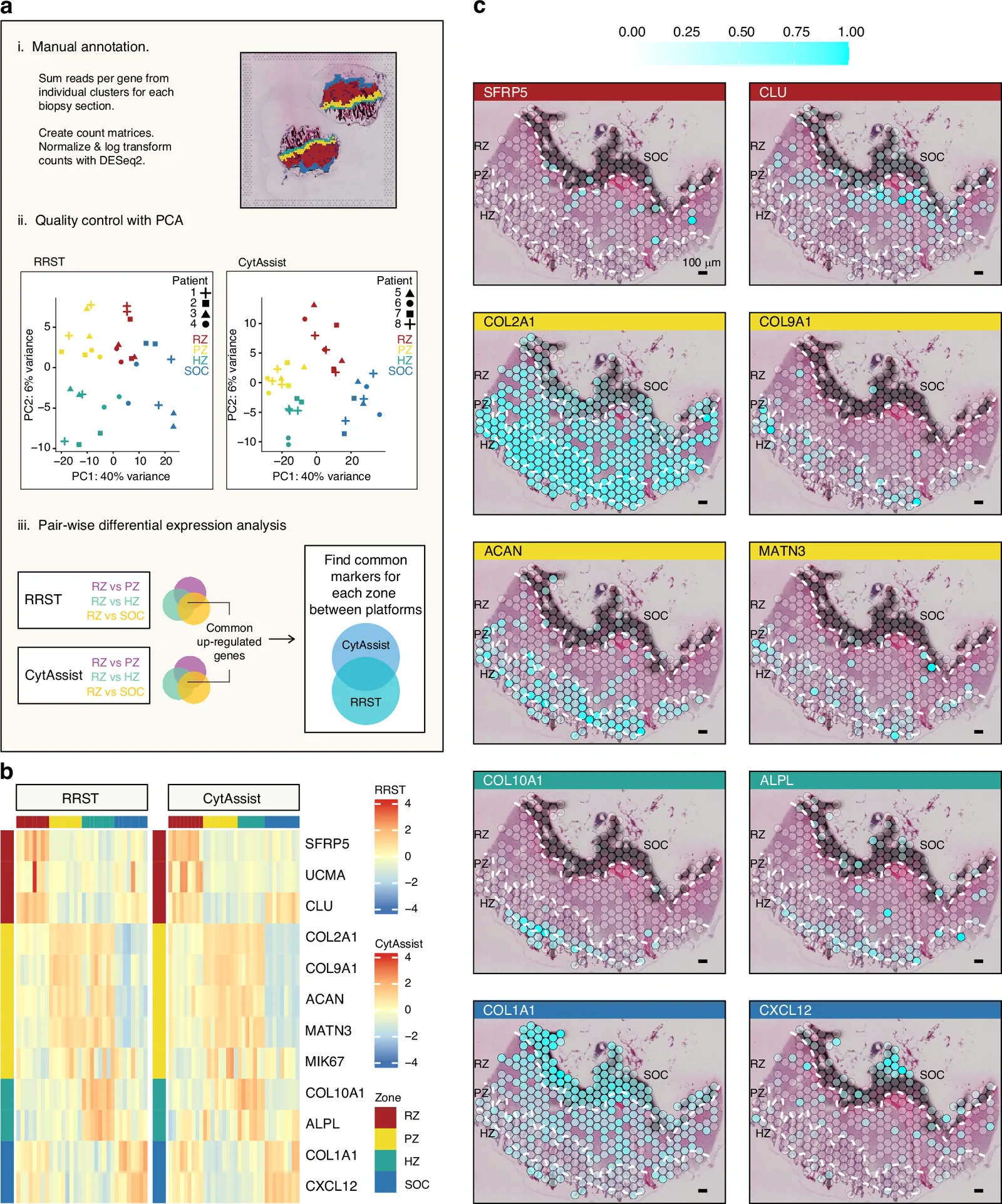
Spatial profiles of cartilage and SOC markers visualized on human growth plate sections. **a** Pseudo-bulk analysis workflow used to analyze SRT data. SRT data collected from RRST and CytAssist platforms was used for transcriptomics analysis. **b** Expression heatmap of selected markers of cartilage and bone in the epiphyseal areas, and (**c**) spatial feature plot of selected markers of bone and cartilage

Next, we aimed to characterize the transcriptome of the human growth plate and SOC. To this end, we devised a three-step strategy (Fig. 3a) to analyze the SRT data that considered (i) the low number of transcripts per spot (UMI counts), (ii) the ability to combine data and results from both platforms (RRST and CytAssist), and (iii) the lack of single-cell resolution at the spatial level. The aim of the framework is to enhance the robustness and generalizability of the results. In the first step, using an “exploration dataset” that contained four different patients per platform (Table 1), we manually annotated the epiphyseal areas (RZ, PZ, HZ, and SOC) in each section to generate a pseudo-count matrix. This matrix summed the counts from all the spots for each gene, area, and sample. This approach is analogous to a pseudo-bulk analysis in single-cell RNA-seq,^23^ which reduces false positives in subsequent statistical analyses.^24^; here, the pseudo-bulk was generated by combining the counts from spots instead of cells. Visual inspection of the first two principal components derived from the pseudo-bulk matrix showed that the four distinct areas clustered together as expected (Fig. 3a). In a second step, we identified transcriptomic markers by conducting a differential expression analysis between areas. Before investigating the results, we validated this approach by confirming that it could distinguish the zones using well-known cartilage and bone markers (Fig. 3b). As anticipated, cartilage markers (*COL2A1*, *COL9A1*, *ACAN*, *MATN3*) were restricted to the growth plate chondrocytes, and several well-described zone-specific genes were detected in the expected zones (RZ: *SFRP5*,^17^ *UCMA*;^25^ HZ: *ALPL*,^26^ *COL10A1*^27^), as confirmed by spatial feature plots for selected markers (Fig. 3c). Altogether, these results demonstrated that SRT generated meaningful results when applied to the human growth plate.

**Fig. 4:**
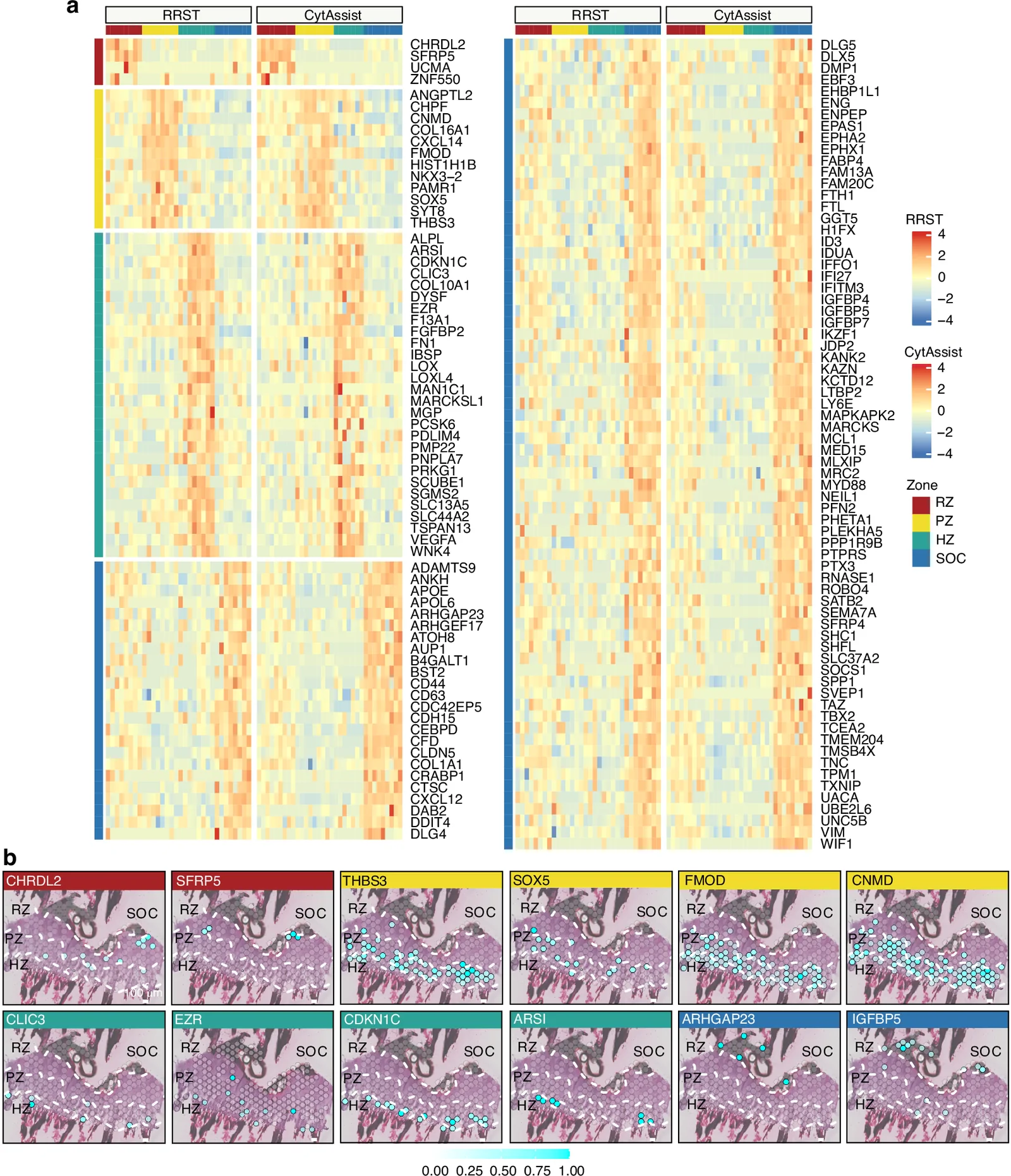
SRT reveals 138 differentially expressed genes between the three growth plate zones and SOC. **a** Expression heatmap of 138 genes significantly upregulated between both platforms in one of the areas above all others. **b** Spatial feature plot of examples of significantly upregulated genes for growth plate cartilage and SOC

Finally, we considered markers that were consistently profiled and significant across both platforms, aiming to maximize markers not associated with a single technology. We identified 138 markers that were significantly upregulated in one area above all others across all eight patients (4 in RZ, 12 in PZ, 28 in HZ and 94 in SOC) (Fig. 4a). We generated spatial feature plots for selected markers to visualize them on tissue sections (Fig. 4b), verifying that enrichment of expression occurred predominantly in the identified area.

**Fig. 5:**
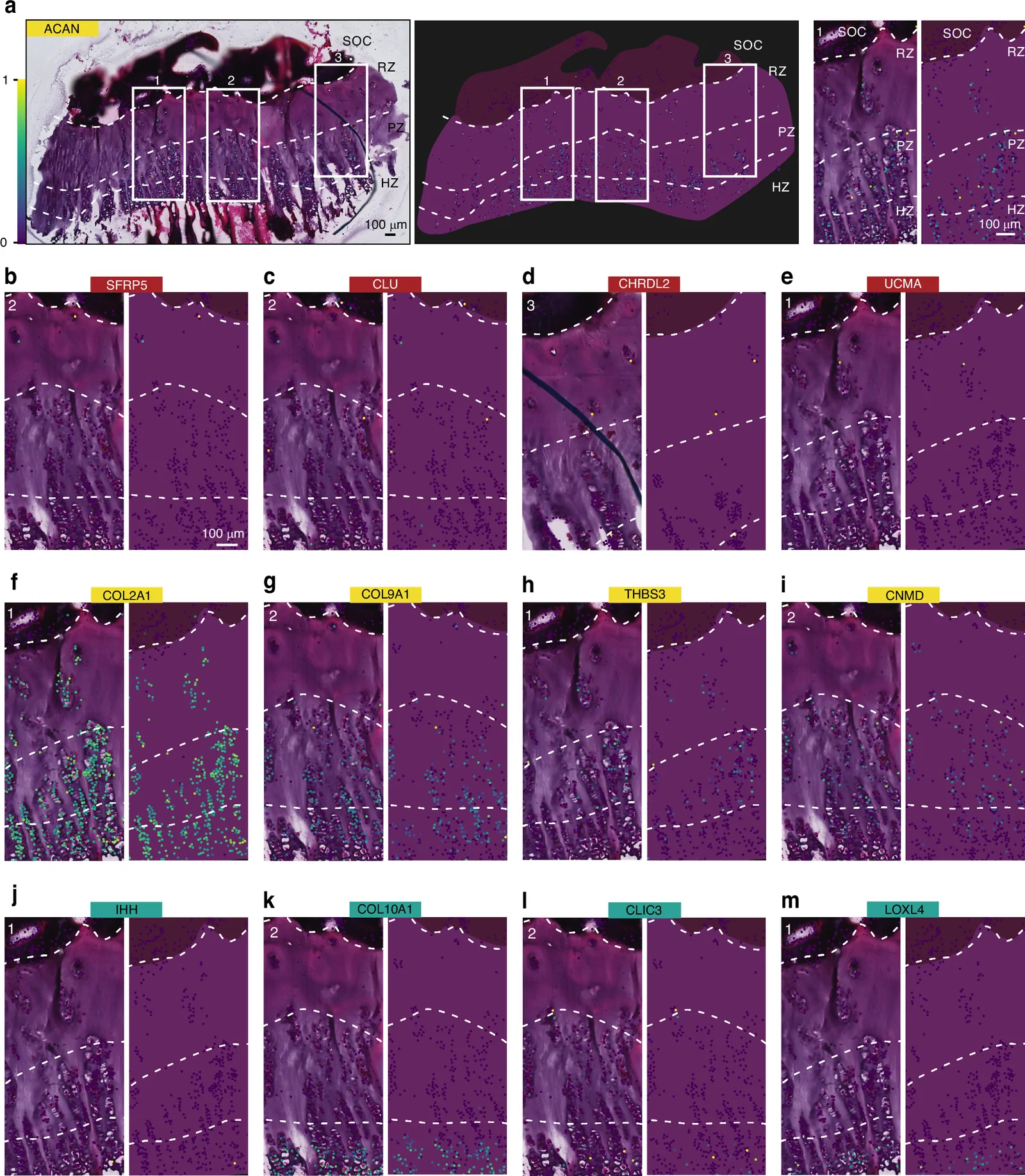
Visium HD spatial representation of known and novel zone-specific markers. **a** High resolution H&E-stained section (1), and a simplified version (2) overlaid with dots representing *ACAN* expression from Visium HD. Each square indicates a region of the sample used to visualize the expression of the different markers at higher magnification. (3) *ACAN* expression in region 1 shown in the H&E image (left) and in the simplified version (right). **b**–**m** Enlarged views of marker expression in H&E image (left) and in the simplified version (right)

We validated these findings in several ways. First, we screened the 138 identified genes in a second cohort (referred to as the “validation dataset” in the materials and methods section, table S1), which included 130 of the markers identified in the exploration dataset (Fig. S4a), and which broadly reflected the original analysis. Secondly, we selected previously unreported markers of the RZ (*CHRDL2*) and HZ (*CLIC3*), and a marker for PZ (*THBS3*) that has been previously reported in the murine PZ^28^ for visualization on tissue sections using multi-target RNAscope (Fig. S4b). These results further validated the robustness of our approach and the results. Pathway over-representation analysis (ORA) further supported the validity of the SRT profiling (Fig. S5a-d), indicating cartilage matrix assembly in the PZ, ossification in the HZ and SOC, while results for the RZ highlighted the importance of cell signaling control. Finally, we performed additional validation using Visium HD, where spatially annotated bins of 2 µm resolution were assigned to histologically segmented cells using Bin2cell.^29^ Our data revealed a high degree of specificity for growth plate markers in anticipated regions and supports the results of the Visium SRT pseudo-bulk analysis by verifying the transcriptional profiles of newly identified markers (Fig. 5a-m).

### SGMS2 is expressed in hypertrophic chondrocytes and facilitates mineralization from within growth plate-derived matrix vesicles

We screened the significantly upregulated zone-specific genes obtained from SRT (Fig. 4) to explore potential functional roles based on clinical insights. For example, *NKX3-2* was significantly enriched in the human PZ, a spatial pattern that was consistently observed using both Visium and Visium HD (Fig. S6a,b). Patients with inactivating mutations in *NKX3-2* develop spondylo-megaepiphyseal-metaphyseal dysplasia (SMMD), which impairs the growth of bones in the neck and trunk, whilst those in the appendicular skeleton become longer.^30,31^ This complicated phenotype may be because *NKX3-2* is involved in both mesenchymal condensations^32^ and the repression of chondrocyte hypertrophy.^33^ Although *Nkx3-2* has been detected in PZ chondrocytes in mouse limbs,^33^ murine models of SMMD do not completely phenocopy SMMD patients.^34^ Hence, our results showing that *NKX3-2* is specifically enriched in PZ chondrocytes in adolescent human limbs offer an important perspective on SMMD.

*WNK4* mutations lead to pseudohypoaldosteronism, involving short stature in a subset of these patients by unknown mechanisms.^35^ We found *WNK4* to be significantly enriched in HZ chondrocytes (Figs. 4; S6c,d), which could indicate that *WNK4* has a direct role in short stature via modulation of the growth plate.

**Fig. 6:**
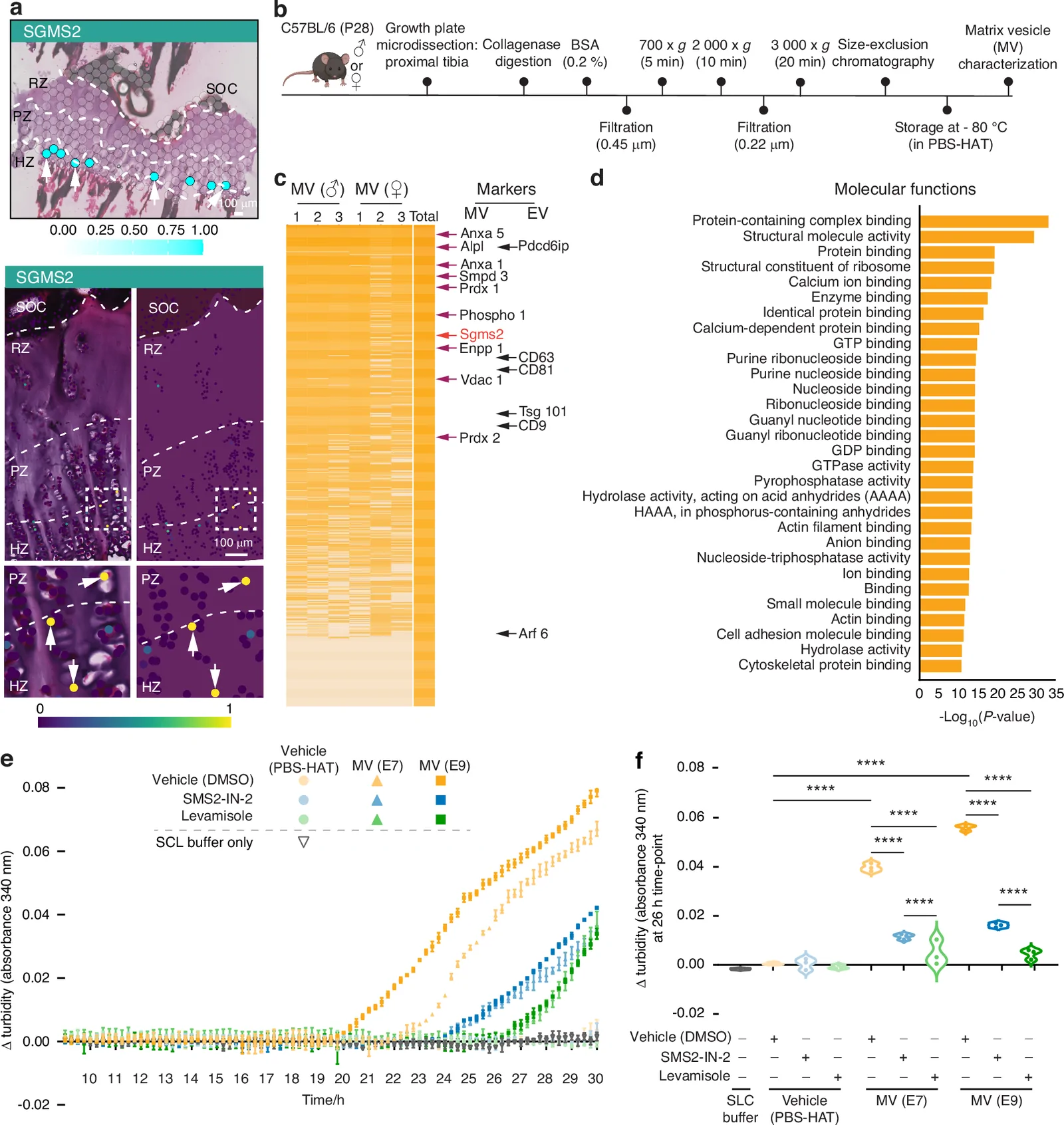
SGMS2 localizes to growth plate-derived matrix vesicles (MVs) and contributes to mineralization. **a** *SGMS2* expression determined by Visium (upper image) and Visium HD (lower image). **b** Workflow to isolate MVs from murine growth plates. **c** Heatmap of log-transformed protein abundance with selected markers indicated, and **d** molecular functions of enriched proteins. **e** MV-mineralization assay with SGMS2 inhibitor (SMS2-IN-2) or alkaline phosphatase inhibitor (levamisole). **f** Mineralization kinetics at 26 h timepoint (**e**)

*SGMS2* appeared in our panel of novel HZ markers but is also yet to be described in growth plate cartilage (Figs. 4, 6a). Missense mutations in *SGMS2*, which encodes the membrane-spanning enzyme, Sphingomyelin Synthase 2, are known to cause spondylo-metaphyseal dysplasias characterized by short stature, bowed tubular bones and under-mineralization of the skeleton.^36^ To functionally validate our SRT profiles and demonstrate the utility of our analytical pipeline, we selected *SGMS2* as a proof-of-concept candidate for further investigation. Mineral nucleation during calcification begins inside matrix vesicles (MVs), a specialized class of extracellular vesicle (EV) attached to the extracellular matrix.^37,38^ Interestingly, the enzymatic product of SGMS2, sphingomyelin, is abundant in growth plate-derived MVs^39^ and serves as a substrate for phosphate ion generation during mineral formation.^40^

To determine whether SGMS2 was localized to growth plate-derived MVs, we first developed a protocol to isolate MVs from murine growth plate cartilage (Fig. 6b), that could maintain cell viability (Fig. S7a,b). The sizes of the obtained particles were quantified by nanoparticle tracking analysis (NTA) (Fig. S7c), revealing the particles were predominantly <200 nm in diameter. Isolated particles had consistent sizes, number and particle:protein ratio between sexes (Fig. S7d-f) and collapsed spherical ultrastructures when analyzed using negative stain TEM, typical of EVs^41^ (Fig. S7g). We analyzed the presence of conventional EV markers via western blot (Fig. S7h). In accordance with the MISEV 2018 guidelines,^42^ three main categories of EV markers, growth plate-derived MV preparations contained transmembrane (Cd63) and intraluminal (Alix) EV-markers, while lacking contamination in the form of Rps6 (Fig. S7h). These data suggest that the nanoparticles isolated from murine growth plate cartilage fulfilled the criteria to be classified as EVs, representing the first method to obtain growth plate-derived EVs conducted in accordance with the Minimal Information for Studies of Extracellular Vesicles (MISEV) principles.

Interestingly, during TEM analysis, minute pieces of undigested collagen fiber could be seen on the surface of some EVs (Fig. S7g), reflecting their identity as matrix-bound particles, i.e. MVs. This notion was supported by the presence of alkaline phosphatase activity in the preparations (Fig. S7i). Next, we conducted LC-MS/MS proteomic analysis to characterize the growth plate-derived MVs, and found consistent enrichment of EV markers such as Cd63, Tsg101, Alix and Cd81 as well as MV markers including Anxa5, Alpl, Phospho1 and Enpp1 (Fig. 6c). Together, these data strongly indicate that our preparations contained MVs. Our analysis also detected Sgms2 in the preparations obtained from both male and female individuals at levels consistent with other MV markers (Fig. 6c). Pathway analysis indicated a high level of binding proteins, particularly those binding to other proteins and calcium ions, as well as pyrophosphatase activity, findings that support an enrichment of growth plate-derived MVs in the preparations (Fig. 6d).

To determine whether MV-associated Sgms2 could have a role in mineralization, we exposed these EVs to an ex vivo mineralization assay in the presence or absence of an Sgms2 inhibitor, SMS2-IN-2, or the alkaline phosphatase inhibitor, levamisole (Fig. 6e). While mineralization occurred after ~20 h in the presence of growth plate-derived MVs (at the highest dose), by 26 h, both SMS2-IN-2 and levamisole significantly delayed the onset mineralization (Fig. 6f, Table S3). Together, our results identify *SGMS2* as a novel HZ marker, which is present in MVs where it is actively involved in mineralization; these data provide a mechanistic connection between genotype and phenotype in the growing bones of patients with *SGMS2* mutations.

### Sub-populations of human resting zone chondrocytes have distinct quiescence statuses

As Visium capture spots covering SOC include multiple cell types in the bone and bone marrow, genes expressed by these cells could mask RZ markers within the growth plate in our pseudo-bulk analysis. For example, although *APOE* is described as a pan-RZ marker,^43^ our analysis indicated that it is significantly enriched in SOC versus growth plate zones (Fig. 4a). To account for this, we sought growth plate-specific RZ markers by re-analyzing the exploration dataset, excluding statistical differences with SOC from the final step of the pseudo-bulk analysis. This strategy identified 31 genes significantly upregulated in RZ versus both PZ and HZ, including the four highly selective RZ markers (*CHRDL2*, *SFRP5*, *UCMA* and *ZNF550*) and markers identified in previous studies, such as *COL15A1* (Fig. S8a). Spatial profiles of selected genes were validated by Visium and Visium HD (Fig. S8b-e). Pathway over-representation analysis (ORA) verified the importance of WNT and BMP signaling to RZ cells (Fig. S8f). We further examined spatially resolved RZ markers previously described in murine models, which were represented in our probe panel;^44^ while *APOE* had higher expression in RZ than other zones, it was not significantly enriched in our study (Fig. S8a,g-h).

The SRT profiles broadly indicated cells expressing various markers were spatially scattered across the RZ rather than being located in specific positions with respect to the surrounding areas (i.e. SOC or PZ). This was supported by the in situ hybridization experiment, which had revealed *CHRDL2* expression in chondrocytes scattered throughout the RZ (Fig. S4b). These observations suggest that the RZ may not be a homogeneous population of quiescent cells but rather could contain sub-populations dispersed across the RZ with diverse gene expression profiles.

**Fig. 7:**
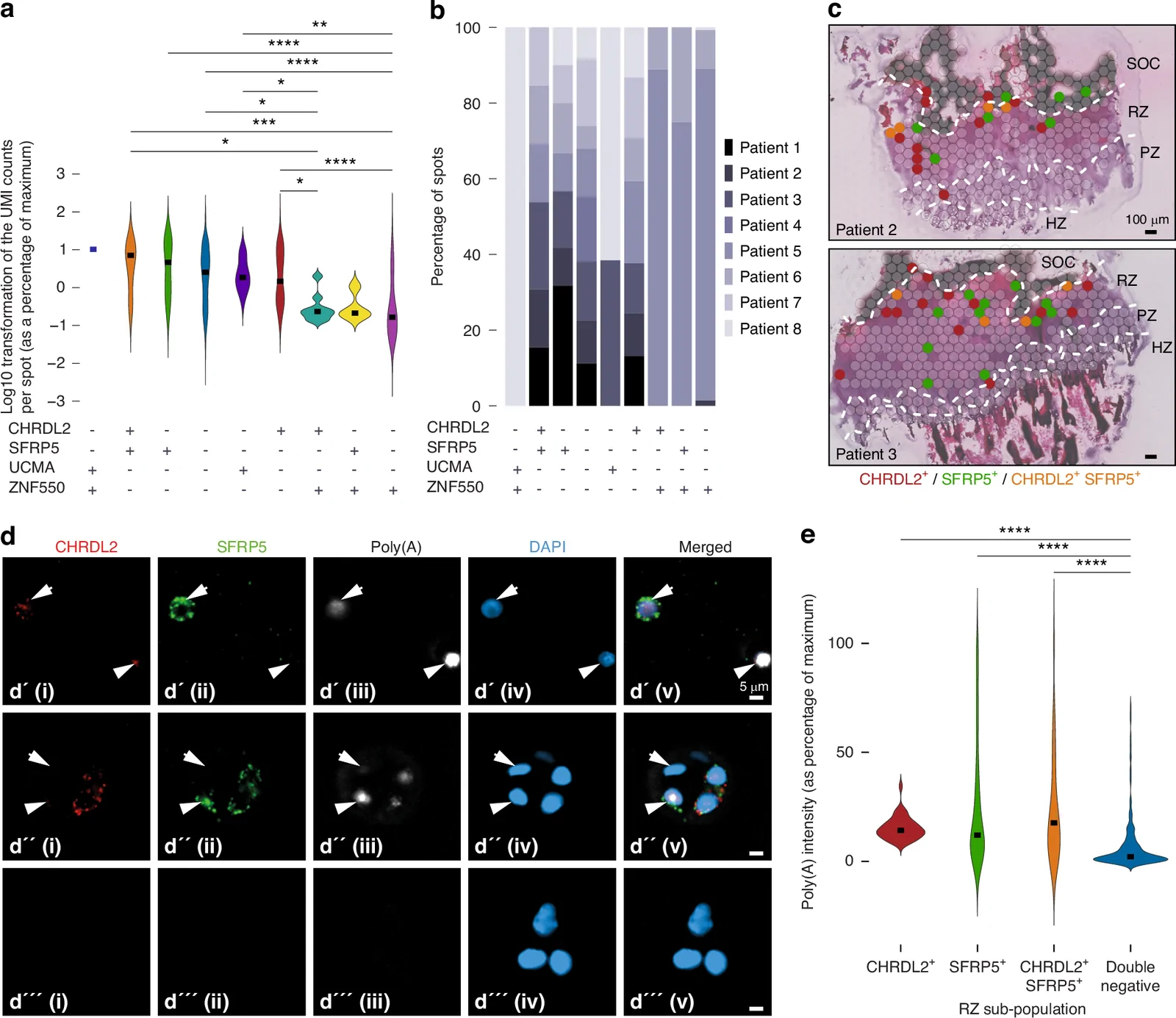
CHRDL2 and SFRP5 can be used to label distinct resting zone sub-populations. **a** Spot UMI counts in the sub-populations of the significantly up-regulated genes in RZ. **b** The abundance of the sub-populations in all eight patients. **c** Spatial feature plot of spot for single expression of CHRDL2 (in red) and SFRP5 (in green) and their co-expression (in orange). **d** High-magnification images of SFRP5/CHRDL2-double-positive cells (arrow d´), -single-positive (arrowheads, d´, d´´) or -double-negative (arrows in d´´ and d´´´). **e** Poly(A) signal intensity in the sub-populations

To explore quiescence status among the heterogeneous RZ chondrocytes, we used the 4 highly specific RZ markers obtained from SRT to identify sub-populations (Fig. S9a) and monitored their relative quiescence using spot UMI count. Intriguingly, these markers were able to split the population: spots enriched in *CHRDL2* and/or *SFRP5* tended to contain relatively high mRNA levels, whereas spots enriched in *ZNF550* contained relatively low mRNA levels (Fig. 7a). Despite applying an analysis approach that identified common markers across all 8 patients, the distributions of these sub-populations between the patients were variable (Fig. 7b), indicating an underlying differences between individuals, which did not appear to relate to genetic sex or Tanner stage (Fig. S9b).

Of the 4 highly specific RZ markers identified in SRT data (Fig. 4a), we focused on those directly involved in signal transduction: *SFRP5* (a negative regulator of Wnt signaling), and *CHRDL2* [a bone morphogenic protein (BMP) pathway antagonist], which were identified in 7 of the 8 patients (Fig. 7b). Spots containing either *CHRDL2* and/or *SFRP5* were spatially distributed throughout the RZ (Fig. 7c). Next, we elaborated on the quiescence status of these four sub-populations on the single cell level (Figs. 7d, S9c). RZ cells in which *CHRDL2* and/or *SFRP5* were detected had relatively high mRNA levels (Fig. 7e), confirming that these cells are among the least quiescent RZ cells. Furthermore, RZ chondrocytes within the same chondrons showed heterogeneity in both marker expression and total mRNA content (Fig. 7d”). Hence, *SFRP5* and *CHRDL2* label sub-populations of human growth plate chondrocytes and were not detected in deeply quiescent RZ cells.

## Discussion

### On the gene expression profiles of human adolescent growth plates

By applying complementary spatial RNA profiling platforms to adolescent human growth plate tissue for the first time, we greatly extended the understanding of its spatial transcriptomic organization and potential regulatory mechanisms. The presence of well-described markers in the anticipated growth plate zones (supplementary note 1) strongly indicates the precision of the SRT methodology and pseudo-bulk analysis approach, strengthening the validity of the less well-described and novel gene expression profiles such as *NKX3-2*, *WNK4*, and *SGMS2*. Despite the small and diverse cohort, with respect to genetic sex and Tanner stage, our analysis revealed consistent marker expression across individuals. This convergence highlights a shared transcriptional signature between individuals, although based on the source material, its relevance to the general population must be viewed with caution. Furthermore, these results confirm the relevance of gene expression profiles obtained from model organisms over past decades to human biology. These transcriptional profiles may facilitate a better understanding of diseases, improve the relevance of existing animal studies and facilitate novel strategies to treat these patients.

### On the quiescent nature of resting zone cells

Based on histological observations, some chondrocytes have been referred to as “resting” since at least the 1920s,^45^ but limited identification of features relating to their cellular quiescence has thus far been described.^46^ Our results demonstrate that human RZ chondrocytes have the characteristics of quiescent stem cells, including reduced mRNA content,^7^ predominantly nuclear mRNA localization,^7^ increased heterochromatin,^47^ lysosomal accumulation,^11^ and the ability to exit G0 when cultured ex vivo.^20,48^ With the exception of cell cycle re-entry, these characteristics have not been described in RZ cells before. Hence, the identification of these new characteristics offers a deeper understanding of RZ cell identity and reveals common traits between RZ cells and quiescent stem cells in other tissues.

### On the putative hierarchy of resting zone sub-populations

The murine RZ contains heterogeneous cells expressing a variety of markers including *Sfrp5*,^49^ *CD73*,^18,50^ *Ucma*,^25^ *Axin2*,^50^ Apoe,^43^ *Clu*,^50^ PTHrP,^13,50^ and *Foxa2*,^14^ among others.^17^ The current consensus is that the RZ chondrocytes include skeletal stem and progenitor cells as the *de facto* stem cells have not yet been identified. Little is known about hierarchy among the populations except for tracing experiments suggesting that *Foxa2*-expressing RZ chondrocytes lie upstream of the PTHrP-positive population.^14^ Our own results demonstrate that, like mice, human RZ chondrocytes are also heterogenous, based on their differential expression of *CHRDL2*, *SFRP5*, *UCMA* and *ZNF550*. To explore the potential hierarchy of these populations, we assessed their quiescence level by UMI count or polyadenylated RNA intensity. Both approaches revealed that RZ sub-populations had heterogenous RNA content, indicating that different sub-populations had varying quiescence statuses. Nevertheless, we determined that sub-populations of RZ cells expressing *CHRDL2* and/or *SFRP5* were located throughout the human RZ and that cells within these sub-populations contained significantly more mRNA than *CHRDL2*/*SFRP5*-double-negative cells. As individual cells within each chondron were heterogenous for total mRNA, *CHRDL2* and *SFRP5* levels, it is possible that local regulation within each chondron maintains the RZ, which would align well with our understanding that repressed BMP- and Wnt signaling maintains the RZ in rodents.^17^ Further investigation aimed at more precisely stratifying RZ sub-populations using an expanded panel of markers, understanding the degree of local control within and between chondrons, and unpicking the precise roles of the identified genes beyond their use simply as markers, would significantly advance our understanding of RZ maintenance during postnatal growth.

While post-natal growth in mice is fueled by skeletal stem cells,^18^ mouse and human RZ cells have similar characteristics and marker expression,^17^ and it is thought that the general mechanisms of growth between mice and humans are similar,^51^ it is important to emphasize that direct evidence of an epiphyseal stem cell population and niche is lacking. Human growth plates contain relatively large RZs in comparison to rodents, and some of these cells may fulfil roles that have yet to be defined.^52^ Further research is required to definitively test if RZ cells, or a specific subset of them, fulfill the necessary criteria to represent bona fide stem cells in human growth plates.

### On the potential role of SGMS2 in skeletal mineralization

SGMS2 catalyzes the conversion of ceramide to sphingomyelin, a metabolite which is then further catalyzed to provide phosphate ions during matrix mineralization.^40^ Pekkinen and colleagues^36^ recently explored the pathologies and mechanisms underpinning several SGMS2 heterozygous variants, making some key findings: while under-mineralization appeared in individuals with nonsense and missense mutations, patients with missense mutations had stronger phenotypes, including bowing of the long bones during growth. In vitro analysis determined that while the enzymatic function of SGMS2 in missense variants p.Ile62Ser and p.Met64Arg was not impaired, the resultant proteins accumulated in the endoplasmic reticulum rather than the plasma membrane. Our discovery that endogenous Sgms2 is located in growth plate-derived MVs where it facilitates mineralization, could provide a key missing piece of the puzzle: it suggests that the localization of SGMS2-mediated sphingomyelin synthesis within the extracellular matrix is necessary for sufficient tissue mineralization. Additionally, while classical EV biogenesis pathways are mediated by the endosomal sorting complex required for transport (ESCRT) or tetraspanins, EVs can also be generated through ceramide-dependent mechanisms,^53^ potentially placing SGMS2 in dual roles in both biogenesis and function in MVs. Hence, future research investigating MVs in these patients may further elaborate the underlying mechanisms.

### Limitations

First, all biopsies were obtained from adolescents undergoing surgery for idiopathic tall stature. Although these individuals were otherwise healthy, their growth plates may differ from that of the general population. Second, while the sample size reflects a relatively large study in relation to human growth plate, it nonetheless restricts statistical power and limits stratified analysis across sex, age, and pubertal stages. In relation to the results obtained from RRST/CytAssist (using the standard Visium platform), several limitations should be noted. First, the spot size in the SRT of 55 µm diameter precluded single cell resolution. Second, due to the relatively low levels of transcription, we needed to devise a robust strategy to detect statistically significant differences between the populations which precluded the unbiased stratification of cells within the entire growth plate; this issue also introduces potential bias in sub-population identification. Furthermore, these SRT methods are probe-based and although covering the whole transcriptome many growth plate-relevant genes were missing from one or both panels (e.g. *IHH*,^54^ *PTHLH*^55^). Additionally, although our SRT analysis describes significantly differentially expressed genes across all eight patients, the results were not sufficient to determine those specific to, for example, genetic sex or pubertal stage, as determined by our power analyses.

While our growth plate-derived MV preparations contained key MV protein markers (e.g., Alpl, Enpp1, Phospho1), alkaline phosphatase activity, and mineralization potential, these assays are at the bulk EV level, and we cannot exclude that non-mineralizing EVs are also present in our MV preparations. Furthermore, while MVs may vary in size from 30-1 000 nm in diameter,^37^ our isolation method obtained only those in the range of 100–200 nm in diameter.

In summary, we greatly elaborated upon the human growth plate transcriptome, allowing us to identify novel zone-specific markers, new primary growth disorders, and candidate pharmacological targets. Furthermore, our results revealed that the RZ contains chondrocytes that display characteristics of adult stem cells and sub-populations of varying quiescence status. Together, these findings can facilitate improved diagnosis and treatment strategies of patients with skeletal growth disorders.

## Materials and methods

### Study design

In this study, we aimed at deciphering the transcriptional profiles of the human growth plate. We selected biopsies containing growth plate and SOC for SRT. We applied the RRST platform, a method we previously optimized for use with undecalcified cartilage tissue, and to improve sequencing results, complemented this with CytAssist. We selected the four patients from each platform with the highest median UMI count per spot, resulting in an exploration dataset of four male and four female patients, two per platform, to reduce the risk of bias. All other sequenced sections using RRST were added to the Validation cohort. Spots were manually assigned into zones by a trained observer. No statistical method was used to pre-determine sample size. The experiments were not randomized, and the investigators were not blinded to allocation during experiments and outcome assessment. Animal experiments were performed in accordance with the ARRIVE guidelines.^56^

### Ethics declaration

The collection of human growth plate samples was approved by the local human ethics committee (KI forskningsetikkommitté Nord vid Karolinska Sjukhuset), ethical permit 97/214. Informed consent was obtained from participating subjects and their caregivers before enrolment in the study, which was performed according to the Declaration of Helsinki. Mouse bone samples were collected according to ethical permit 16673/2020, approved by Stockholm’s animal experiment ethics committee (Stockholms djurförsöksetiska nämnd).

### Human growth plate sample information and collection

Human growth plate samples were collected from constitutionally tall-stature patients undergoing epiphyseal surgery (epiphysiodesis) to reduce bone growth at the Karolinska University Hospital. To be eligible, for treatment a predicted adult height of at least 186 cm for girls or 200 cm for boys (corresponding to 3 standard deviations above mean height), at least 8 cm of predicted growth remaining, and a strong desire to undergo surgery must exist. Immediately after collection, biopsies from distal femora and proximal tibiae were transferred into DMEM-F12 medium (phenol red free [21041025; Gibco]) on ice and transported directly to the laboratory. Under microscopy, biopsies in DMEM/F12 were dissected on ice into slices of approximately 1 mm thickness, using a scalpel. For SRT experiments, fresh-frozen samples were prepared by placing each slice into Optimal Cutting Temperature (OCT) compound (4583; Tissue-Tek) within a cryomold (62534-10; Tissue-Tek), which was rapidly frozen in a hexane (296090-1; Sigma-Aldrich) bath that had been pre-cooled in a tank of absolute ethanol (01399; Histolab) containing dry-ice (carbon dioxide ice). For RNAscope, each slice was fixed in pre-cooled 4% formaldehyde (64833; Merck)/PBS for 48 h at 4 °C. Samples were then placed into 30% sucrose (S-2885; AG Scientific) overnight and embedded in OCT in cryomolds. Samples were stored at −80 °C prior to cryo-sectioning.

### RRST gene expression library preparation

Fresh-frozen human growth plate slices were cryo-sectioned in a CryoStar NX70 (957070; Epredia) at 10 µm thickness, placed onto glass slides from Visium Spatial for FFPE Gene Expression kit (PN-1000338; 10x Genomics), and stored at -80°C before processing. Each Capture Area contained section(s) from 1 patient. Spatial gene expression libraries were generated following RRST Gene Expression protocol, as previously reported.^6^ Final libraries were purified and assessed using an Agilent Bioanalyzer (Agilent 2100 Bioanalyzer system) and quantified using the Qubit Fluorometric quantification (ThermoFisher) before sequencing on NextSeq2000 (Illumina) at a depth of 25 000 reads per tissue covered spot on Capture Area. Read 1 (28 bp [base pairs]) encodes for the Spatial barcode and Unique Molecular Identifier (UMI) and Read 2 (50 bp) were used to sequence the ligated probe insert.

### CytAssist gene expression library preparation

Fresh-frozen human growth plate slices were cryo-sectioned at 10 µm thickness, placed within a 6.5 mm Capture Area on SuperFrost Ultra Plus GOLD Adhesion slides (11976299; Thermo Fisher Scientific) as per^6^, and stored in −80 °C freezer. Each Capture Area contained sections from two patients. The tissue slides were removed from −80 °C freezer, fixed and stained as in RRST protocol, as previously reported. CytAssist gene expression libraries were generated using Visium CytAssist Spatial Gene Expression for FFPE kit (PN-1000520; 10x Genomics) following 10x Genomics Visium Spatial Gene expression protocol (User Guide, CG000495 Rev D). Final libraries were purified and assessed using an Agilent Bioanalyzer (Agilent 2100 Bioanalyzer system) and quantified using the Qubit Fluorometric Quantification (ThermoFisher) before sequencing on NextSeq2000 (Illumina) at a depth of 25 000 reads per tissue covered spot on the capture area. Read 1 (28 bp) encodes for the Spatial barcode and UMI and Read 2 (50 bp) were used to sequence the ligated probe insert.

### Data pre-processing

Sequenced libraries were processed using Space Ranger software (version 1.3.1 for RRST data and version 2.0.1 for CytAssist data, 10x Genomics). Reads were aligned to the pre-built human reference genome the GRCh38, provided by 10x Genomics (version refdata-gex-GRCh38-2020-A). h5 files and image data were obtained.

### Spot-level annotation

Spots from all Capture Areas were annotated on a spot-by-spot basis using Loupe Browser version 6.2.0 (10x Genomics). Based on tissue histology, spots were manually defined into areas: SOC, RZ, PZ, HZ, and POC. Spots lacking cells were excluded. Since human growth plate slices did not always contain entire growth plates, we prioritized the use of sections that contained the complete SOC and RZ with SRT. Of those used to generate the exploration dataset, 29.4% of tissue sections lacked POC (Table 2), which was excluded from downstream analyses.

### Statistical analysis

Normality was assessed using the Shapiro-Wilk test; where normality could be assumed, parametric tests were performed (Figs. 1d, 2f, 6f, S7b, S7d, S7e, S7f), otherwise non-parametric tests (Figs. 1f, 2a, 2c, 2d, 2g, 7a, 7e, S1b, S1c, S2d, S3c, S7i) were applied. Data were processed using RStudio software version 4.1.2 (2021-11-01) or GraphPad Prism version 10.2.3. Statistical analyses were performed using GraphPad Prism Software 9.

### Supplementary information

Supplementary information accompanies the manuscript on Bone Research website http://www.nature.com/boneres.

## Supplementary information

Supplementary information

## Data Availability

All data associated with this study are present in the paper or the Supplementary Materials, whereas code and data used in spatially resolved transcriptomics analyses (Visium and Visium HD) are available online: https://github.com/anarl/spatial_bone_growth. Original images can be obtained from the corresponding authors upon reasonable request.
