# Supplementary material for: Human growth plates house resting zone sub-populations with features of quiescent stem cells
Source: Bone Res. 2026 Aug 5;14:79. doi: 10.1038/s41413-026-00564-y (PMC13438842; doi:10.1038/s41413-026-00564-y)
Supplement: Supplementary file 1 [file 41413_2026_564_MOESM1_ESM.docx]

**Supplementary information**

**Supplementary Materials and Methods**

Image capture and analysis

Bioinformatics analysis

Transmission electron microscopy to assess cell ultrastructure

Triple labelling of human growth plate biopsies with thymidine analogues

Visium HD library preparation

Matrix vesicle isolation from rodents

Matrix vesicle characterization

Transmission electron microscopy (TEM) for matrix vesicle ultrastructure

Liquid chromatography electrospray ionization tandem mass spectrometry (LC-ESI-MS/MS)

Peptide and protein identification

Mineralization assay

Collection of mouse tissues for histological analyses

RNAscope *in situ* hybridization

RNAscope *in situ* hybridization and EdU detection

Image capture and analysis specific to supplementary figures

**Extended legends for main figures**

**Supplementary notes**

Supplementary note 1 – Discussion regarding anticipated genes identified within human growth plates by SRT.

**References within Supplementary Materials**

**Supplementary Figures**

Figure S1. Detailed assessment of SRT application to human growth plate biopsies

Figure S2. mRNA in mouse resting zone chondrocytes has predominantly nuclear localization

Figure S3. Low mRNA levels are a strong indicator of LRCs in mice

Figure S4. SRT validation using an additional dataset and *in situ* hybridization

Figure S5. Over-Representation Analysis on zone-specific markers.

Figure S6. Zone-specific enrichment of clinically relevant genes in the human growth plate

Figure S7. Characterization of matrix vesicles (MVs) isolated from murine growth plate cartilage

Figure S8. Identification and spatial validation of RZ-specific markers in the murine growth plate

Figure S9. Quantification of sub-populations of human RZ chondrocytes

**Supplementary** **Table**

Table S1. SRT Validation dataset

Table S2. Protein abundance in matrix vesicles isolated from growth plates of male and female mice.

Table S3. Results of statistical testing for mineralization assay containing all comparisons.

**Supplementary Materials and Methods**

**Image capture and analysis**

Fluorescent images were acquired using Zeiss laser scanning microscopes (LSM) 700, 800 or 980. Maximal intensity projections (MIP) were routinely generated from the acquired Z-stack images. Depending on the purpose and user, images were analyzed using ImageJ,^58^ CellProfiler (https://cellprofiler.org/) or QuPath (<https://github.com/qupath>).

For the quantification of mean poly(A) signal intensity per nucleus as a percentage of maximum value (Figs. 1f, 2g, S2c and S3c), the microscopy MIP files were loaded into ImageJ, and the following steps were used: channels were split, DAPI channel was selected to set its threshold and convert to mask and nuclei was identified. Next, poly(A) channel was selected to measure the mean signal intensity in nuclei. The same settings were used for every sample. The values for each zone were saved as separate csv files and taken into RStudio to analyze. We normalized RNA values within the growth plate of each individual, using its own maximum value (Figs. 1f, 2g, S7e, S2c and S3c). The ggplot package was used to generate graphs (Figs. 1f, 2gi, S7e, S2c and S3c). For the quantification of nucleus/cytoplasm poly(A) signal ratio (Figs. 2a, S2d), the microscopy MIP files were loaded into CellProfiler and the following steps were used: multichannel image was split and converted to grayscale, the nucleus was segmented by selecting DAPI channel, the cell was identified by expanding from nucleus edge, the cytoplasm was identified by subtracting nucleus from cell. The poly(A) signal intensity was measured in the nucleus and cytoplasm of each individual cell. The same settings for object size and method were used for every sample. The values for each zone were saved as separate csv files and taken into RStudio to analyze. The ggplot package was used to generate graphs (Figs. 2a, S2d).

For the quantification of triple labeled sections of human growth plate (Fig. 2f), the microscopy MIP files were loaded into ImageJ, and the following steps were used to count the absolute number of labeled cells with BrdU analogues: channels were split, DAPI channel was selected to set its threshold and convert to mask, and nuclei were segmented and counted. Each channel was separately thresholded and labeled nuclei were identified and counted. The data were loaded to GraphPad Prism version 10 to plot and analyze.

For the quantification in Fig. 4, microscopy MIP files were loaded into QuPath and values of mean poly(A) intensity per nucleus obtained following cell detection. A manual assessment of *CHRDL2* and *SFRP5* signals (in which positive cells either had a large positive region or more than two puncta) was used as a guide to select thresholding values for *CHRDL2* and *SFRP5* channels. We applied stringent automated thresholding by signal intensity, which could be an underestimate of positive cells, as some cells contained one or two individual puncta, and not all signal was confined to the nucleus.

**Bioinformatics analysis**

The h5 files obtained from Space Ranger were converted into Seurat objects. The table containing the area annotation was added to the metadata for each sample and section. Finally, Seurat objects were merged to obtain a Seurat object containing all the samples. For each section, the raw reads coming from spots labelled as SOC, RZ, PZ and HZ were summed up generating a new count matrix (pseudo-bulk approach). RRST and CytAssist samples were split into two count matrices, and a metadata table was generated for each platform. Analyses for each sample were done independently following the same pipeline. First, only genes with at least 10 reads among all the samples were kept for further analysis per platform. Patient-based batch correction was performed using ComBat_seq.^60^ Normalization and differential expression analysis were performed using DESeq2 separately for each platform.^61^ Pairwise comparisons between the different areas were performed and for each pairwise comparison genes with a *p*-value < 0.2 and that were either up- or down-regulated in the analysis associated with both platforms were combined using the fisher test.^62^

A gene was considered a candidate marker of an area (SOC, RZ, PZ or HZ) if it was up-regulated in the specific area compared to the other three areas and has a False Discovery Rate (FDR) adjusted Fisher *p* value < 0.05 in the three comparisons; based on the Benjamini Hochberg threshold. For validation purposes, the same pseudo-bulk strategy was followed in the validation dataset. In this case we only checked the differential expression of the 138 markers genes found in the exploration dataset. These samples were all sequenced on RRST platform; similar normalization and differential analysis were conducted. Genes were considered significant with an FDR < 0.05. Only 130 of the 138 genes are presented in Fig. S4a since the other 8 genes did not meet the expression level threshold in the validation dataset. The RZ markers within the growth plate were re-analyzed by selecting those ones significantly up-regulated in RZ versus HZ and PZ (log2FC > 0 and fisher p-value < 0.1) and having a log2FC > 0 in RZ versus SOC.

**Transmission electron microscopy to assess cell ultrastructure**

A human growth plate slice was further cut during dissection to approximately 1.5 mm in width, fixed in 2.5 % glutaraldehyde (20105; Ladd Research industries) in 0.1 M phosphate (pH 7.4) for 1 hour at room temperature, and stored at 4 °C until further processing (approximately 36 hours). Next, samples were rinsed in 0.1 M phosphate buffer and then post-fixed in 2 % osmium tetroxide (O018; TAAB) /0.1 M phosphate buffer, (pH 7.4) at 4 °C for 2 hours. After stepwise dehydration in ethanol and acetone, the tissue was embedded in LX-112 resin (Ladd). An EM UC7 (Leica) was used to generate Ultrathin sections (approximately 80-100 nm), which were subsequently contrasted with uranyl acetate (U001; TAAB), followed by lead citrate (7398; Merck), and finally examined using a HT7700 transmission electron microscope (Hitachi High-Technologies) at 80 kV. A 2kx2k Veleta CCD camera (Olympus Soft Imaging Solutions) was used to acquire digital images. The proportion of heterochromatin on the nuclear region of electron microscopy images was assessed using ImageJ^63^ as previously reported.^64^ In brief, the nuclear membrane was delimited and subjected to an intensity threshold on filtered images by Gaussian blur (radius = 2 pixels). The percentage of heterochromatin is reported as the heterochromatin area in the total nuclear surface. Using electron microscopy images, single-membrane-bound bodies containing electron-dense material with a round- or elongated-like ultrastructure were defined as lysosomes, according to standard criteria.^65^ The number of lysosomes was assessed by cell.

**Triple labelling of human growth plate biopsies with thymidine analogues**

Individual human growth plate slices were grown in one well of a 24-well plate (83.3922; Sarstedt) with 2 ml of culture medium containing DMEM/F12 (11320033; Gibco) supplemented with 50 µg/ml ascorbic acid (A9560; Sigma-Aldrich), 0.2 % BSA (05470-5G; Sigma-Aldrich) and 50 µg/ml gentamicin (11530506; Fisher Scientific). EdU was added on day 1 at 1:1000 of 25 mg/mL stock solution and incubated overnight. IdU (I7125-5Gl; Merck) was added on days 3 and 6 at 1:1000 of 250 mM stock solution. CldU (C6891; Merck) was added on days 9, 11, 13, and 15 at 1:1000 of 15 mM stock solution. On days 2 and 8, wells were rinsed with PBS and slices were placed in culture medium without thymidine analogues. Explants were maintained in a humidified atmosphere (37°C, 5% CO_2_) within an incubator (51033595; Thermo Scientific). Explants were typically placed in culture within approximately two hours of surgery. On day 17, the explants were briefly rinsed in PBS and fixed in pre-cooled 4 % PFA/PBS overnight at 4 °C. Subsequently, the samples were incubated in 30 % sucrose overnight at 4 °C, and the following day they were embedded in OCT in cryomolds. Samples were stored at -80 °C before cryo-sectioning.

The detection method was used as described previously,^66^ with modifications made because some fluorescent antibodies were no longer commercially available. CldU was visualized using anti-BrdU antibodies conjugated to Alexa Fluor 488 ([clone BU1/75 (ICR1)] 220074l; Abcam). The anti-IdU antibodies were detected using donkey anti-mouse antibodies conjugated to Alexa Fluor 555 (150106; Abcam), and EdU was detected using Alexa Fluor azide 647.

**Visium HD library preparation**

Fresh-frozen human growth plate samples were cryo-sectioned at 10 µm thickness, placed within a 6.5 mm Capture Area on SuperFrost Ultra Plus GOLD Adhesion slides (11976299; Thermo 436 Fisher Scientific) following 10x Genomics Tissue Preparation Guide (Demonstrated Protocol, 437 CG000518 Rev C), and stored in -80 °C before processing. The tissue slides were retrieved from the -80 °C freezer and placed on a thermocycler pre-heated to 37 °C for 1 min, fixed and stained as in RRST protocol, as previously reported.^59^ Visium HD gene expression libraries were generated using Visium HD, Human Transcriptome, 6.5 mm (PN-1000675; 10x Genomics), as described. ^59^ Final libraries were purified and assessed using an Agilent Tapestation and quantified using the Qubit Fluorometric Quantification (ThermoFisher) before sequencing on NextSeq2000 (Illumina) at a depth of 275 million read pairs per tissue coverage on the Visium HD Capture Area. The Read 1 (43 bp) encodes for the Spatial barcode and UMI and Read 2 (50 bp) were used to sequence the ligated probe insert. Sequenced libraries were processed using Space Ranger software version 3.1.2 for Visium HD data. One patient per capture area was selected for downstream analysis. Visualization of gene expression was performed as follows: segmentation was performed with Bin2cell ^67^ in which DAPI staining was used by the software to detect individual nuclei. The volume ratio algorithm was used to extend the nuclei to cells, and underlying bins (2um resolution) were assigned to the given segmented cell.

**Matrix vesicle isolation from rodents**

Under aseptic conditions, C57BL/6 male or female mice were euthanized at post-natal-day 28 followed by removal of the skin and dissection of the hind limbs. Legs were rinsed in pre-cooled (4 °C), 0.22 µm PBS (1X) and subsequently transferred to pre-cooled (4 °C) DMEM/F-12 supplemented with GlutaMAX™ (Gibco, catalogue number: 31331093) and 0.1 mg/ml gentamicin (Gibco, catalogue number: 15750045), hereafter called dissection medium. Next, muscle and connective tissue were gently removed to expose the knee joint under a stereomicroscope (Nikon SMZ645), using fine forceps (VWR. Cat. No: OUTI5-DURAX) and a scalpel (Swann-Morton. Cat. No: DS-10). The tibia was carefully separated from the articular capsule and then the articular cartilage was removed followed by a straight cut at the primary spongiosa region. The microdissected growth plates were placed in pre-cooled (4 °C) dissection medium until processing. Thereafter, growth plates were gently minced in small fragments and digested in 0.15 % collagenase II (Worthington Biochemical. Cat. No. LS004174) at 37 °C for 45 min. Note that the growth plate fragments in collagenase II were gently vortex once before incubation. The enzymatic digestion was then stopped by adding 0.2 % bovine serum albumin (BSA) prepared in dissection medium (0.22 µm filtered). To separate the undigested growth plate fragments from the suspension, the samples were filtered through a 45 µm cell strainer (Sigma-Aldrich. Cat. No. CLS352340). This filtrate was centrifuged at 700 × g for 5 min at 4 °C followed by 2,000 × g for 10 min at 4 °C, thus removing cell debris. Of note, the pellet obtained after the initial centrifugation (700 × g for 5 min) was used to evaluate chondrocyte growth plate viability using Trypan Blue (Bio-Rad, Cat. No. 1450021) and quantified with a TC20 Cell Counter (Bio-Rad). The cleared samples were further filtrated (0.22 µm) and then concentrated using 100 kDa Amicon Ultra centrifugal filters (Millipore, catalogue number: UFC9100) at 3,000 × g for 30 min at 4 °C. Concentrated samples were subsequently subjected to size-exclusion chromatography (70 nm, Izon Science) we previously described.^1^ EVs were stored in Maxymum Recovery polypropylene 1.5 mL tubes (Corning, Axygen Maxymum Recovery, catalogue number: MCT-150-L-C) and in 0.22 µm filtered phosphate-buffered saline (PBS) supplemented with HEPES (ThermoFisher, catalogue number: 15630-080), bovine serum albumin (Sigma–Aldrich, catalogue number: 05470), and trehalose [Sigma–Aldrich, D-(+)-Trehalose dihydrate, catalogue number: T0167-25G], referred to as PBS-HAT (1X).^68^ Note that the human albumin used in the original protocol was replaced with bovine serum albumin, as we have previously described.^69^ Note that growth plate microdissection and matrix vesicle isolation were performed sequentially on the same day to preserve EV integrity and functionality, as generally suggested for EV studies. Note that growth plate microdissection and matrix vesicle isolation were performed sequentially on the same day to preserve EV integrity and functionality, as generally suggested for EV studies.^70^ The EV-related methods were performed in accordance with the MISEV 2018 principles;^70^ to contribute to the standardization of the EV field, we have submitted all relevant data of our experiments to the EV-TRACK knowledgebase (EV-TRACK ID: EV250118).^71^

Cell viability of chondrocytes derived from growth plates was assessed with Trypan Blue (Bio-rad. Cat. No: 1450021) and obtained in a Cell counter (*Bio*-*Rad* TC20).

**Matrix vesicle characterization**

Particle concentration and size were assessed by nanoparticle tracking analysis using a ZetaView® instrument (PMX-230-Z-488/640, Particle Metrix) with a Particle Metrix Software Suite 1.3. Following the manufacturer's instructions, the equipment was rinsed for 30 seg with 0.22 µm filtered PBS (1X) and calibrated using 100 nm polystyrene beads standard solution (700074, Particle Matrix) diluted in 0.22 µm filtered PBS (1X) at 1/250,000. Next, the instrument was rinsed with 5 ml of 0.22 µm filtered PBS (1X). Thereafter, samples were measured using the “EV scatter T” mode under constant parameters, including temperature (25 °C), laser at 488 nm, sensitivity (82) and shutter (100). Of note, all 12 cell positions of the instruments were analyzed. Note that the equipment was cleaned with 5 ml of 0.22 µm filtered PBS (1X) between samples. Measurements were visualized and downloaded as PDF files using the ParticleExplorer (PEX) software package (Particle Metrix).

Protein quantifications and particle ultrastructure were analyzed using the microBCA kit and transmission electron microscopy, respectively, whereas EV markers (CD63 and ALIX) and contaminants in the form of RPS6 were assessed by western blot using the same method and cell lysate preparations (i.e. for the control sample), as we recently described.^69^

**Transmission electron microscopy (TEM) for matrix vesicle ultrastructure**

EVs were prepared for TEM using negative staining as we previously reported.^69^ Briefly, 5 µL of each EV sample was applied to a glow-discharged, carbon-coated grid and incubated for 1 minute. Excess liquid was gently blotted with filter paper, followed by a 7-second incubation with 5 µL of 1% uranyl acetate in water for contrast enhancement. After removing the excess stain with filter paper, the grids were allowed to air dry. Imaging was performed using a Talos 120C G2 transmission electron microscope (Thermo Scientific) operating at 120 kV and equipped with a CETA-D camera.

**Liquid chromatography electrospray ionization tandem mass spectrometry (LC-ESI-MS/MS)**

Online LC-MS analysis was conducted using a Dionex UltiMate™ 3000 RSLCnano System coupled to an Orbitrap Exploris 480 mass spectrometer (Thermo Scientific). An aliquot of each sample was suspended in LC mobile phase A. Equal peptide amounts derived from each sample (male and female) were injected into the LC–MS/MS system to minimize technical variability; this approach ensures that differences observed across samples reflect biological variation rather than disparities in sample concentration. Peptides were first trapped on a C18 guard desalting column (Acclaim PepMap 100, 75 µm × 2 cm, nanoViper, C18, 5 µm, 100 Å) and subsequently separated on a 25 cm C18 column (Aurora Ultimate, C18, 1.7 µm, 25 cm × 75 µm). The mobile phase (nano capillary) consisted of solvent A (0.1 % formic acid in water) and solvent B (0.1 % formic acid in acetonitrile). Using a constant flow rate of 0.25 µL min^−1^, a gradient was applied, starting at 6 % B and increasing to 43 % B over 180 minutes, followed by a sharp rise to 100 % B over 5 minutes.

Data acquisition was performed with FTMS master scans at a resolution of 120,000 (400–1200 m/z). Data-dependent MS/MS (60,000 resolutions) was performed on the top five most abundant ions using higher energy collision dissociation (HCD) with a normalized collision energy of 30 %. Precursor ions were isolated with a 2 m/z window. Automatic gain control (AGC) targets were set to 1 × 10⁶ for MS1 and 2 × 10⁵ for MS2, with maximum injection times of 100 ms for both MS1 and MS2. The total duty cycle duration was approximately 2.5 s. Dynamic exclusion was employed with a duration of 45 s, and precursors with unassigned or charge state 1 were excluded. An underfill ratio of 1 % was applied.

**Peptide and protein identification**

Orbitrap raw MS/MS files were converted to mzML format using msConvert from the ProteoWizard tool suite. Spectral data were analyzed using MSGF+ (v2020.03.14)^72^ and Percolator (v3.5)^73^ for target-decoy Percolator scoring. All searches were performed against the murine protein database Ensembl 113 within a proteomics workflow (<https://github.com/lehtiolab/ddamsproteomics>, v2.18), which was constructed using the Nextflow workflow tool (v22.10.5). MSGF+ search parameters included a precursor mass tolerance of 10 ppm, fully tryptic peptides, a maximum peptide length of 50 amino acids, and a maximum charge state of 6. Carbamidomethylation of cysteine residues was set as a fixed modification, while oxidation of methionine residues was considered as a variable modification. MS1 feature detection and quantification were conducted using Hardklor (v2.3.0) and Kronik (v2.20). Peptide spectrum matches (PSMs) identified at a 1 % false discovery rate (FDR) were used to assign gene identities. Protein quantification was based on the average intensity of the top three highest-intensity peptides, with peptide MS1 quantification defined by the 'Best Intensity', representing the intensity value at the apex of the feature elution profile.

Protein FDRs were calculated using the picked-FDR method, with gene symbols as protein groups, and restricted to 1 % FDR.^74^ For peptide identification, a precursor ion mass tolerance of 10 ppm and product ion mass tolerances of 0.02 Da for HCD-FTMS and 0.8 Da for CID-ITMS were applied. The search algorithm accounted for tryptic peptides with a maximum of two missed cleavages, with carbamidomethylation (C) as a fixed modification and oxidation (M) and deamidation (N) as variable modifications. The proteomic data were filtered and processed for bioinformatic analysis in the following way. Proteins in which more than 50 % of values were missing were filtered out, and the remaining missing data was imputed using a random forest method, MissForest,^75^ implemented in Python. The log-transformed resulting protein abundance data was used for plotting heatmaps. The molecular function plot was generated using the proteomic data against Ensembl 113 (<http://oct2024.archive.ensembl.org/index.html)> protein list for mouse species, using Gorilla tool.^76,77^

**Mineralization assay**

The mineralization assay was performed according to the following publication.^78^ Briefly, the synthetic cartilage lymph (SCL) buffer was prepared freshly, which is composed of 1.83 mmol L^−1^ NaHCO_3_ (Sigma-Aldrich, catalogue number: S6014), 12.7 mmol L^−1^ KCl (Sigma-Aldrich, catalogue number: P3911), 0.57 mmol L^−1^ MgCl_2_ (Sigma-Aldrich, catalogue number: M8266), 5.55 mmol L^−1^,D-glucose (Sigma-Aldrich, catalogue number: G8270), 63.5 mmol L^−1^ sucrose (Sigma-Aldrich, catalogue number: S8501), 16.5 mmol L^−1^ Tris (2-Amino-2-hydroxymethyl-propane-1,3-diol) (Sigma-Aldrich, catalogue number: T1503), 100 mmol L^−1^ NaCl (Sigma-Aldrich, catalogue number: S9888), 0.7 mmol L^−1^ Na2SO4 (Sigma-Aldrich, catalogue number: 238597) in water at pH 7.6.^75,76^ MVs collected from different three batches (two of batches from male mice and one from female mouse) were then incubated in SCL buffer containing 2.0 mmolL^−1^ CaCl_2_ (Sigma-Aldrich, catalogue number: C1016), and 2.0 mmolL^−1^ adenosinetriphosphate (ATP) (Sigma-Aldrich, catalogue number: A6419) in 96 wells plates for 30 hr at 37◦C. The concentration of added MVs was either 1x10e9 or 1x10e7 per ml and the final volume adjusted to 200 μL of each well. Besides the addition of MVs, either sphingomyelin synthase 2 inhibitor (SMS2-IN-2) (final concentration: 500 nM; Sigma-Aldrich, catalogue number: T7263) or positive control Levamisole (final concentration: 1.0 mM; Sigma-Aldrich, catalogue number: L-025) were added and characterized as MVs + SMS2-IN-2 group or MVs + Levamisole group, respectively. The final volume of each well was adjusted to 200 μL and the turbidity was measured at 340 nm, with 15-min intervals in duplicates (Plate Reader TECAN infinite Mplex, Tecan Trading AG, Switzerland). The changes in the values of turbidity from baseline vs time (hr) and iAUC of different groups were calculated and plotted using GraphPad Prism.

**Collection of mouse tissues for histological analyses**

Tissues were collected at twenty-eight days of age from wild-type mice on a mixed background. Briefly, hindlimbs were dissected so that the tibia, femur and knee joint were intact, with the skin removed and excess muscle partially trimmed. Samples were fixed in pre-cooled 4 % formaldehyde/PBS for 48 hours at 4°C, then subsequently placed into 30 % sucrose overnight, prior to embedding within OCT medium in cryomolds (62534-1; Tissue-Tek). Samples were stored at -80 °C before processing. In the experiment where LRCs were labeled with EdU (A10044; Invitrogen), mice were given 65 µg EdU/g body mass by daily intraperitoneal injections on post-natal days 6-9.

**RNAscope *in situ* hybridization**

Cryo-sections at 10 μm thickness were prepared from blocks containing 48-hour fixed samples using Leica CM3050 S Cryostat (LeicaBiosystems), placed onto SuperFrost Ultra Plus GOLD Adhesion slides (11976299; Thermo Fisher Scientific), and stored at -80°C before processing.

To visualize RNA molecules on tissue section we utilized RNAscope® as previously described,^79^ outlined in the supplementary methods section. The RNA expression levels for our target were investigated using RNAscope® Multiplex Fluorescent Detection Reagents v2 kit (323100; ACD) and RNAscope® 4-Plex Ancillary kit (323120; ACD) using probes for poly(A) (423731; ACD), hh-CHRDL2 (1043521-C1; ACD), hh-THBS3 (1274141-C2; ACD), hh-CLIC3 (569631-C3; ACD), and hh-SFRP5 (1056891-C4; ACD). Positive RNA control probe (320881; ACD) and negative RNA control probe (320871; ACD) were used to validate the RNAscope assay on mouse or human sections.

**RNAscope *in situ* hybridization with EdU detection**

For standard RNAscope *in situ* hybridization, slides were removed from -80 °C freezer and allowed to equilibrate at room temperature until condensation had dried. The samples were incubated in 1x Target Retrieval Solution (322000; ACD) inside a Certoclav CV II/1600 pressure cooker containing 500 ml distilled water, and heated until the pressure reached 0.2 Bar. Slides were then washed in nuclease-free water (NFW [10977-035; Invitrogen]) for 15 seconds, absolute ethanol for 3 minutes, and air-dried. After washing with copious amounts of NFW, sections were covered with Protease III (322337; ACD) and incubated at 40 °C for 30 minutes. The sections were subsequently washed twice in NFW for 2 minutes, incubated with RNAscope Hydrogen Peroxide (322337; ACD) for 10 minutes, and then washed twice by incubating with copious amounts of NFW for 2 minutes. Next, probe hybridization was performed by adding 2-3 drops (~20 µl per drop) of target probes, diluted as per the manufacturer´s instructions, and incubated for 2 hours at 40 °C.

After hybridization, amplification was performed: RNAscope Multiplex v2 Amp1 was applied and incubated for 30 minutes at 40 °C, then RNAscope Multiplex v2 Amp2 was applied and incubated for 30 minutes at 40 °C and finally RNAscope Multiplex v2 Amp3 applied and incubated for 15 minutes at 40 °C. Between all steps, slides were washed twice in 1X Wash Buffer for 2 minutes.

Depending on the number of probes used and their respective channels, the following steps were performed multiple times in sequential order (i.e. from C1-C4) using different fluorophores each time; on the final occasion, DAPI was added to the HRP blocker. RNAscope Multiplex v2 HRP-C1-4 (corresponding to the probes used) were applied and incubated for 15 minutes at 40°C; staining was performed using diluted TSA Plus fluorophores diluted at 1:750 as per the manufacturer´s instructions for 30 minutes at 40°C; HRP blocker (323107; ACD) was applied and incubated for 15 minutes at 40°C. Between all steps, slides were washed twice in 1X Wash Buffer for 2 minutes. After the final wash step, slides were rinsed once in PBS prior to mounting in Fluoroshield (F6182; Sigma-Aldrich) and were stored at 4°C.

When RNAscope was combined with EdU detection, we followed the RNAscope *in situ* hybridization protocol described above, with the following deviations. First, DAPI was not included in the final HRP blocking step, and after the final wash step (mentioned in the paragraph above), we processed the slides as follows: sections were blocked using 3 % normal horse serum in PBS/T (PBS containing 0.05 % Tween20) for 1 hour at room temperature. Sections were washed with PBS/T (3 times for 5 minutes) and exposed to reaction buffer of distilled water containing 100 mM Tris (from 1M stock, pH 7.5), 2 mM CuSO_4_, 1 μM Alexa-azide 555 (from a 1 mM stock in DMSO), and 100 mM ascorbic acid (added immediately prior to use from a 0.5 M stock in distilled water). Slides were protected from light and incubated at room temperature for 30 minutes, and gently agitated twice during this time. Subsequently, slides were washed once in PBS/T then counterstained with DAPI (1.5 µg/ml in PBS). Slides were then rinsed with PBS (3 times for 5 minutes) and mounted with Fluoroshield.

**Image capture and analysis specific to supplementary figures**

For the quantification of cell number per spot (Fig. S1b), for each zone the number of cells were divided by the area of zone, measured in square micrometre, and multiplied by the area of spots of spatial transcriptomics platform. The values were taken into RStudio to analyze and ggplot package was used to plot.

For the quantification of mean poly(A) signal intensity per nucleus as a percentage of maximum value in EdU-labeled cells in mouse growth plate (Fig. S3c), the microscopy MIP files were loaded into CellProfiler and following steps were used: multichannel image was split and converted to grayscale, each nucleus was identified as an object by selecting DAPI channel, EdU-labeled nucleus was identified as an object by selecting EdU channel, poly(A) signal was measured in nuclei of EdU positive cells and EdU negative cells. The same settings for object size and method were used for every sample. The values for condition were saved as separate csv files and taken into RStudio to analyze. Signal intensity was normalized within the growth plate of each mouse and ggplot package was used to plot.

**Extended legends for main figures**

**Figure 1. Resting zone chondrocytes contain relatively low amounts of mRNA.**

(**a**) The procedure for collecting human growth plate biopsies during epiphysiodesis surgeries. (**b**) Hematoxylin and eosin (H&E)-stained tissue section (b´) was histologically separated into areas of growth plate zones and SOC, and spots representing UMI counts from RRST profiling were overlaid (b´´). (**c**) Level-plot of UMI counts per section in patients 1 to 4 for RRST and 5 to 8 for CytAssist. (**d**) Violin plot depicting the Ln transformed average of spot UMI counts in each zone per section in both RRST and CytAssist. The comparison of means between zones in RRST was analyzed using one-way ANOVA (p-value < 0.01) and Tukey post-hoc test: **p < 0.01 and *p < 0.05 from 8 sections from 4 patients. The comparison of means between zones in CytAssist was analyzed using one-way ANOVA (p-value < 0.01) and Tukey post-hoc test: **p < 0.01 from 9 sections from 4 patients (two sections from patient 5 on panel C. shows lack of HZ). (**e**) Representative image of RNAscope assay targeting poly(A) expression in different zones of the human growth plate. (**f**) Mean signal intensity per nucleus was quantified in each zone and converted to a percentage value using the maximum value for each patient. To compare the average signal intensity medians between zones, we used Kruskal-Wallis (p-value < 0.0001) and Wilcoxon post-hoc test: ****p < 0.0001 for all three comparisons. Nuclei from n = 2084 cells in RZ, n = 1985 cells in PZ, n = 784 cells in HZ from 5 patients.

**Figure 2. Resting zone chondrocytes display features of cellular quiescence *in vivo*.** (**a**) Relative nuclear abundance of poly(A) mRNA was analyzed using Kruskal-Wallis (p-value < 0.0001) and Wilcoxon post-hoc test. The y-axis is plotted on a logarithmic scale with base 10. The median of each zone (RZ = 6.8104, PZ = 5.9712, HZ = 5.7397) is displayed (n = 2860 cells in RZ, n = 2557 cells in PZ, n = 860 cells in HZ from 5 patients). (**b**) TEM images of heterochromatin (asterisks) and lysosomes (arrowheads) in RZ and PZ chondrocytes. (**c**) The percentage of each nucleus comprising heterochromatin, and (**d**) the number of lysosomes per cell were analyzed using Mann-Whitney test (44 - 62 cells per zone, from 3 patients). (**e**) High magnification images (from Fig. S3d,e) indicating zone-specific thymidine-analogue labelling of chondrocytes after *ex vivo* culture. (**f**) Analysis of EdU, IdU and CldU incorporation was conducted using pair-wise matching sample ANOVA (EdU p-value < 0.05, IdU p-value = 0.0989, CldU p-value < 0.001) and Tukey post-hoc test (n = 646 cells in RZ, n = 1227 cells in PZ, n = 505 cells in HZ from 3 patients). (**g**) Poly(A) RNA visualized after culture was compared with fresh-frozen slices (presented in Fig. 1F) and analyzed using Mann-Whitney tests. The median of each zone is presented: uncultured RZ = 26.2469, cultured RZ = 46.5278, uncultured PZ = 62.7071, cultured PZ = 69.2647, uncultured HZ = 81.2138, cultured HZ = 69.5188 (for cultured biopsies; n = 1194 cells in RZ, n = 1454 cells in PZ, n = 1053 cells in HZ from 3 patients). In all panels, *p < 0.05, **p < 0.01, ***p < 0.001, ****p < 0.0001.

**Figure 3. Spatial profiles of cartilage and SOC markers visualized on human growth plate sections.** (**a**) Pseudo-Bulk analysis workflow used to analyze SRT data. SRT data collected from RRST and CytAssist platforms was used for transcriptomics analysis. (1) samples were manually annotated based on histology into the different areas: RZ, PZ, HZ, and SOC. Reads per gene from each section were summed per region creating a pseudo-bulk count matrix. Normalization and log transformation of the counts was performed with DESeq2. (2) Principal Component Analysis (PCA) was performed for each platform, where samples grouped distinctly by area. (3) Pair-wise differential expression analysis was performed between the different areas in each platform. Each area-specific marker was significantly up-regulated using both platforms. (**b**) Expression heatmap of 12 selected markers of cartilage and bone in the epiphyseal areas, and (**c**) Spatial feature plot of selected markers of bone and cartilage.

**Figure 4. SRT reveals 138 differentially expressed genes between the three growth plate zones and SOC.** (**a**) Expression heatmap of 138 genes significantly up-regulated between both platforms in one of the areas above all others. Z-scores for each gene are plotted, blue indicating lower expression and red higher expression of the gene in each sample. Upper bars indicate the sample area labelled, and left bars indicate the area associated for each gene, with RZ in red, PZ in yellow, HZ in green and SOC in blue. (**b**) Spatial feature plot of examples of significantly up-regulated genes for growth plate cartilage and SOC.

**Figure 5. Visium HD spatial representation of known and novel zone-specific markers.** (**a**) High resolution microphotographs of histological section stained with hematoxylin and eosin (H&E) (i), and a simplified version (ii) overlaid with dots representing ACAN expression from Visium HD in individual cells segmented with Bin2cell using bins of 2 µm resolution. Each square indicates a region of the sample used to visualize the expression of the different markers at higher magnification. (iii) *ACAN* expression in region 1 shown in the H&E image (left) and in the simplified version (right). (**b-m**) Enlarged views of marker expression in H&E image (left) and in the simplified version (right). RZ markers, *SFRP5*, *CLU*, *CHRDL2* and *UCMA* (b-e), PZ markers, *COL2A1*, *COL9A1*, *THBS3*, and *CNMD* (f-i), and HZ markers *IHH*, *COL10A1,* *CLIC3* and *LOXL4* (**j-m**). For each gene, the corresponding region is indicated in the upper left corner.

**Figure 6. SGMS2 localizes to growth plate-derived matrix vesicles (MVs) and contributes to mineralization.** (**a**) Spatial expression of SGMS2 across growth plate zones (RZ, PZ, HZ, SOC) determined by Visium and Visium HD confirming pseudo-bulk analysis. SGMS2 is enriched in HZ. For Visium HD, each dot represents an individual cell as segmented by Bin2cell. (**b**) Workflow for isolation of MVs from murine growth plate cartilage. (**c**) Heatmap presenting log-transformed protein abundance for all male/female samples and the total for each protein showing enrichment of canonical EV markers (CD63, CD81, TSG101, ALIX) and MV markers (ANXA5, ALPL, PHOSPHO1, ENPP1), alongside detection of SGMS2 at levels comparable to other MV markers, indicated using arrows. (**d**) The molecular functions of proteins enriched in MVs were identified using GO terms by comparing them with the entire proteome. (**e**) Mineralization assay using isolated MVs at two concentrations (E7 and E9) in the presence or absence of SGMS2 inhibitor (SMS2-IN-2) or alkaline phosphatase inhibitor (levamisole), showing changes in turbidity over time when MVs at each time point the mean is indicated and error bars represent standard deviation. (**f**) Mineralization kinetics by turbidity (absorbance at 340 nm) at the 26 hr timepoint (from panel e) were quantified.

**Figure. 7. CHRDL2 and SFRP5 can be used to label distinct resting zone sub-populations.** (**a**) UMI counts per spot were quantified in the sub-populations of the significantly up-regulated genes in RZ. Medians were statistically compared using Kruskal-Wallis (p-value < 0.0001) and Wilcoxon post-hoc test: *p < 0.05, **p < 0.01, ***p < 0.001, ****p < 0.0001. (**b**) The abundance of the sub-populations in all 8 patients was visualized. (**c**) Spatial feature plot of spot for single expression of CHRDL2 (in red) and SFRP5 (in green) and their co-expression (in orange). Patient 2 is female and patient 3 is male. (**d**) High-magnification images allow visualization of SFRP5/CHRDL2-double-positive cells (arrow d´), -single-positive (arrowheads, d´, d´´) or -double-negative (arrows in d´´ and d´´´). (**e**) Poly(A) signal intensity was quantified in the sub-populations. Medians were statistically compared using Kruskal-Wallis (p-value < 0.001) and Wilcoxon post-hoc test: ****p < 0.0001. The median of each group being CHRDL2+ = 0.1422, SFRP5+ = 0.1200, CHRDL2+ SFRP5+ = 0.1763, Double negative = 0.0210 (n = 381 cells in RZ from 3 patients).

**Supplementary notes**

Supplementary note #1 – Discussion regarding anticipated genes identified within human growth plates by SRT.

Within the RZ, half of the up-regulated genes have been previously detected in corresponding cells in rodents (*Ucma*,^80^ *Sfrp5*^81^). The two other genes, *CHRDL2* and *ZNF550*, are newly identified RZ markers, although *Chrdl2* is expressed in RZ cells in mice prior to, but not after SOC formation.^82^ As a BMP pathway antagonist, *CHRDL2* expression in the human RZ fits well with our understanding of BMP signaling in rodents in which BMP antagonists, such as *Bmp3* and *Grem1*, ^83^ are expressed in the RZ. These results agree with the overall understanding of BMP signaling in growth plate and shed light on a previously unreported species-specific regulator of human growth.

SRT analysis also enabled us to identify a large number of uniquely expressed genes in the other growth plate zones, which closely aligned with their anticipated function. For example, several of the genes enriched in the PZ are involved in cartilage formation (e.g. *SOX5*,^84^ *COL16A1*,^85^ *CHPF*^86^), a particularly active process in this region, as our ORA analysis confirmed. *Fmod* ^87^ *Cnmd* ^88^ and *Thbs3* ^89^ are enriched in PZ chondrocytes in developing rodents. Of the genes enriched in HZ chondrocytes, many have been previously reportedly expressed in these cells: *ALPL*,^90^ *CDKN1C*,^91^ *COL10A1*,^92^ *FN1* (as well as RZ cells, prior to SOC formation in mice),^93^ *MGP* (previously reported in PZ and HZ of neonatal mice).^94^

**References within Supplementary Materials**

58.	National Institutes of Health. Download ImageJ. https://imagej.nih.gov/ij/download.html (2023).

59.	Mirzazadeh, R. *et al.* Spatially resolved transcriptomic profiling of degraded and challenging fresh frozen samples. *Nat. Commun.* 14, 509 (2023).

60.	Zhang, Y., Parmigiani, G. & Johnson, W. E. ComBat-seq: batch effect adjustment for RNA-seq count data. *NAR Genom. Bioinform.* 2, lqaa078 (2020).

61. Michael Love *et al.* Differential gene expression analysis based on the

negative binomial distribution. *Bioconductor 3.19*.

62.	Guillemette Marot, Andrea Rau, Florence Jaffrezic & Samuel Blanck. metaRNASeq: Meta-Analysis of RNA-Seq Data. *The Comprehensive R Archive Network* (2021).

63.	Schneider, C. A., Rasband, W. S. & Eliceiri, K. W. NIH Image to ImageJ: 25 years of image analysis. Nat. Methods 9, 671–675 (2012).

64.	Rabineau, M. et al. Cell guidance into quiescent state through chromatin remodeling induced by elastic modulus of substrate. Biomaterials 37, 144–155 (2015).

65.	Barral, D. C. et al. Current methods to analyze lysosome morphology, positioning, motility and function. Traffic vol. 23 238–269 Preprint at https://doi.org/10.1111/tra.12839 (2022).

66.	Newton, P. T. et al. A radical switch in clonality reveals a stem cell niche in the epiphyseal growth plate. Nature 567, 234–238 (2019).

67.	Polański, K. et al. Bin2cell reconstructs cells from high resolution Visium HD data. Bioinformatics 40, btae546 (2024).

68.	Görgens, A. *et al.* Identification of storage conditions stabilizing extracellular vesicles preparations. *J. Extracell. Vesicles* 11, e12238 (2022).

69. Marchan‐Alvarez, J. G. et al. A protocol to differentiate the chondrogenic ATDC5 cell‐line for the collection of chondrocyte‐derived extracellular vesicles. Journal of Extracellular Biology 3, e70004 (2024).

70.	Zhang, Y., Lan, M. & Chen, Y. Minimal Information for Studies of Extracellular Vesicles (MISEV): Ten-Year Evolution (2014–2023). *Pharmaceutics* 16, 1394 (2024).

71.	Van Deun, J. *et al.* EV-TRACK: transparent reporting and centralizing knowledge in extracellular vesicle research. *Nat. Methods* 14, 228–232 (2017).

72. Kim, S. & Pevzner, P. A. MS-GF+ makes progress towards a universal

database search tool for proteomics. *Nat. Commun.* 5, 5277 (2014).

73.	Granholm, V. *et al.* Fast and Accurate Database Searches with MS-GF+Percolator. *J. Proteome Res.* 13, 890–897 (2014).

74.	Savitski, M. M., Wilhelm, M., Hahne, H., Kuster, B. & Bantscheff, M. A Scalable Approach for Protein False Discovery Rate Estimation in Large

75. Proteomic Data Sets. *Molecular & Cellular Proteomics* 14, 2394–2404 (2015).

75.	Hindy Yuen Shing Yan, Vallelado Mario Villaizan & Sep905. Missforest in python - arguably the best missing values imputation method. https://github.com/yuenshingyan/MissForest/tree/v1.0.0 (2024).

76.	Eden, E., Navon, R., Steinfeld, I., Lipson, D. & Yakhini, Z. GOrilla: a tool for discovery and visualization of enriched GO terms in ranked gene lists. *BMC Bioinformatics* 10, 48 (2009).

77.	Eden, E., Lipson, D., Yogev, S. & Yakhini, Z. Discovering Motifs in Ranked Lists of DNA Sequences. *PLoS Comput. Biol.* 3, e39 (2007).

78. Wu, LiciaN. Y. *et al.* Physicochemical Characterization of the Nucleational

Core of Matrix Vesicles. *Journal of Biological Chemistry* 272, 4404–4411

(1997).

79.	Cominal, J. G. *et al.* A protein corona modulates the function of mineralization-competent matrix vesicles. *JBMR Plus* 9, ziae168 (2025).

80.	Tagariello, A. *et al.* Ucma - A novel secreted factor represents a highly specific marker for distal chondrocytes. *Matrix Biology* 27, 3–11 (2008).

81.	Lui, J. C. K. *et al.* Spatial and temporal regulation of gene expression in the mammalian growth plate. *Bone* 46, 1380-90 (2010).

82. Nakayama, N. *et al.* A novel chordin-like BMP inhibitor, CHL2, expressed

preferentially in chondrocytes of developing cartilage and osteoarthritic joint

cartilage. *Development* 131, 229-40 (2004).

83. Garrison, P., Yue, S., Hanson, J., Baron, J. & Lui, J. C. Spatial regulation of bone morphogenetic proteins (BMPs) in postnatal articular and growth plate cartilage. *PLoS One* 12, e0176752 (2017).

84.	Smits, P. *et al.* The Transcription Factors L-Sox5 and Sox6 Are Essential for Cartilage Formation. *Dev. Cell* 1, 277–290 (2001).

85.	Kassner, A. *et al.* Discrete integration of collagen XVI into tissue-specific collagen fibrils or beaded microfibrils. *Matrix Biology* 22, 131-43 (2003).

86.	Ogawa, H. *et al.* Chondroitin Sulfate Synthase-2 Is Necessary for Chain Extension of Chondroitin Sulfate but Not Critical for Skeletal Development. *PLoS One* 7, e43806 (2012).

87.	Itzstein, C. *et al.* Fibromodulin is expressed by both chondrocytes and osteoblasts during fetal bone development. *J. Cell. Biochem.* 82, 46-57 (2001).

88.	Amil, B. *et al.* Chondromodulin-I expression in the growth plate of young uremic rats. *Kidney Int.* 66, 51–59 (2004).

89. Iruela‐Arispe, M. L., Liska, D. A. J., Sage, E. H. & Bornstein, P. Differential

expression of thrombospondin 1, 2, and 3 during murine development.

*Developmental Dynamics* 197, 40-56 (1993).

90. Miao, D. & Scutt, A. Histochemical localization of alkaline phosphatase activity

in decalcified bone and cartilage. *Journal of Histochemistry and*

*Cytochemistry* 50, 333-40 (2002).

91. Yan, Y., Frisén, J., Lee, M. H., Massagué, J. & Barbacid, M. Ablation of the

CDK inhibitor p57(Kip2) results in increased apoptosis and delayed

differentiation during mouse development. *Genes Dev.* 11, 973-83 (1997).

92.	Chen, J. *et al.* Hypertrophic chondrocyte-specific Col10a1 controlling	elements in Cre recombinase transgenic studies. *Am. J. Transl. Res.* 11,

6672-6679 (2019).

93. Dinesh, N. E. H. *et al.* Role of Fibronectin in Postnatal Skeletal Development.

*bioRxiv* 2024.02.01.578475 (2024) doi:10.1101/2024.02.01.578475.

94.	Luo, G. *et al.* Spontaneous calcification of arteries and cartilage in mice lacking

matrix GLA protein. *Nature* 386, 78–81 (1997).

**Supplementary Figures**

**Figure S1. Detailed assessment of SRT application to human growth plate biopsies.** (**a**) High magnification image indicating bone marrow of the primary spongiosa with hematoxylin and eosin (left) and UMI count overlaid (right). (**b**) Number of cells per 55 µm^2^ (the diameter of one Visium spot) was quantified in each zone of human growth plate using DAPI staining of the sections used in panel 1F. The comparison of the number of cells between zones was conducted using Kruskal-Wallis (p-value = 0.1677), the median for each zone is RZ = 0.7834, PZ = 1.7458, HZ = 0.8681 from 5 patients.

**Figure S2. mRNA in mouse resting zone chondrocytes has predominantly nuclear localization.** (**a,b)** RNAscope staining of poly(A) tail of proximal tibial growth plate of mouse at P28. (**c**) Quantification of mean poly(A) signal intensity per nucleus in each growth plate zone. The comparison of medians between zones was conducted using Kruskal-Wallis (p-value < 0.0001) and Wilcoxon post-hoc test: ****p < 0.0001. The median of each zone being RZ = 69.6850, PZ = 92.2472, HZ = 67.9876 (n = 1891 cells in RZ, n = 4197 cells in PZ, n = 2714 cells in HZ). (**d**) RZ chondrocytes of mouse growth plate zones have significantly higher relative poly(A) signal intensity in the nucleus. Data was pooled from 3 mice and analyzed using Kruskal-Wallis (p-value < 0.0001) and Wilcoxon post-hoc test: ****p < 0.0001, ***p < 0.001. The median of each zone was RZ = 4.8801, PZ = 3.7429, HZ = 4.5268 (n = 2657 cells in RZ, n = 6600 cells in PZ, n = 3081 cells in HZ).

**Figure S3. Low mRNA levels are a strong indicator of LRCs in mice.** (**a**) Workflow for labeling EdU label-retaining RZ chondrocytes in mice at P28. (**b**) Proximal tibial growth plates were stained for EdU and poly(A) tails of RNA using RNAscope. (**c**) Quantification of mean poly(A) signal intensity per nucleus was performed in EdU positive and EdU negative cells. Data was pooled from 4 mice and analyzed using Mann-Whitney-U test: p-value < 0.05. The median of each condition was EdU-negative = 27.70, EdU-positive = 18.49 (n = 6910 for EdU negative cells and n = 45 for EdU positive cells).(**d**) Workflow illustrates the *ex vivo* culture of human growth plate biopsies with thymidine analogues, and (**e**) representative images following visualization. (**f**) Poly(A) RNA visualized after culture.

**Figure S4. SRT validation using an additional dataset and *in situ* hybridization.** (**a**) Expression heatmap of 130 genes significantly upregulated in one of the areas above all others in SRT analysis (Fig. 4), within the validation dataset. Genes labeled with an asterisk were significantly upregulated in the validation dataset. Z-scores for each gene are plotted, blue indicating lower expression and red higher expression of the gene in each sample. The upper bars indicate the sample area, and left bars indicate the area associated with each gene. RZ in red, PZ in yellow, HZ in green and SOC in blue. (**b**) Human growth plate was stained using RNAscope to visualize markers of the RZ (CHRDL2), PZ (THBS3) and HZ (CLIC3) identified by SRT; n = 1 patient.

**Figure S5. Over-Representation Analysis on zone-specific markers.** (**a-d)** show GO biological process over-representation analysis for marker genes from RZ, PZ, HZ, and SOC, respectively.

**Figure S6. Zone-specific enrichment of clinically relevant genes in the human growth plate.** (a, b) NKX3-2 expression is enriched in the PZ, supported by spatial overlays using Visium and Visium HD. (c, d) WNK4 is enriched in hypertrophic zone HZ chondrocytes. For Visium HD, each dot represents an individual cell as segmented by Bin2cell.

**Figure S7. Characterization of matrix vesicles (MVs) isolated from murine growth plate cartilage.** (a) Schematic of the MV isolation protocol from growth plate cartilage. (b) Cell viability of cartilage samples post-isolation procedure. (c) Nanoparticle tracking analysis (NTA) of isolated particles. (d–f) Quantitative comparison of particle size (mode), total EV number, and particle-to-protein ratio between male and female samples. (g) Transmission electron microscopy (TEM) images of isolated particles. (h) Western blot analysis of MV preparations. (i) Alkaline phosphatase (ALP) activity assay.

**Figure S8. Identification and spatial validation of RZ-specific markers in the murine growth plate.** (a) Heatmap showing 31 genes significantly upregulated in the RZ compared to PZ and HZ, excluding statistical comparisons with the secondary ossification center SOC. (b–e) Spatial transcriptomic validation of selected RZ markers using Visium and Visium HD platforms. Expression profiles of ZNF550 on Visium (b) and Visium HD (c) and COL15A1 on Visium (d) and Visium HD (e) show enrichment in the RZ. (f) Pathway over-representation analysis (ORA) of RZ-upregulated genes reveals significant involvement of WNT and BMP signaling pathways. (g–h) Spatial expression profiles of previously described RZ marker APOE on Visium (g) and Visium HD (h) show higher expression in RZ but were not significantly enriched in this dataset. For Visium HD, each dot represents an individual cell as segmented by Bin2cell.

**Figure S9. Quantification of sub-populations of human RZ chondrocytes.** (**a**) Pie charts visualizing the relative abundance of spots containing the various sub-populations of RZ markers identified from SRT from the entire population (left) and among sub-populations (right). (**b**) Power analysis was performed to determine the number of patients needed to be included to have a power of 0.8 for genetic sex and Tanner stage, based on both platforms. (**c**) Pie chart visualizing the relative abundance of spots containing sub-populations of RZ markers identified by their expression of CHRDL2 or SFRP5 using RNAscope.

**Table S1. SRT Validation dataset.** Metadata of the patient information regarding the tissues analyzed in Fig. S4a. Patient IDs numbering correspond to a continuation from Table 1. Duplication in this table indicates samples from the same patient were run in different capture areas.

| **Patient ID** | **Platform** | **Genetic sex** | **Tanner pubertal stage PH** | **Tanner pubertal stage B** | **Tanner pubertal stage G** | **Patient age at surgery** |
| --- | --- | --- | --- | --- | --- | --- |
| 1 | RRST | M | 3 | - | 3 | 13 y 2 m |
| 1 | RRST | M | 3 | - | 3 | 13 y 2 m |
| 4 | RRST | F | 3 to 4 | 4 | - | 12 y 11 m |
| 5 | RRST | M | 2 | - | 2 | 12 y 2 m |
| 6 | RRST | F | 4 | 4 | - | 14 y 6 m |
| 6 | RRST | F | 4 | 4 | - | 14 y 6 m |
| 9 | RRST | M | 4 | - | 4 | 13 y 10 m |
| 10 | RRST | M | 4 | - | 5 | 14 y 4 m |

**Table S2. Protein abundance in matrix vesicles isolated from growth plates of male and female mice.**

| **Proteins** | **Male** | | | **Female** | | | **Total** |
| --- | --- | --- | --- | --- | --- | --- | --- |
|  | **1** | **2** | **3** | **1** | **2** | **3** |  |
| Krt5 | 1,87E+00 | 2,00E+00 | 1,90E+00 | 1,76E+00 | 1,71E+00 | 1,88E+00 | 2,64E+00 |
| Dsp | 1,83E+00 | 1,97E+00 | 1,88E+00 | 1,80E+00 | 1,68E+00 | 1,83E+00 | 2,62E+00 |
| Mfge8 | 1,82E+00 | 1,91E+00 | 1,81E+00 | 1,87E+00 | 1,32E+00 | 1,78E+00 | 2,56E+00 |
| Krt14 | 1,79E+00 | 1,83E+00 | 1,78E+00 | 1,75E+00 | 1,60E+00 | 1,78E+00 | 2,54E+00 |
| Actc1 | 1,72E+00 | 1,63E+00 | 1,84E+00 | 1,72E+00 | 1,72E+00 | 1,70E+00 | 2,50E+00 |
| Krt6a | 1,72E+00 | 1,83E+00 | 1,71E+00 | 1,51E+00 | 1,70E+00 | 1,74E+00 | 2,49E+00 |
| Acta1 | 1,58E+00 | 1,67E+00 | 1,87E+00 | 1,74E+00 | 1,26E+00 | 1,73E+00 | 2,46E+00 |
| Krt16 | 1,65E+00 | 1,79E+00 | 1,63E+00 | 1,65E+00 | 1,51E+00 | 1,72E+00 | 2,45E+00 |
| Krt17 | 1,66E+00 | 1,73E+00 | 1,75E+00 | 1,59E+00 | 1,51E+00 | 1,70E+00 | 2,44E+00 |
| Krt42 | 1,65E+00 | 1,74E+00 | 1,69E+00 | 1,63E+00 | 1,57E+00 | 1,67E+00 | 2,44E+00 |
| Krt10 | 1,60E+00 | 1,77E+00 | 1,67E+00 | 1,70E+00 | 1,38E+00 | 1,65E+00 | 2,42E+00 |
| Ahnak | 1,79E+00 | 1,67E+00 | 1,68E+00 | 1,86E+00 | 9,03E-01 | 1,38E+00 | 2,42E+00 |
| Atp2a1 | 1,57E+00 | 1,62E+00 | 1,82E+00 | 1,66E+00 | 1,30E+00 | 1,70E+00 | 2,42E+00 |
| Hapln1 | 1,75E+00 | 1,59E+00 | 1,68E+00 | 1,60E+00 | 1,28E+00 | 1,60E+00 | 2,38E+00 |
| Actg1 | 1,51E+00 | 1,57E+00 | 1,80E+00 | 1,64E+00 | 1,20E+00 | 1,65E+00 | 2,37E+00 |
| Krt2 | 1,53E+00 | 1,68E+00 | 1,58E+00 | 1,52E+00 | 1,41E+00 | 1,61E+00 | 2,34E+00 |
| Anxa6 | 1,58E+00 | 1,53E+00 | 1,64E+00 | 1,61E+00 | 1,34E+00 | 1,49E+00 | 2,32E+00 |
| Krt1 | 1,51E+00 | 1,65E+00 | 1,51E+00 | 1,38E+00 | 1,38E+00 | 1,46E+00 | 2,27E+00 |
| Krt75 | 1,48E+00 | 1,64E+00 | 1,52E+00 | 1,30E+00 | 1,48E+00 | 1,46E+00 | 2,27E+00 |
| Atp1a1 | 1,46E+00 | 1,49E+00 | 1,56E+00 | 1,53E+00 | 1,34E+00 | 1,45E+00 | 2,26E+00 |
| Krt77 | 1,45E+00 | 1,57E+00 | 1,45E+00 | 1,40E+00 | 1,23E+00 | 1,48E+00 | 2,22E+00 |
| Jup | 1,49E+00 | 1,56E+00 | 1,46E+00 | 1,36E+00 | 1,18E+00 | 1,41E+00 | 2,20E+00 |
| Myh4 | 1,42E+00 | 1,36E+00 | 1,54E+00 | 1,41E+00 | 1,40E+00 | 1,32E+00 | 2,19E+00 |
| Acan | 1,45E+00 | 1,48E+00 | 1,56E+00 | 1,43E+00 | 1,15E+00 | 1,32E+00 | 2,19E+00 |
| Anxa5 | 1,34E+00 | 1,43E+00 | 1,45E+00 | 1,41E+00 | 1,20E+00 | 1,40E+00 | 2,16E+00 |
| Anxa2 | 1,40E+00 | 1,45E+00 | 1,36E+00 | 1,43E+00 | 1,15E+00 | 1,40E+00 | 2,15E+00 |
| Lrp1 | 1,49E+00 | 1,48E+00 | 1,34E+00 | 1,46E+00 | 6,02E-01 | 1,28E+00 | 2,13E+00 |
| Krt79 | 1,32E+00 | 1,41E+00 | 1,38E+00 | 1,28E+00 | 1,26E+00 | 1,38E+00 | 2,12E+00 |
| Srl | 1,11E+00 | 1,48E+00 | 1,41E+00 | 1,53E+00 | 4,77E-01 | 1,34E+00 | 2,11E+00 |
| Myh1 | 1,20E+00 | 1,34E+00 | 1,41E+00 | 1,41E+00 | 1,30E+00 | 1,26E+00 | 2,11E+00 |
| Edil3 | 1,32E+00 | 1,36E+00 | 1,36E+00 | 1,36E+00 | 1,00E+00 | 1,36E+00 | 2,09E+00 |
| Gm5478 | 1,23E+00 | 1,38E+00 | 1,32E+00 | 1,30E+00 | 1,28E+00 | 1,26E+00 | 2,08E+00 |
| Myh9 | 1,48E+00 | 1,46E+00 | 1,23E+00 | 1,36E+00 | 3,01E-01 | 1,18E+00 | 2,06E+00 |
| Atp1a2 | 1,28E+00 | 1,23E+00 | 1,38E+00 | 1,32E+00 | 1,27E+00 | 1,20E+00 | 2,06E+00 |
| Krt19 | 1,28E+00 | 1,28E+00 | 1,32E+00 | 1,34E+00 | 1,15E+00 | 1,28E+00 | 2,06E+00 |
| Msn | 1,28E+00 | 1,26E+00 | 1,28E+00 | 1,43E+00 | 8,45E-01 | 1,32E+00 | 2,05E+00 |
| Eno1b | 1,32E+00 | 1,38E+00 | 1,36E+00 | 1,26E+00 | 1,00E+00 | 1,18E+00 | 2,05E+00 |
| Hspa5 | 1,28E+00 | 1,38E+00 | 1,28E+00 | 1,28E+00 | 1,11E+00 | 1,20E+00 | 2,04E+00 |
| Meltf | 1,28E+00 | 1,30E+00 | 1,30E+00 | 1,32E+00 | 1,00E+00 | 1,20E+00 | 2,03E+00 |
| Hspa8 | 1,28E+00 | 1,34E+00 | 1,34E+00 | 1,23E+00 | 9,54E-01 | 1,23E+00 | 2,03E+00 |
| Pdcd6ip | 1,32E+00 | 1,38E+00 | 1,28E+00 | 1,34E+00 | 6,02E-01 | 1,20E+00 | 2,03E+00 |
| Alpl | 1,20E+00 | 1,34E+00 | 1,34E+00 | 1,26E+00 | 9,54E-01 | 1,20E+00 | 2,01E+00 |
| Col6a3 | 1,40E+00 | 1,36E+00 | 1,46E+00 | 7,78E-01 | 6,99E-01 | 1,18E+00 | 2,01E+00 |
| Krt76 | 1,18E+00 | 1,34E+00 | 1,23E+00 | 1,18E+00 | 1,15E+00 | 1,30E+00 | 2,01E+00 |
| Atp2b1 | 1,30E+00 | 1,23E+00 | 1,38E+00 | 1,34E+00 | 6,99E-01 | 1,18E+00 | 2,01E+00 |
| Anxa4 | 1,32E+00 | 1,23E+00 | 1,30E+00 | 1,30E+00 | 1,04E+00 | 1,08E+00 | 2,00E+00 |
| Ckap4 | 1,26E+00 | 1,30E+00 | 1,30E+00 | 1,26E+00 | 9,03E-01 | 1,15E+00 | 1,99E+00 |
| P4hb | 1,23E+00 | 1,23E+00 | 1,28E+00 | 1,15E+00 | 1,04E+00 | 1,18E+00 | 1,97E+00 |
| Krt13 | 4,34E-07 | 4,34E-07 | 4,34E-07 | 1,48E+00 | 1,36E+00 | 1,54E+00 | 1,96E+00 |
| Krt72 | 1,11E+00 | 1,36E+00 | 1,17E+00 | 1,00E+00 | 1,11E+00 | 1,18E+00 | 1,95E+00 |
| Igf2r | 1,34E+00 | 1,15E+00 | 1,15E+00 | 1,28E+00 | 4,77E-01 | 1,18E+00 | 1,94E+00 |
| Hspg2 | 1,20E+00 | 1,26E+00 | 1,11E+00 | 1,18E+00 | 1,16E+00 | 1,00E+00 | 1,94E+00 |
| Pkm | 1,11E+00 | 1,20E+00 | 1,26E+00 | 1,23E+00 | 1,00E+00 | 1,08E+00 | 1,93E+00 |
| Atp2a2 | 1,28E+00 | 1,41E+00 | 1,57E+00 | 4,34E-07 | 4,34E-07 | 4,34E-07 | 1,93E+00 |
| Pdia3 | 1,20E+00 | 1,18E+00 | 1,23E+00 | 1,18E+00 | 7,78E-01 | 1,15E+00 | 1,92E+00 |
| Eef2 | 1,18E+00 | 1,18E+00 | 1,20E+00 | 1,20E+00 | 6,02E-01 | 1,15E+00 | 1,90E+00 |
| Anxa11 | 1,20E+00 | 1,11E+00 | 1,23E+00 | 1,18E+00 | 9,54E-01 | 9,54E-01 | 1,90E+00 |
| Golim4 | 1,26E+00 | 1,23E+00 | 1,18E+00 | 1,20E+00 | 3,01E-01 | 1,00E+00 | 1,89E+00 |
| Rdx | 1,20E+00 | 1,20E+00 | 1,00E+00 | 1,28E+00 | 4,77E-01 | 1,11E+00 | 1,89E+00 |
| Myof | 1,36E+00 | 1,15E+00 | 1,11E+00 | 1,36E+00 | 4,34E-07 | 3,01E-01 | 1,88E+00 |
| Tfrc | 1,23E+00 | 1,23E+00 | 1,15E+00 | 1,11E+00 | 6,02E-01 | 1,00E+00 | 1,88E+00 |
| Hsp90b1 | 1,23E+00 | 1,15E+00 | 1,11E+00 | 1,15E+00 | 6,02E-01 | 1,08E+00 | 1,87E+00 |
| Slc8a3 | 1,08E+00 | 1,32E+00 | 1,11E+00 | 1,08E+00 | 6,02E-01 | 1,04E+00 | 1,86E+00 |
| Pgk1 | 1,20E+00 | 1,18E+00 | 1,20E+00 | 1,04E+00 | 6,99E-01 | 1,00E+00 | 1,86E+00 |
| Rpn1 | 1,11E+00 | 1,08E+00 | 1,18E+00 | 1,11E+00 | 8,45E-01 | 1,08E+00 | 1,86E+00 |
| Ldha | 1,18E+00 | 1,15E+00 | 1,04E+00 | 1,15E+00 | 7,78E-01 | 1,04E+00 | 1,85E+00 |
| Nucb1 | 1,11E+00 | 1,11E+00 | 1,04E+00 | 1,11E+00 | 9,03E-01 | 1,11E+00 | 1,85E+00 |
| Canx | 1,15E+00 | 1,15E+00 | 1,08E+00 | 1,15E+00 | 7,78E-01 | 1,00E+00 | 1,85E+00 |
| Ywhae | 1,20E+00 | 1,11E+00 | 1,04E+00 | 1,18E+00 | 6,02E-01 | 1,00E+00 | 1,84E+00 |
| Marcks | 1,11E+00 | 1,11E+00 | 1,23E+00 | 1,04E+00 | 6,99E-01 | 1,00E+00 | 1,84E+00 |
| Calr | 1,04E+00 | 1,15E+00 | 1,08E+00 | 1,15E+00 | 6,02E-01 | 1,11E+00 | 1,83E+00 |
| Anxa1 | 1,04E+00 | 1,04E+00 | 1,11E+00 | 1,20E+00 | 9,03E-01 | 9,03E-01 | 1,83E+00 |
| Hsp90ab1 | 1,18E+00 | 1,04E+00 | 9,54E-01 | 1,11E+00 | 1,01E+00 | 9,03E-01 | 1,82E+00 |
| Ttn | 1,04E+00 | 1,04E+00 | 1,30E+00 | 4,34E-07 | 1,05E+00 | 1,06E+00 | 1,82E+00 |
| Col6a1 | 1,18E+00 | 1,08E+00 | 1,11E+00 | 1,06E+00 | 6,02E-01 | 1,00E+00 | 1,82E+00 |
| Papss2 | 1,11E+00 | 1,11E+00 | 1,08E+00 | 1,18E+00 | 4,34E-07 | 1,04E+00 | 1,81E+00 |
| Capn6 | 1,08E+00 | 1,04E+00 | 1,11E+00 | 1,11E+00 | 4,77E-01 | 1,08E+00 | 1,81E+00 |
| Gsn | 1,11E+00 | 1,08E+00 | 9,03E-01 | 1,15E+00 | 6,02E-01 | 1,08E+00 | 1,80E+00 |
| Col6a2 | 1,18E+00 | 1,08E+00 | 1,11E+00 | 1,04E+00 | 3,01E-01 | 1,00E+00 | 1,80E+00 |
| Ptk7 | 1,00E+00 | 1,15E+00 | 9,54E-01 | 1,08E+00 | 9,84E-01 | 9,03E-01 | 1,80E+00 |
| Scin | 1,04E+00 | 1,11E+00 | 9,54E-01 | 1,04E+00 | 1,02E+00 | 9,03E-01 | 1,80E+00 |
| Ppa1 | 1,08E+00 | 1,11E+00 | 1,15E+00 | 9,54E-01 | 6,99E-01 | 9,54E-01 | 1,79E+00 |
| Slc3a2 | 1,00E+00 | 1,00E+00 | 1,04E+00 | 1,15E+00 | 6,02E-01 | 1,11E+00 | 1,79E+00 |
| Mvp | 1,15E+00 | 1,00E+00 | 1,00E+00 | 1,00E+00 | 9,66E-01 | 8,45E-01 | 1,78E+00 |
| Pdia4 | 1,11E+00 | 9,54E-01 | 9,03E-01 | 1,20E+00 | 9,48E-01 | 6,99E-01 | 1,78E+00 |
| Tuba1c | 1,04E+00 | 1,00E+00 | 1,11E+00 | 1,04E+00 | 4,77E-01 | 1,04E+00 | 1,77E+00 |
| Gapdh | 1,00E+00 | 1,00E+00 | 1,08E+00 | 1,04E+00 | 4,77E-01 | 1,11E+00 | 1,77E+00 |
| Plcd1 | 1,08E+00 | 1,08E+00 | 1,00E+00 | 1,20E+00 | 4,34E-07 | 9,03E-01 | 1,77E+00 |
| Serpinh1 | 1,04E+00 | 9,54E-01 | 1,15E+00 | 1,00E+00 | 6,02E-01 | 9,54E-01 | 1,76E+00 |
| Epb41l2 | 1,11E+00 | 1,11E+00 | 9,03E-01 | 1,18E+00 | 4,34E-07 | 8,45E-01 | 1,76E+00 |
| Eef1a1 | 1,00E+00 | 1,00E+00 | 1,04E+00 | 9,54E-01 | 8,45E-01 | 9,54E-01 | 1,75E+00 |
| Smpd3 | 1,04E+00 | 1,08E+00 | 1,00E+00 | 1,04E+00 | 6,99E-01 | 8,45E-01 | 1,75E+00 |
| Gdi2 | 9,54E-01 | 1,04E+00 | 1,00E+00 | 9,54E-01 | 9,03E-01 | 9,03E-01 | 1,74E+00 |
| Krt78 | 9,03E-01 | 1,15E+00 | 1,00E+00 | 8,45E-01 | 8,45E-01 | 9,54E-01 | 1,74E+00 |
| Gnb2 | 9,54E-01 | 1,00E+00 | 9,57E-01 | 9,03E-01 | 9,55E-01 | 9,54E-01 | 1,73E+00 |
| Lman1 | 1,04E+00 | 1,04E+00 | 1,04E+00 | 9,03E-01 | 4,77E-01 | 1,00E+00 | 1,73E+00 |
| Atp2b4 | 9,54E-01 | 9,54E-01 | 1,00E+00 | 9,54E-01 | 9,48E-01 | 9,03E-01 | 1,73E+00 |
| Aldoa | 1,00E+00 | 1,00E+00 | 1,08E+00 | 1,04E+00 | 3,01E-01 | 9,03E-01 | 1,72E+00 |
| Ptgfrn | 1,08E+00 | 1,04E+00 | 1,00E+00 | 1,11E+00 | 4,34E-07 | 7,78E-01 | 1,72E+00 |
| Cspg4 | 1,11E+00 | 1,08E+00 | 9,54E-01 | 1,04E+00 | 4,34E-07 | 8,45E-01 | 1,72E+00 |
| Slc38a10 | 1,08E+00 | 1,04E+00 | 1,04E+00 | 8,45E-01 | 8,85E-01 | 6,02E-01 | 1,72E+00 |
| Cacna2d1 | 9,54E-01 | 9,03E-01 | 1,00E+00 | 1,00E+00 | 9,23E-01 | 8,45E-01 | 1,72E+00 |
| Gnas | 9,54E-01 | 1,00E+00 | 8,45E-01 | 1,08E+00 | 6,99E-01 | 9,54E-01 | 1,72E+00 |
| H4c11 | 9,54E-01 | 8,45E-01 | 1,04E+00 | 1,04E+00 | 6,02E-01 | 1,00E+00 | 1,72E+00 |
| Vim | 1,08E+00 | 8,45E-01 | 9,03E-01 | 9,54E-01 | 9,00E-01 | 8,45E-01 | 1,71E+00 |
| Ezr | 9,03E-01 | 9,03E-01 | 9,11E-01 | 1,04E+00 | 8,98E-01 | 8,45E-01 | 1,70E+00 |
| Itgav | 1,04E+00 | 7,78E-01 | 9,54E-01 | 1,00E+00 | 8,70E-01 | 6,99E-01 | 1,68E+00 |
| Ywhab | 1,00E+00 | 9,03E-01 | 1,00E+00 | 1,00E+00 | 4,34E-07 | 9,54E-01 | 1,68E+00 |
| Anxa3 | 1,08E+00 | 9,03E-01 | 7,78E-01 | 9,54E-01 | 8,67E-01 | 6,99E-01 | 1,68E+00 |
| Hsp90aa1 | 1,00E+00 | 8,45E-01 | 9,03E-01 | 1,04E+00 | 8,61E-01 | 6,02E-01 | 1,67E+00 |
| Rab1a | 9,03E-01 | 9,54E-01 | 1,04E+00 | 1,08E+00 | 3,01E-01 | 6,99E-01 | 1,67E+00 |
| Tm9sf4 | 9,54E-01 | 1,00E+00 | 1,00E+00 | 9,54E-01 | 4,34E-07 | 9,03E-01 | 1,67E+00 |
| Prdx1 | 1,00E+00 | 8,45E-01 | 9,54E-01 | 1,04E+00 | 6,02E-01 | 7,78E-01 | 1,67E+00 |
| Actn4 | 8,45E-01 | 9,54E-01 | 6,99E-01 | 1,00E+00 | 8,87E-01 | 8,90E-01 | 1,67E+00 |
| Gnaq | 7,78E-01 | 1,08E+00 | 9,03E-01 | 9,54E-01 | 3,01E-01 | 9,54E-01 | 1,66E+00 |
| Gna11 | 8,45E-01 | 9,03E-01 | 9,54E-01 | 9,54E-01 | 6,02E-01 | 9,54E-01 | 1,66E+00 |
| Lmna | 1,08E+00 | 1,15E+00 | 7,78E-01 | 8,45E-01 | 4,77E-01 | 6,02E-01 | 1,66E+00 |
| Basp1 | 9,03E-01 | 9,03E-01 | 9,03E-01 | 8,45E-01 | 8,76E-01 | 8,45E-01 | 1,66E+00 |
| Slc44a2 | 8,45E-01 | 9,03E-01 | 9,54E-01 | 9,03E-01 | 8,64E-01 | 7,78E-01 | 1,66E+00 |
| H2bc4 | 9,03E-01 | 8,45E-01 | 9,54E-01 | 9,54E-01 | 7,78E-01 | 7,78E-01 | 1,65E+00 |
| Alb | 1,04E+00 | 6,99E-01 | 8,45E-01 | 1,00E+00 | 6,02E-01 | 9,03E-01 | 1,65E+00 |
| Cpm | 9,03E-01 | 9,54E-01 | 6,99E-01 | 1,04E+00 | 8,50E-01 | 6,02E-01 | 1,64E+00 |
| Tln1 | 9,03E-01 | 7,78E-01 | 6,02E-01 | 1,08E+00 | 8,58E-01 | 8,32E-01 | 1,64E+00 |
| Hal | 9,03E-01 | 1,04E+00 | 8,45E-01 | 9,03E-01 | 4,34E-07 | 9,54E-01 | 1,64E+00 |
| Ppia | 8,45E-01 | 9,54E-01 | 1,04E+00 | 8,45E-01 | 6,02E-01 | 7,78E-01 | 1,64E+00 |
| Anxa8 | 9,54E-01 | 9,54E-01 | 9,54E-01 | 9,03E-01 | 4,34E-07 | 9,03E-01 | 1,64E+00 |
| Glg1 | 1,00E+00 | 8,45E-01 | 9,03E-01 | 1,00E+00 | 3,01E-01 | 8,45E-01 | 1,64E+00 |
| Pkp1 | 9,03E-01 | 1,04E+00 | 7,78E-01 | 8,45E-01 | 4,77E-01 | 9,03E-01 | 1,63E+00 |
| Bsg | 1,00E+00 | 9,54E-01 | 8,45E-01 | 6,99E-01 | 6,02E-01 | 8,45E-01 | 1,62E+00 |
| Tpi1 | 9,54E-01 | 1,00E+00 | 6,99E-01 | 1,00E+00 | 4,34E-07 | 8,45E-01 | 1,62E+00 |
| Pgam1 | 9,03E-01 | 1,00E+00 | 7,78E-01 | 9,54E-01 | 3,01E-01 | 8,45E-01 | 1,62E+00 |
| Ehd1 | 7,78E-01 | 9,03E-01 | 8,45E-01 | 9,03E-01 | 8,31E-01 | 7,78E-01 | 1,62E+00 |
| Tubb2a | 8,45E-01 | 7,78E-01 | 7,78E-01 | 9,54E-01 | 8,28E-01 | 8,30E-01 | 1,62E+00 |
| Anxa7 | 8,45E-01 | 9,54E-01 | 8,45E-01 | 9,54E-01 | 6,99E-01 | 6,02E-01 | 1,61E+00 |
| Dsg1a | 8,45E-01 | 8,45E-01 | 8,45E-01 | 8,45E-01 | 6,99E-01 | 9,03E-01 | 1,61E+00 |
| Ppib | 8,45E-01 | 7,78E-01 | 1,00E+00 | 9,54E-01 | 4,77E-01 | 7,78E-01 | 1,61E+00 |
| Calu | 8,45E-01 | 8,45E-01 | 7,78E-01 | 8,45E-01 | 8,36E-01 | 8,45E-01 | 1,61E+00 |
| Rcn1 | 7,78E-01 | 7,78E-01 | 8,45E-01 | 9,03E-01 | 8,29E-01 | 8,32E-01 | 1,61E+00 |
| Nedd4 | 9,03E-01 | 9,54E-01 | 7,78E-01 | 6,99E-01 | 8,12E-01 | 7,78E-01 | 1,61E+00 |
| Ptgis | 1,00E+00 | 9,54E-01 | 6,02E-01 | 9,54E-01 | 8,05E-01 | 3,01E-01 | 1,61E+00 |
| Tnc | 9,54E-01 | 8,45E-01 | 9,03E-01 | 7,78E-01 | 7,91E-01 | 6,02E-01 | 1,60E+00 |
| Myh10 | 8,45E-01 | 7,78E-01 | 6,99E-01 | 9,54E-01 | 8,14E-01 | 8,12E-01 | 1,60E+00 |
| Anpep | 1,00E+00 | 9,03E-01 | 6,99E-01 | 1,08E+00 | 4,34E-07 | 6,02E-01 | 1,60E+00 |
| Gnai2 | 7,78E-01 | 7,78E-01 | 9,03E-01 | 7,78E-01 | 6,99E-01 | 9,54E-01 | 1,60E+00 |
| Nt5e | 8,45E-01 | 1,04E+00 | 8,45E-01 | 8,45E-01 | 3,01E-01 | 7,78E-01 | 1,60E+00 |
| Nme1nme2 | 6,99E-01 | 9,03E-01 | 8,45E-01 | 9,03E-01 | 6,99E-01 | 8,45E-01 | 1,60E+00 |
| Iqgap1 | 9,03E-01 | 9,03E-01 | 4,77E-01 | 1,04E+00 | 8,17E-01 | 4,77E-01 | 1,60E+00 |
| Ehd2 | 9,03E-01 | 9,03E-01 | 6,02E-01 | 1,00E+00 | 7,87E-01 | 4,77E-01 | 1,59E+00 |
| Hspa1l | 7,78E-01 | 9,03E-01 | 8,02E-01 | 7,78E-01 | 8,04E-01 | 8,07E-01 | 1,59E+00 |
| Slc16a1 | 6,99E-01 | 9,03E-01 | 8,45E-01 | 9,54E-01 | 3,01E-01 | 9,03E-01 | 1,59E+00 |
| Prkg2 | 9,03E-01 | 1,08E+00 | 6,99E-01 | 7,78E-01 | 7,76E-01 | 3,01E-01 | 1,59E+00 |
| Plxnb2 | 8,45E-01 | 9,03E-01 | 6,02E-01 | 8,45E-01 | 8,16E-01 | 8,00E-01 | 1,59E+00 |
| Gna13 | 6,99E-01 | 8,45E-01 | 7,78E-01 | 9,03E-01 | 8,05E-01 | 8,10E-01 | 1,59E+00 |
| Myo1d | 9,03E-01 | 8,45E-01 | 4,77E-01 | 9,03E-01 | 8,20E-01 | 7,84E-01 | 1,59E+00 |
| Calml3 | 8,45E-01 | 1,00E+00 | 6,02E-01 | 7,78E-01 | 7,85E-01 | 6,99E-01 | 1,58E+00 |
| Stt3a | 9,54E-01 | 9,03E-01 | 8,45E-01 | 7,78E-01 | 4,77E-01 | 6,99E-01 | 1,58E+00 |
| Nucb2 | 9,54E-01 | 8,45E-01 | 8,45E-01 | 9,54E-01 | 4,34E-07 | 6,99E-01 | 1,58E+00 |
| Rab10 | 8,45E-01 | 9,54E-01 | 8,45E-01 | 7,78E-01 | 7,55E-01 | 4,77E-01 | 1,58E+00 |
| Cltc | 1,04E+00 | 6,99E-01 | 6,99E-01 | 6,99E-01 | 7,53E-01 | 7,67E-01 | 1,57E+00 |
| Slc25a4 | 6,99E-01 | 6,99E-01 | 7,78E-01 | 9,03E-01 | 8,05E-01 | 8,45E-01 | 1,57E+00 |
| Gnai3 | 7,78E-01 | 6,99E-01 | 8,45E-01 | 6,99E-01 | 8,04E-01 | 9,03E-01 | 1,57E+00 |
| Atp5f1b | 7,78E-01 | 9,03E-01 | 8,45E-01 | 9,54E-01 | 4,34E-07 | 7,78E-01 | 1,57E+00 |
| Slc16a3 | 7,78E-01 | 8,45E-01 | 7,78E-01 | 8,45E-01 | 7,67E-01 | 6,99E-01 | 1,57E+00 |
| Rpl6 | 1,00E+00 | 7,78E-01 | 6,99E-01 | 8,45E-01 | 7,37E-01 | 4,77E-01 | 1,56E+00 |
| Rpl7 | 8,45E-01 | 8,45E-01 | 6,02E-01 | 8,45E-01 | 7,93E-01 | 6,99E-01 | 1,56E+00 |
| Phospho1 | 8,45E-01 | 8,45E-01 | 8,45E-01 | 7,78E-01 | 4,34E-07 | 9,03E-01 | 1,56E+00 |
| Ywhaz | 8,45E-01 | 8,45E-01 | 7,78E-01 | 8,45E-01 | 3,01E-01 | 8,45E-01 | 1,56E+00 |
| Rab7 | 9,03E-01 | 8,45E-01 | 7,78E-01 | 9,03E-01 | 4,34E-07 | 7,78E-01 | 1,56E+00 |
| Farp1 | 7,78E-01 | 8,45E-01 | 6,99E-01 | 7,78E-01 | 7,75E-01 | 7,71E-01 | 1,55E+00 |
| Apoe | 7,78E-01 | 9,54E-01 | 6,99E-01 | 7,78E-01 | 7,54E-01 | 6,02E-01 | 1,55E+00 |
| Cpne8 | 7,78E-01 | 9,03E-01 | 6,99E-01 | 8,45E-01 | 7,50E-01 | 6,02E-01 | 1,55E+00 |
| Krt23 | 6,02E-01 | 7,78E-01 | 9,03E-01 | 7,46E-01 | 7,66E-01 | 7,78E-01 | 1,55E+00 |
| Tubb4b | 8,45E-01 | 7,78E-01 | 6,02E-01 | 1,00E+00 | 7,31E-01 | 4,77E-01 | 1,55E+00 |
| Myl1 | 4,77E-01 | 6,99E-01 | 9,03E-01 | 8,45E-01 | 7,79E-01 | 7,78E-01 | 1,54E+00 |
| Rpn2 | 8,45E-01 | 7,78E-01 | 9,03E-01 | 9,03E-01 | 4,34E-07 | 6,99E-01 | 1,54E+00 |
| Cgref1 | 7,78E-01 | 9,03E-01 | 7,78E-01 | 6,99E-01 | 6,02E-01 | 7,78E-01 | 1,54E+00 |
| Trpv4 | 8,45E-01 | 7,78E-01 | 7,78E-01 | 9,03E-01 | 4,77E-01 | 6,99E-01 | 1,54E+00 |
| Myl12a | 8,45E-01 | 7,78E-01 | 8,45E-01 | 6,99E-01 | 7,39E-01 | 6,02E-01 | 1,54E+00 |
| Ywhah | 7,78E-01 | 7,78E-01 | 6,02E-01 | 8,45E-01 | 7,55E-01 | 7,61E-01 | 1,54E+00 |
| Stk26 | 8,45E-01 | 6,99E-01 | 6,99E-01 | 9,03E-01 | 7,02E-01 | 6,02E-01 | 1,53E+00 |
| Atp1b3 | 8,45E-01 | 9,03E-01 | 7,78E-01 | 8,45E-01 | 3,01E-01 | 6,02E-01 | 1,53E+00 |
| Tmed10 | 8,45E-01 | 9,03E-01 | 7,78E-01 | 7,78E-01 | 4,34E-07 | 7,78E-01 | 1,53E+00 |
| Rpl4 | 7,78E-01 | 6,99E-01 | 6,02E-01 | 9,03E-01 | 7,27E-01 | 6,99E-01 | 1,52E+00 |
| Pebp1 | 6,99E-01 | 7,78E-01 | 8,45E-01 | 8,45E-01 | 3,01E-01 | 7,78E-01 | 1,52E+00 |
| Tubb5 | 8,45E-01 | 7,78E-01 | 7,78E-01 | 9,54E-01 | 4,34E-07 | 6,02E-01 | 1,52E+00 |
| Atp5f1a | 8,45E-01 | 6,02E-01 | 9,03E-01 | 9,54E-01 | 4,34E-07 | 6,02E-01 | 1,52E+00 |
| Vcl | 9,03E-01 | 9,03E-01 | 6,99E-01 | 6,99E-01 | 6,84E-01 | 3,01E-01 | 1,52E+00 |
| Slc25a5 | 6,99E-01 | 6,99E-01 | 6,99E-01 | 8,45E-01 | 7,16E-01 | 6,99E-01 | 1,51E+00 |
| Ryr1 | 3,01E-01 | 6,99E-01 | 9,54E-01 | 8,45E-01 | 7,12E-01 | 6,02E-01 | 1,51E+00 |
| Gm8797 | 9,54E-01 | 4,77E-01 | 8,45E-01 | 7,78E-01 | 4,77E-01 | 6,02E-01 | 1,51E+00 |
| Smpdl3b | 7,78E-01 | 8,45E-01 | 8,45E-01 | 7,78E-01 | 3,01E-01 | 6,02E-01 | 1,51E+00 |
| Stxbp1 | 4,77E-01 | 6,99E-01 | 7,78E-01 | 8,45E-01 | 7,41E-01 | 7,28E-01 | 1,50E+00 |
| Gfpt1 | 6,99E-01 | 8,45E-01 | 4,77E-01 | 7,78E-01 | 7,32E-01 | 7,05E-01 | 1,50E+00 |
| Rpl15 | 6,99E-01 | 7,78E-01 | 7,78E-01 | 6,02E-01 | 7,10E-01 | 6,99E-01 | 1,49E+00 |
| Arf3 | 6,99E-01 | 7,78E-01 | 7,78E-01 | 7,78E-01 | 7,06E-01 | 4,77E-01 | 1,49E+00 |
| Sri | 7,78E-01 | 8,45E-01 | 6,02E-01 | 7,78E-01 | 7,00E-01 | 4,77E-01 | 1,49E+00 |
| Tkt | 9,03E-01 | 9,54E-01 | 4,77E-01 | 6,99E-01 | 4,34E-07 | 6,99E-01 | 1,49E+00 |
| Cyb5r3 | 7,78E-01 | 7,78E-01 | 7,78E-01 | 7,78E-01 | 3,01E-01 | 6,99E-01 | 1,49E+00 |
| Rasa3 | 6,99E-01 | 7,78E-01 | 4,77E-01 | 8,45E-01 | 6,96E-01 | 6,84E-01 | 1,49E+00 |
| Tmed2 | 8,45E-01 | 7,78E-01 | 6,99E-01 | 6,02E-01 | 6,75E-01 | 6,02E-01 | 1,49E+00 |
| Fap | 9,54E-01 | 6,99E-01 | 4,77E-01 | 7,78E-01 | 6,31E-01 | 4,77E-01 | 1,48E+00 |
| Tpp2 | 1,00E+00 | 6,02E-01 | 6,99E-01 | 7,78E-01 | 6,11E-01 | 4,34E-07 | 1,48E+00 |
| Gys1 | 6,99E-01 | 7,78E-01 | 6,02E-01 | 6,99E-01 | 7,04E-01 | 6,99E-01 | 1,48E+00 |
| Napa | 6,02E-01 | 6,99E-01 | 7,78E-01 | 6,99E-01 | 7,01E-01 | 6,99E-01 | 1,48E+00 |
| Golm2 | 8,45E-01 | 8,45E-01 | 6,99E-01 | 7,78E-01 | 3,01E-01 | 4,77E-01 | 1,48E+00 |
| Sgms2 | 6,99E-01 | 8,45E-01 | 6,99E-01 | 6,99E-01 | 4,34E-07 | 8,45E-01 | 1,48E+00 |
| Fscn1 | 8,45E-01 | 7,78E-01 | 7,78E-01 | 7,78E-01 | 4,34E-07 | 6,02E-01 | 1,48E+00 |
| Rpl18 | 6,99E-01 | 6,99E-01 | 6,99E-01 | 6,99E-01 | 6,99E-01 | 6,99E-01 | 1,48E+00 |
| Myo1c | 6,99E-01 | 7,78E-01 | 4,77E-01 | 8,45E-01 | 6,75E-01 | 6,02E-01 | 1,47E+00 |
| Cpne1 | 8,45E-01 | 7,78E-01 | 6,99E-01 | 6,99E-01 | 6,65E-01 | 3,01E-01 | 1,47E+00 |
| Ace | 8,45E-01 | 6,02E-01 | 4,77E-01 | 7,78E-01 | 6,95E-01 | 6,67E-01 | 1,47E+00 |
| Col1a1 | 9,54E-01 | 7,78E-01 | 6,02E-01 | 6,99E-01 | 6,24E-01 | 4,34E-07 | 1,47E+00 |
| Mrc1 | 6,99E-01 | 8,45E-01 | 8,45E-01 | 9,03E-01 | 4,34E-07 | 4,34E-07 | 1,46E+00 |
| Idh1 | 7,78E-01 | 8,45E-01 | 6,99E-01 | 7,78E-01 | 3,01E-01 | 4,77E-01 | 1,46E+00 |
| Casq1 | 4,77E-01 | 6,99E-01 | 8,45E-01 | 7,78E-01 | 4,77E-01 | 6,99E-01 | 1,46E+00 |
| Blmh | 6,99E-01 | 8,45E-01 | 6,02E-01 | 7,78E-01 | 4,77E-01 | 6,02E-01 | 1,46E+00 |
| Itgb1 | 6,99E-01 | 7,78E-01 | 8,45E-01 | 6,99E-01 | 4,34E-07 | 6,99E-01 | 1,46E+00 |
| Arhgdia | 6,99E-01 | 7,78E-01 | 7,78E-01 | 6,99E-01 | 4,77E-01 | 6,02E-01 | 1,46E+00 |
| Tm9sf3 | 6,99E-01 | 6,99E-01 | 8,45E-01 | 6,99E-01 | 3,01E-01 | 6,99E-01 | 1,46E+00 |
| Ywhaq | 7,78E-01 | 7,78E-01 | 6,02E-01 | 8,45E-01 | 4,34E-07 | 6,99E-01 | 1,46E+00 |
| Slc25a3 | 6,99E-01 | 6,99E-01 | 6,02E-01 | 6,99E-01 | 6,85E-01 | 6,99E-01 | 1,46E+00 |
| Trf | 7,78E-01 | 6,99E-01 | 4,77E-01 | 7,78E-01 | 6,75E-01 | 6,02E-01 | 1,46E+00 |
| Rps9 | 8,45E-01 | 7,78E-01 | 4,77E-01 | 7,78E-01 | 6,56E-01 | 3,01E-01 | 1,46E+00 |
| Rab8a | 8,45E-01 | 6,02E-01 | 6,99E-01 | 6,99E-01 | 6,41E-01 | 4,77E-01 | 1,45E+00 |
| Prdx6 | 6,02E-01 | 8,45E-01 | 6,99E-01 | 6,99E-01 | 6,41E-01 | 4,77E-01 | 1,45E+00 |
| Galnt2 | 7,78E-01 | 6,99E-01 | 6,02E-01 | 8,45E-01 | 6,34E-01 | 3,01E-01 | 1,45E+00 |
| Ephx1 | 6,02E-01 | 6,99E-01 | 6,02E-01 | 7,78E-01 | 6,64E-01 | 6,68E-01 | 1,45E+00 |
| Enpp1 | 6,99E-01 | 8,45E-01 | 6,02E-01 | 7,78E-01 | 4,77E-01 | 4,77E-01 | 1,45E+00 |
| Aoc3 | 9,03E-01 | 3,01E-01 | 6,02E-01 | 9,54E-01 | 3,01E-01 | 4,77E-01 | 1,45E+00 |
| Cat | 8,45E-01 | 9,03E-01 | 6,99E-01 | 6,02E-01 | 4,34E-07 | 4,77E-01 | 1,45E+00 |
| Rras2 | 7,78E-01 | 6,02E-01 | 8,45E-01 | 6,99E-01 | 3,01E-01 | 6,02E-01 | 1,45E+00 |
| Rpl7a | 6,99E-01 | 6,99E-01 | 6,02E-01 | 7,78E-01 | 6,52E-01 | 4,77E-01 | 1,44E+00 |
| Trdn | 3,01E-01 | 6,02E-01 | 9,03E-01 | 6,99E-01 | 6,41E-01 | 6,02E-01 | 1,44E+00 |
| P4ha1 | 7,78E-01 | 7,78E-01 | 6,99E-01 | 6,99E-01 | 6,32E-01 | 4,34E-07 | 1,44E+00 |
| Eif4a1 | 6,02E-01 | 7,78E-01 | 6,02E-01 | 7,78E-01 | 6,27E-01 | 4,77E-01 | 1,44E+00 |
| Niban2 | 7,78E-01 | 6,02E-01 | 6,99E-01 | 7,78E-01 | 6,16E-01 | 3,01E-01 | 1,43E+00 |
| Kras | 6,99E-01 | 6,02E-01 | 8,45E-01 | 7,78E-01 | 4,34E-07 | 6,02E-01 | 1,43E+00 |
| Lman2 | 8,45E-01 | 7,78E-01 | 6,99E-01 | 6,02E-01 | 4,34E-07 | 6,02E-01 | 1,43E+00 |
| Scara3 | 7,78E-01 | 6,02E-01 | 4,34E-07 | 8,45E-01 | 6,61E-01 | 6,29E-01 | 1,43E+00 |
| Ganab | 6,99E-01 | 7,78E-01 | 4,77E-01 | 6,02E-01 | 6,44E-01 | 6,41E-01 | 1,43E+00 |
| Hras | 6,02E-01 | 6,99E-01 | 6,36E-01 | 7,78E-01 | 6,18E-01 | 4,77E-01 | 1,42E+00 |
| Akr1a1 | 6,99E-01 | 6,02E-01 | 4,77E-01 | 8,45E-01 | 6,17E-01 | 4,77E-01 | 1,42E+00 |
| Ppp3ca | 6,02E-01 | 6,02E-01 | 6,02E-01 | 7,78E-01 | 6,16E-01 | 6,02E-01 | 1,42E+00 |
| Cpne3 | 6,99E-01 | 6,99E-01 | 6,99E-01 | 6,99E-01 | 6,11E-01 | 3,01E-01 | 1,42E+00 |
| Slc12a2 | 6,99E-01 | 6,02E-01 | 7,78E-01 | 7,78E-01 | 6,10E-01 | 4,34E-07 | 1,42E+00 |
| Cd63 | 6,02E-01 | 6,99E-01 | 6,99E-01 | 7,78E-01 | 4,34E-07 | 6,99E-01 | 1,41E+00 |
| Ywhag | 7,78E-01 | 6,99E-01 | 6,99E-01 | 6,99E-01 | 4,34E-07 | 6,02E-01 | 1,41E+00 |
| Snap23 | 7,78E-01 | 6,99E-01 | 7,78E-01 | 6,02E-01 | 4,34E-07 | 6,02E-01 | 1,41E+00 |
| Myl6 | 6,99E-01 | 7,78E-01 | 6,99E-01 | 6,99E-01 | 4,34E-07 | 6,02E-01 | 1,41E+00 |
| Rhoa | 8,45E-01 | 6,02E-01 | 6,99E-01 | 6,02E-01 | 3,01E-01 | 6,02E-01 | 1,41E+00 |
| Slc1a5 | 6,02E-01 | 6,02E-01 | 8,45E-01 | 6,99E-01 | 3,01E-01 | 6,02E-01 | 1,41E+00 |
| Slc7a5 | 6,02E-01 | 6,02E-01 | 6,99E-01 | 6,02E-01 | 6,14E-01 | 6,02E-01 | 1,40E+00 |
| Lin7c | 6,02E-01 | 6,02E-01 | 6,99E-01 | 6,99E-01 | 6,11E-01 | 4,77E-01 | 1,40E+00 |
| H13 | 6,99E-01 | 6,99E-01 | 6,99E-01 | 4,77E-01 | 6,08E-01 | 4,77E-01 | 1,40E+00 |
| Vcan | 6,99E-01 | 6,02E-01 | 6,02E-01 | 6,99E-01 | 3,01E-01 | 6,99E-01 | 1,40E+00 |
| Calm3 | 6,02E-01 | 6,02E-01 | 8,45E-01 | 6,02E-01 | 4,34E-07 | 6,99E-01 | 1,40E+00 |
| Ugdh | 7,78E-01 | 7,78E-01 | 4,77E-01 | 7,78E-01 | 4,34E-07 | 4,77E-01 | 1,40E+00 |
| Sec61a1 | 7,78E-01 | 7,78E-01 | 6,02E-01 | 6,02E-01 | 3,01E-01 | 4,77E-01 | 1,40E+00 |
| Itga5 | 6,02E-01 | 6,99E-01 | 6,02E-01 | 7,78E-01 | 5,80E-01 | 3,01E-01 | 1,39E+00 |
| Gpi1 | 6,99E-01 | 6,02E-01 | 6,02E-01 | 8,45E-01 | 4,34E-07 | 5,59E-01 | 1,39E+00 |
| Lnpep | 7,78E-01 | 6,99E-01 | 6,02E-01 | 6,99E-01 | 5,54E-01 | 4,34E-07 | 1,39E+00 |
| Itga1 | 6,99E-01 | 4,77E-01 | 6,99E-01 | 4,77E-01 | 6,01E-01 | 6,10E-01 | 1,38E+00 |
| Cav1 | 6,02E-01 | 4,77E-01 | 6,02E-01 | 6,99E-01 | 6,04E-01 | 6,02E-01 | 1,38E+00 |
| Gdi1 | 6,99E-01 | 6,99E-01 | 3,01E-01 | 6,02E-01 | 6,21E-01 | 5,83E-01 | 1,38E+00 |
| Steap3 | 7,78E-01 | 6,99E-01 | 4,77E-01 | 8,45E-01 | 4,34E-07 | 3,01E-01 | 1,38E+00 |
| Rab2a | 6,99E-01 | 7,78E-01 | 4,77E-01 | 7,78E-01 | 4,34E-07 | 4,77E-01 | 1,38E+00 |
| Rap1a | 6,02E-01 | 6,02E-01 | 6,99E-01 | 6,01E-01 | 5,87E-01 | 4,77E-01 | 1,38E+00 |
| Slc12a4 | 6,02E-01 | 6,99E-01 | 4,77E-01 | 6,99E-01 | 5,83E-01 | 4,77E-01 | 1,38E+00 |
| Cd81 | 6,99E-01 | 6,02E-01 | 4,77E-01 | 6,99E-01 | 5,68E-01 | 4,77E-01 | 1,37E+00 |
| Cab39 | 7,78E-01 | 6,02E-01 | 6,02E-01 | 6,02E-01 | 5,67E-01 | 3,01E-01 | 1,37E+00 |
| Stxbp3 | 8,45E-01 | 6,99E-01 | 3,01E-01 | 6,02E-01 | 5,66E-01 | 3,01E-01 | 1,37E+00 |
| Park7 | 6,99E-01 | 6,02E-01 | 7,78E-01 | 4,77E-01 | 5,65E-01 | 3,01E-01 | 1,37E+00 |
| Uqcrc2 | 6,02E-01 | 6,02E-01 | 6,99E-01 | 6,99E-01 | 5,52E-01 | 3,01E-01 | 1,37E+00 |
| Bpnt2 | 4,77E-01 | 4,77E-01 | 6,99E-01 | 6,99E-01 | 3,01E-01 | 6,99E-01 | 1,36E+00 |
| Pacsin2 | 6,02E-01 | 6,99E-01 | 7,78E-01 | 6,99E-01 | 4,34E-07 | 3,01E-01 | 1,36E+00 |
| H3f3a | 6,99E-01 | 6,02E-01 | 6,99E-01 | 6,02E-01 | 4,34E-07 | 6,02E-01 | 1,36E+00 |
| Vdac1 | 6,02E-01 | 6,99E-01 | 6,99E-01 | 6,99E-01 | 4,34E-07 | 4,77E-01 | 1,36E+00 |
| Psma7 | 7,78E-01 | 4,77E-01 | 4,77E-01 | 6,99E-01 | 4,34E-07 | 6,99E-01 | 1,36E+00 |
| Flna | 8,45E-01 | 6,99E-01 | 3,01E-01 | 3,01E-01 | 5,55E-01 | 5,30E-01 | 1,36E+00 |
| Dstn | 4,77E-01 | 6,02E-01 | 4,77E-01 | 6,99E-01 | 5,87E-01 | 6,02E-01 | 1,36E+00 |
| Itgb2 | 7,78E-01 | 4,34E-07 | 5,94E-01 | 6,02E-01 | 5,89E-01 | 6,01E-01 | 1,36E+00 |
| Sfn | 4,77E-01 | 7,78E-01 | 5,58E-01 | 5,69E-01 | 5,29E-01 | 4,77E-01 | 1,36E+00 |
| Cnp | 6,99E-01 | 6,02E-01 | 4,77E-01 | 6,99E-01 | 5,59E-01 | 3,01E-01 | 1,35E+00 |
| Fkbp10 | 7,78E-01 | 6,02E-01 | 4,77E-01 | 6,02E-01 | 5,54E-01 | 3,01E-01 | 1,35E+00 |
| Itga6 | 6,99E-01 | 6,99E-01 | 4,77E-01 | 6,99E-01 | 5,47E-01 | 4,34E-07 | 1,35E+00 |
| Sgcd | 4,77E-01 | 4,77E-01 | 6,02E-01 | 6,99E-01 | 5,67E-01 | 5,81E-01 | 1,35E+00 |
| Sec23ip | 7,78E-01 | 6,02E-01 | 6,02E-01 | 4,77E-01 | 5,36E-01 | 3,01E-01 | 1,35E+00 |
| Ppp2r1a | 6,02E-01 | 6,02E-01 | 4,77E-01 | 7,78E-01 | 5,34E-01 | 3,01E-01 | 1,35E+00 |
| Ddah1 | 6,02E-01 | 8,45E-01 | 3,01E-01 | 6,02E-01 | 5,31E-01 | 3,01E-01 | 1,35E+00 |
| Tpm3 | 4,77E-01 | 7,78E-01 | 4,77E-01 | 6,02E-01 | 5,17E-01 | 4,77E-01 | 1,35E+00 |
| P4ha2 | 6,99E-01 | 6,02E-01 | 4,77E-01 | 4,77E-01 | 5,61E-01 | 5,58E-01 | 1,35E+00 |
| Rpl13a | 6,99E-01 | 4,77E-01 | 6,02E-01 | 5,84E-01 | 5,34E-01 | 4,77E-01 | 1,35E+00 |
| Phex | 9,03E-01 | 3,01E-01 | 4,34E-07 | 6,99E-01 | 5,05E-01 | 4,77E-01 | 1,35E+00 |
| Cask | 6,02E-01 | 3,01E-01 | 4,77E-01 | 7,78E-01 | 5,49E-01 | 5,48E-01 | 1,34E+00 |
| Ctnnb1 | 6,99E-01 | 4,77E-01 | 4,77E-01 | 6,02E-01 | 5,47E-01 | 5,47E-01 | 1,34E+00 |
| Clic4 | 6,02E-01 | 6,02E-01 | 4,77E-01 | 7,78E-01 | 4,34E-07 | 6,02E-01 | 1,34E+00 |
| Itga10 | 6,02E-01 | 6,99E-01 | 6,99E-01 | 6,99E-01 | 3,01E-01 | 4,34E-07 | 1,34E+00 |
| Slc26a2 | 6,02E-01 | 6,02E-01 | 4,77E-01 | 6,02E-01 | 4,77E-01 | 6,02E-01 | 1,34E+00 |
| Rps19 | 6,99E-01 | 6,99E-01 | 3,01E-01 | 6,02E-01 | 5,61E-01 | 3,01E-01 | 1,34E+00 |
| Fgfr3 | 6,02E-01 | 6,02E-01 | 3,01E-01 | 7,78E-01 | 5,54E-01 | 3,01E-01 | 1,33E+00 |
| Erp29 | 6,02E-01 | 6,02E-01 | 6,02E-01 | 3,01E-01 | 5,75E-01 | 5,69E-01 | 1,33E+00 |
| Gna12 | 3,01E-01 | 4,77E-01 | 6,99E-01 | 6,02E-01 | 5,62E-01 | 5,78E-01 | 1,33E+00 |
| Arf5 | 6,02E-01 | 6,99E-01 | 6,02E-01 | 4,77E-01 | 5,31E-01 | 3,01E-01 | 1,33E+00 |
| Tgm1 | 6,99E-01 | 6,02E-01 | 4,77E-01 | 4,77E-01 | 5,22E-01 | 4,77E-01 | 1,33E+00 |
| Wdr1 | 4,77E-01 | 6,02E-01 | 6,99E-01 | 6,99E-01 | 5,14E-01 | 4,34E-07 | 1,33E+00 |
| Rab8b | 6,99E-01 | 4,77E-01 | 6,02E-01 | 6,02E-01 | 5,07E-01 | 3,01E-01 | 1,33E+00 |
| Colec12 | 6,02E-01 | 6,02E-01 | 4,77E-01 | 8,45E-01 | 4,34E-07 | 3,01E-01 | 1,32E+00 |
| Nptn | 6,02E-01 | 6,99E-01 | 6,99E-01 | 4,77E-01 | 4,34E-07 | 4,77E-01 | 1,32E+00 |
| Tm9sf2 | 6,99E-01 | 6,02E-01 | 4,77E-01 | 6,99E-01 | 4,34E-07 | 4,77E-01 | 1,32E+00 |
| Sod1 | 4,77E-01 | 6,02E-01 | 4,77E-01 | 6,02E-01 | 4,77E-01 | 6,02E-01 | 1,32E+00 |
| Col2a1 | 6,02E-01 | 6,02E-01 | 4,77E-01 | 4,77E-01 | 5,32E-01 | 5,33E-01 | 1,32E+00 |
| Ikbip | 6,02E-01 | 4,77E-01 | 4,77E-01 | 6,02E-01 | 5,28E-01 | 5,28E-01 | 1,32E+00 |
| H2-D1 | 7,78E-01 | 4,77E-01 | 4,77E-01 | 6,99E-01 | 4,38E-01 | 4,34E-07 | 1,32E+00 |
| Vat1 | 6,02E-01 | 6,02E-01 | 3,01E-01 | 6,02E-01 | 5,51E-01 | 4,77E-01 | 1,31E+00 |
| Capn2 | 4,77E-01 | 4,77E-01 | 4,77E-01 | 6,99E-01 | 5,10E-01 | 5,14E-01 | 1,31E+00 |
| Stx4a | 4,77E-01 | 4,77E-01 | 3,01E-01 | 7,78E-01 | 4,91E-01 | 5,03E-01 | 1,31E+00 |
| Rab5a | 4,77E-01 | 6,02E-01 | 6,02E-01 | 4,77E-01 | 5,12E-01 | 4,77E-01 | 1,31E+00 |
| Apmap | 6,02E-01 | 6,02E-01 | 4,77E-01 | 6,02E-01 | 5,11E-01 | 3,01E-01 | 1,31E+00 |
| Ppic | 3,01E-01 | 4,77E-01 | 6,99E-01 | 6,02E-01 | 5,10E-01 | 4,77E-01 | 1,31E+00 |
| Slc44a1 | 4,77E-01 | 6,02E-01 | 6,02E-01 | 6,02E-01 | 5,07E-01 | 3,01E-01 | 1,31E+00 |
| Cd47 | 6,02E-01 | 6,02E-01 | 4,77E-01 | 4,77E-01 | 5,06E-01 | 4,77E-01 | 1,31E+00 |
| Slc13a5 | 6,02E-01 | 6,02E-01 | 4,77E-01 | 4,77E-01 | 5,06E-01 | 4,77E-01 | 1,31E+00 |
| Rhog | 6,02E-01 | 4,77E-01 | 4,77E-01 | 6,02E-01 | 5,05E-01 | 4,77E-01 | 1,31E+00 |
| Pgd | 4,77E-01 | 7,78E-01 | 3,01E-01 | 4,77E-01 | 4,89E-01 | 4,92E-01 | 1,31E+00 |
| Srm | 6,02E-01 | 4,77E-01 | 4,77E-01 | 6,02E-01 | 5,03E-01 | 4,77E-01 | 1,31E+00 |
| Lamp1 | 6,99E-01 | 4,77E-01 | 4,77E-01 | 4,77E-01 | 5,02E-01 | 4,77E-01 | 1,30E+00 |
| Rab5c | 6,02E-01 | 4,77E-01 | 4,77E-01 | 6,02E-01 | 4,98E-01 | 4,77E-01 | 1,30E+00 |
| Rftn2 | 4,77E-01 | 6,02E-01 | 6,02E-01 | 6,99E-01 | 4,97E-01 | 4,34E-07 | 1,30E+00 |
| Psma5 | 6,02E-01 | 7,78E-01 | 3,01E-01 | 6,02E-01 | 4,90E-01 | 4,34E-07 | 1,30E+00 |
| Panx3 | 4,77E-01 | 6,02E-01 | 6,99E-01 | 4,77E-01 | 4,34E-07 | 6,02E-01 | 1,30E+00 |
| H2az1 | 4,77E-01 | 4,77E-01 | 6,02E-01 | 6,02E-01 | 4,77E-01 | 4,77E-01 | 1,30E+00 |
| Cdh13 | 6,02E-01 | 3,01E-01 | 4,77E-01 | 9,03E-01 | 4,34E-07 | 3,01E-01 | 1,30E+00 |
| Cfl1 | 4,77E-01 | 4,77E-01 | 4,77E-01 | 7,78E-01 | 3,01E-01 | 4,77E-01 | 1,30E+00 |
| Adk | 6,99E-01 | 3,01E-01 | 6,02E-01 | 6,02E-01 | 4,34E-07 | 6,02E-01 | 1,30E+00 |
| Insrr | 6,02E-01 | 4,77E-01 | 6,02E-01 | 3,01E-01 | 4,77E-01 | 6,02E-01 | 1,30E+00 |
| Phb1 | 6,02E-01 | 3,01E-01 | 4,77E-01 | 6,02E-01 | 5,27E-01 | 5,06E-01 | 1,29E+00 |
| Chp1 | 6,02E-01 | 4,77E-01 | 3,01E-01 | 6,02E-01 | 5,05E-01 | 4,94E-01 | 1,29E+00 |
| Inpp5a | 4,77E-01 | 6,99E-01 | 4,77E-01 | 3,01E-01 | 4,91E-01 | 5,08E-01 | 1,29E+00 |
| Ihh | 4,77E-01 | 4,77E-01 | 3,01E-01 | 6,02E-01 | 5,16E-01 | 6,02E-01 | 1,29E+00 |
| P3h1 | 6,99E-01 | 3,01E-01 | 4,77E-01 | 4,77E-01 | 4,99E-01 | 4,93E-01 | 1,28E+00 |
| Apoa1 | 3,01E-01 | 6,99E-01 | 4,77E-01 | 4,77E-01 | 4,91E-01 | 4,95E-01 | 1,28E+00 |
| Rap1b | 4,77E-01 | 4,77E-01 | 6,02E-01 | 4,77E-01 | 4,94E-01 | 4,77E-01 | 1,28E+00 |
| Rps4x | 6,99E-01 | 4,77E-01 | 3,01E-01 | 4,77E-01 | 4,89E-01 | 4,77E-01 | 1,28E+00 |
| Cox4i1 | 6,02E-01 | 6,02E-01 | 4,77E-01 | 6,99E-01 | 4,34E-07 | 3,01E-01 | 1,28E+00 |
| Dag1 | 6,02E-01 | 4,77E-01 | 6,02E-01 | 6,99E-01 | 4,34E-07 | 3,01E-01 | 1,28E+00 |
| Hbb-bs | 6,99E-01 | 4,77E-01 | 3,01E-01 | 6,99E-01 | 4,34E-07 | 4,77E-01 | 1,28E+00 |
| Arg1 | 4,77E-01 | 6,02E-01 | 6,99E-01 | 4,77E-01 | 3,01E-01 | 3,01E-01 | 1,28E+00 |
| Gng12 | 6,02E-01 | 6,02E-01 | 4,77E-01 | 6,99E-01 | 4,34E-07 | 3,01E-01 | 1,28E+00 |
| Cd82 | 6,02E-01 | 6,99E-01 | 3,01E-01 | 4,77E-01 | 4,34E-07 | 6,02E-01 | 1,28E+00 |
| Pdia6 | 4,77E-01 | 6,02E-01 | 6,99E-01 | 6,02E-01 | 4,34E-07 | 3,01E-01 | 1,28E+00 |
| Plec | 6,99E-01 | 6,99E-01 | 4,77E-01 | 3,01E-01 | 3,01E-01 | 3,01E-01 | 1,28E+00 |
| Dlg1 | 6,02E-01 | 4,77E-01 | 6,02E-01 | 6,02E-01 | 4,63E-01 | 4,34E-07 | 1,28E+00 |
| Rps18 | 6,02E-01 | 4,77E-01 | 4,77E-01 | 6,02E-01 | 4,56E-01 | 3,01E-01 | 1,28E+00 |
| Mlec | 6,99E-01 | 4,77E-01 | 6,02E-01 | 3,01E-01 | 4,31E-01 | 3,01E-01 | 1,27E+00 |
| Tsg101 | 4,77E-01 | 7,78E-01 | 3,01E-01 | 6,02E-01 | 4,00E-01 | 4,34E-07 | 1,27E+00 |
| Rpl13 | 3,01E-01 | 3,01E-01 | 4,77E-01 | 6,02E-01 | 5,31E-01 | 6,02E-01 | 1,26E+00 |
| Rras | 4,77E-01 | 3,01E-01 | 6,02E-01 | 4,77E-01 | 4,96E-01 | 5,07E-01 | 1,26E+00 |
| Rps14 | 4,77E-01 | 4,77E-01 | 3,01E-01 | 4,77E-01 | 5,05E-01 | 6,02E-01 | 1,26E+00 |
| Pgm1 | 4,77E-01 | 6,02E-01 | 4,77E-01 | 3,01E-01 | 4,88E-01 | 4,91E-01 | 1,26E+00 |
| Rnh1 | 4,77E-01 | 3,01E-01 | 4,77E-01 | 6,02E-01 | 4,87E-01 | 4,85E-01 | 1,26E+00 |
| Flg2 | 4,77E-01 | 6,02E-01 | 3,01E-01 | 4,77E-01 | 4,80E-01 | 4,77E-01 | 1,26E+00 |
| Dpysl2 | 6,02E-01 | 3,01E-01 | 4,75E-01 | 4,77E-01 | 4,77E-01 | 4,81E-01 | 1,26E+00 |
| Cyb5a | 6,02E-01 | 4,77E-01 | 6,02E-01 | 6,99E-01 | 4,34E-07 | 4,34E-07 | 1,26E+00 |
| 1700009N14Rik | 3,01E-01 | 6,02E-01 | 4,77E-01 | 6,02E-01 | 4,34E-07 | 6,02E-01 | 1,26E+00 |
| Ergic1 | 4,77E-01 | 4,77E-01 | 4,77E-01 | 4,77E-01 | 4,77E-01 | 4,77E-01 | 1,26E+00 |
| Cmpk1 | 4,77E-01 | 4,77E-01 | 4,77E-01 | 4,77E-01 | 4,77E-01 | 4,77E-01 | 1,26E+00 |
| Ckm | 4,77E-01 | 4,77E-01 | 4,77E-01 | 4,77E-01 | 4,77E-01 | 4,77E-01 | 1,26E+00 |
| Rps3 | 4,77E-01 | 4,77E-01 | 4,77E-01 | 4,77E-01 | 4,77E-01 | 4,77E-01 | 1,26E+00 |
| Cd9 | 6,02E-01 | 3,01E-01 | 6,02E-01 | 6,02E-01 | 4,34E-07 | 4,77E-01 | 1,26E+00 |
| S100a10 | 3,01E-01 | 3,01E-01 | 4,77E-01 | 6,02E-01 | 3,01E-01 | 6,99E-01 | 1,26E+00 |
| Atp5pd | 3,01E-01 | 4,77E-01 | 4,77E-01 | 6,02E-01 | 4,71E-01 | 4,77E-01 | 1,25E+00 |
| Rpl10a | 6,02E-01 | 4,77E-01 | 4,77E-01 | 4,77E-01 | 4,68E-01 | 3,01E-01 | 1,25E+00 |
| Napg | 4,77E-01 | 6,02E-01 | 3,01E-01 | 4,77E-01 | 4,73E-01 | 4,68E-01 | 1,25E+00 |
| Gfpt2 | 3,01E-01 | 6,02E-01 | 3,01E-01 | 6,02E-01 | 4,71E-01 | 4,67E-01 | 1,25E+00 |
| Mmp14 | 4,77E-01 | 4,77E-01 | 3,01E-01 | 6,02E-01 | 4,70E-01 | 4,59E-01 | 1,25E+00 |
| Erp44 | 6,02E-01 | 6,02E-01 | 3,01E-01 | 6,02E-01 | 4,50E-01 | 4,34E-07 | 1,25E+00 |
| Rap2c | 6,02E-01 | 4,77E-01 | 3,01E-01 | 6,02E-01 | 4,41E-01 | 3,01E-01 | 1,25E+00 |
| Jsrp1 | 4,87E-01 | 4,77E-01 | 4,77E-01 | 6,02E-01 | 4,28E-01 | 3,01E-01 | 1,25E+00 |
| Itgb3 | 6,02E-01 | 4,77E-01 | 3,01E-01 | 4,77E-01 | 4,56E-01 | 4,53E-01 | 1,25E+00 |
| Uba1 | 6,02E-01 | 4,77E-01 | 3,01E-01 | 6,99E-01 | 4,30E-01 | 4,34E-07 | 1,25E+00 |
| Mgat2 | 4,77E-01 | 4,77E-01 | 3,01E-01 | 6,99E-01 | 4,18E-01 | 3,01E-01 | 1,25E+00 |
| Sdcbp | 4,77E-01 | 3,01E-01 | 6,99E-01 | 6,02E-01 | 4,03E-01 | 4,34E-07 | 1,24E+00 |
| Fasn | 4,77E-01 | 3,01E-01 | 4,34E-07 | 7,78E-01 | 4,49E-01 | 4,23E-01 | 1,24E+00 |
| Mme | 7,78E-01 | 4,77E-01 | 3,01E-01 | 4,34E-07 | 4,37E-01 | 4,17E-01 | 1,24E+00 |
| Vdac2 | 3,01E-01 | 3,01E-01 | 4,77E-01 | 4,77E-01 | 4,94E-01 | 6,02E-01 | 1,23E+00 |
| Dipk2a | 6,02E-01 | 6,02E-01 | 3,01E-01 | 4,77E-01 | 4,34E-07 | 4,77E-01 | 1,23E+00 |
| Arf4 | 4,77E-01 | 4,77E-01 | 6,02E-01 | 6,02E-01 | 4,34E-07 | 3,01E-01 | 1,23E+00 |
| Krt80 | 6,02E-01 | 6,02E-01 | 4,77E-01 | 3,01E-01 | 4,34E-07 | 4,77E-01 | 1,23E+00 |
| Cd200 | 4,77E-01 | 4,77E-01 | 6,02E-01 | 3,01E-01 | 4,34E-07 | 6,02E-01 | 1,23E+00 |
| Prdx2 | 6,02E-01 | 6,02E-01 | 4,77E-01 | 4,77E-01 | 4,34E-07 | 3,01E-01 | 1,23E+00 |
| Rcn3 | 6,02E-01 | 6,02E-01 | 4,77E-01 | 4,77E-01 | 4,34E-07 | 3,01E-01 | 1,23E+00 |
| Rplp0 | 6,02E-01 | 4,77E-01 | 3,01E-01 | 4,77E-01 | 4,38E-01 | 3,01E-01 | 1,22E+00 |
| Rps16 | 4,77E-01 | 4,77E-01 | 3,01E-01 | 6,02E-01 | 4,37E-01 | 3,01E-01 | 1,22E+00 |
| Pitpna | 6,99E-01 | 6,02E-01 | 4,34E-07 | 4,77E-01 | 4,32E-01 | 4,34E-07 | 1,22E+00 |
| Rpl8 | 4,77E-01 | 4,77E-01 | 4,77E-01 | 4,77E-01 | 4,28E-01 | 3,01E-01 | 1,22E+00 |
| Ncam1 | 4,77E-01 | 6,99E-01 | 3,01E-01 | 4,34E-07 | 4,39E-01 | 4,65E-01 | 1,22E+00 |
| Pls3 | 4,77E-01 | 3,01E-01 | 4,55E-01 | 4,77E-01 | 4,63E-01 | 4,57E-01 | 1,22E+00 |
| Rab35 | 4,77E-01 | 6,02E-01 | 4,77E-01 | 3,01E-01 | 4,15E-01 | 3,01E-01 | 1,22E+00 |
| Man1b1 | 4,77E-01 | 6,02E-01 | 4,34E-07 | 4,77E-01 | 4,64E-01 | 4,26E-01 | 1,22E+00 |
| Pygl | 4,77E-01 | 6,02E-01 | 3,01E-01 | 6,02E-01 | 4,04E-01 | 4,34E-07 | 1,22E+00 |
| Rtn4 | 4,34E-07 | 6,02E-01 | 4,77E-01 | 6,02E-01 | 3,99E-01 | 3,01E-01 | 1,22E+00 |
| Npepps | 3,01E-01 | 4,77E-01 | 4,45E-01 | 4,77E-01 | 4,50E-01 | 4,49E-01 | 1,22E+00 |
| Fut8 | 6,02E-01 | 4,77E-01 | 4,41E-01 | 4,34E-07 | 4,61E-01 | 4,39E-01 | 1,21E+00 |
| Tmed4 | 4,77E-01 | 4,77E-01 | 4,34E-01 | 3,01E-01 | 4,54E-01 | 4,46E-01 | 1,21E+00 |
| Cyfip1 | 6,02E-01 | 6,02E-01 | 4,34E-07 | 3,01E-01 | 4,52E-01 | 4,00E-01 | 1,21E+00 |
| Vcp | 6,02E-01 | 4,77E-01 | 4,34E-07 | 4,77E-01 | 4,40E-01 | 4,11E-01 | 1,21E+00 |
| Rps3a1 | 6,99E-01 | 3,01E-01 | 4,77E-01 | 4,77E-01 | 3,55E-01 | 4,34E-07 | 1,21E+00 |
| Fermt2 | 6,02E-01 | 4,77E-01 | 4,34E-07 | 4,77E-01 | 4,33E-01 | 3,97E-01 | 1,21E+00 |
| Tmem30a | 4,77E-01 | 3,01E-01 | 3,01E-01 | 6,02E-01 | 4,18E-01 | 4,10E-01 | 1,21E+00 |
| Tm9sf1 | 7,78E-01 | 3,01E-01 | 4,34E-07 | 6,02E-01 | 3,33E-01 | 4,34E-07 | 1,21E+00 |
| Arpc1b | 4,77E-01 | 4,77E-01 | 4,34E-07 | 6,02E-01 | 4,26E-01 | 3,91E-01 | 1,21E+00 |
| Arpc2 | 6,02E-01 | 3,01E-01 | 3,01E-01 | 4,77E-01 | 4,03E-01 | 3,97E-01 | 1,20E+00 |
| Rab11b | 6,02E-01 | 4,77E-01 | 6,99E-01 | 4,34E-07 | 3,01E-01 | 4,34E-07 | 1,20E+00 |
| Ahcy | 6,02E-01 | 6,99E-01 | 3,01E-01 | 4,77E-01 | 4,34E-07 | 4,34E-07 | 1,20E+00 |
| Atp5pb | 6,02E-01 | 4,77E-01 | 3,01E-01 | 6,02E-01 | 4,34E-07 | 3,01E-01 | 1,20E+00 |
| Tmem43 | 6,02E-01 | 4,77E-01 | 4,77E-01 | 6,02E-01 | 4,34E-07 | 4,34E-07 | 1,20E+00 |
| Gm45717 | 3,01E-01 | 4,77E-01 | 4,77E-01 | 3,01E-01 | 3,01E-01 | 6,02E-01 | 1,20E+00 |
| Tspan6 | 6,02E-01 | 3,01E-01 | 4,77E-01 | 6,02E-01 | 4,34E-07 | 3,01E-01 | 1,20E+00 |
| Sdk2 | 7,78E-01 | 6,02E-01 | 4,34E-07 | 3,01E-01 | 2,95E-01 | 4,34E-07 | 1,20E+00 |
| Cd36 | 4,77E-01 | 4,34E-07 | 3,01E-01 | 4,77E-01 | 4,62E-01 | 6,02E-01 | 1,20E+00 |
| Arl8b | 4,77E-01 | 4,77E-01 | 3,01E-01 | 4,77E-01 | 4,15E-01 | 3,01E-01 | 1,19E+00 |
| Copb1 | 4,77E-01 | 4,77E-01 | 3,01E-01 | 4,77E-01 | 4,12E-01 | 3,01E-01 | 1,19E+00 |
| Slc5a3 | 4,77E-01 | 4,77E-01 | 4,77E-01 | 3,01E-01 | 4,03E-01 | 3,01E-01 | 1,19E+00 |
| Banf1 | 4,77E-01 | 6,02E-01 | 4,77E-01 | 4,34E-07 | 3,94E-01 | 3,01E-01 | 1,19E+00 |
| Tpt1 | 4,77E-01 | 4,77E-01 | 3,01E-01 | 6,02E-01 | 3,88E-01 | 4,34E-07 | 1,19E+00 |
| Prkcsh | 4,77E-01 | 4,77E-01 | 4,77E-01 | 4,77E-01 | 3,80E-01 | 4,34E-07 | 1,19E+00 |
| Hhatl | 4,34E-07 | 3,01E-01 | 4,77E-01 | 6,99E-01 | 3,78E-01 | 3,01E-01 | 1,19E+00 |
| Ykt6 | 6,02E-01 | 4,77E-01 | 4,77E-01 | 3,01E-01 | 3,75E-01 | 4,34E-07 | 1,19E+00 |
| Stx2 | 4,77E-01 | 4,77E-01 | 4,77E-01 | 4,77E-01 | 3,71E-01 | 4,34E-07 | 1,19E+00 |
| Sdf4 | 4,77E-01 | 4,77E-01 | 4,34E-07 | 6,99E-01 | 3,64E-01 | 4,34E-07 | 1,19E+00 |
| Rab14 | 6,02E-01 | 4,77E-01 | 4,77E-01 | 3,01E-01 | 3,56E-01 | 4,34E-07 | 1,18E+00 |
| Nherf2 | 4,77E-01 | 6,02E-01 | 4,34E-07 | 6,02E-01 | 3,50E-01 | 4,34E-07 | 1,18E+00 |
| Rpl3 | 8,45E-01 | 3,01E-01 | 4,34E-07 | 3,01E-01 | 3,46E-01 | 4,34E-07 | 1,18E+00 |
| Slc29a1 | 4,77E-01 | 3,01E-01 | 3,01E-01 | 4,77E-01 | 3,01E-01 | 4,77E-01 | 1,18E+00 |
| Tmem165 | 4,77E-01 | 3,01E-01 | 4,77E-01 | 4,77E-01 | 4,34E-07 | 4,77E-01 | 1,18E+00 |
| Hba-a2 | 4,77E-01 | 4,77E-01 | 3,01E-01 | 4,77E-01 | 4,34E-07 | 4,77E-01 | 1,18E+00 |
| Slc38a3 | 6,02E-01 | 3,01E-01 | 4,77E-01 | 4,77E-01 | 4,34E-07 | 3,01E-01 | 1,18E+00 |
| Lamp2 | 3,01E-01 | 4,77E-01 | 3,01E-01 | 6,02E-01 | 3,01E-01 | 3,01E-01 | 1,18E+00 |
| Uggt1 | 4,77E-01 | 3,01E-01 | 3,01E-01 | 4,77E-01 | 4,02E-01 | 3,92E-01 | 1,18E+00 |
| Ckb | 3,01E-01 | 3,01E-01 | 3,79E-01 | 6,02E-01 | 3,46E-01 | 3,62E-01 | 1,17E+00 |
| Capns1 | 4,77E-01 | 3,01E-01 | 3,01E-01 | 4,77E-01 | 3,91E-01 | 3,86E-01 | 1,17E+00 |
| Ephb2 | 4,77E-01 | 4,77E-01 | 3,01E-01 | 3,01E-01 | 3,82E-01 | 3,83E-01 | 1,17E+00 |
| Erlin2 | 4,77E-01 | 3,01E-01 | 3,01E-01 | 4,77E-01 | 3,85E-01 | 3,70E-01 | 1,17E+00 |
| Pals2 | 3,01E-01 | 6,02E-01 | 4,34E-07 | 4,77E-01 | 3,89E-01 | 3,60E-01 | 1,17E+00 |
| Atp6v0a1 | 3,01E-01 | 4,77E-01 | 4,34E-07 | 6,02E-01 | 3,77E-01 | 3,61E-01 | 1,17E+00 |
| Car12 | 3,01E-01 | 4,34E-07 | 4,77E-01 | 4,77E-01 | 4,21E-01 | 4,77E-01 | 1,17E+00 |
| Krt82 | 4,34E-07 | 4,77E-01 | 4,12E-01 | 3,45E-01 | 4,77E-01 | 4,34E-01 | 1,16E+00 |
| Fam3c | 4,77E-01 | 4,34E-07 | 4,77E-01 | 4,77E-01 | 3,76E-01 | 3,01E-01 | 1,16E+00 |
| Acsl1 | 3,01E-01 | 3,72E-01 | 3,01E-01 | 6,99E-01 | 2,98E-01 | 4,34E-07 | 1,16E+00 |
| Hsd17b12 | 4,77E-01 | 4,77E-01 | 4,34E-07 | 6,02E-01 | 3,69E-01 | 4,34E-07 | 1,16E+00 |
| Fth1 | 3,01E-01 | 4,77E-01 | 3,57E-01 | 6,02E-01 | 3,07E-01 | 4,34E-07 | 1,16E+00 |
| Sfxn3 | 6,02E-01 | 4,34E-07 | 4,34E-07 | 6,02E-01 | 3,47E-01 | 3,10E-01 | 1,15E+00 |
| Ssr4 | 4,77E-01 | 3,01E-01 | 4,77E-01 | 3,01E-01 | 3,53E-01 | 3,01E-01 | 1,15E+00 |
| Psma6 | 4,77E-01 | 3,01E-01 | 4,77E-01 | 3,01E-01 | 3,52E-01 | 3,01E-01 | 1,15E+00 |
| Pitpnb | 4,77E-01 | 3,01E-01 | 4,77E-01 | 4,77E-01 | 3,49E-01 | 4,34E-07 | 1,15E+00 |
| Aqp1 | 4,77E-01 | 4,77E-01 | 3,01E-01 | 3,01E-01 | 3,47E-01 | 3,01E-01 | 1,15E+00 |
| Itgb5 | 6,02E-01 | 3,01E-01 | 3,46E-01 | 6,02E-01 | 4,34E-07 | 4,34E-07 | 1,15E+00 |
| Rala | 3,01E-01 | 3,01E-01 | 4,77E-01 | 4,77E-01 | 3,44E-01 | 3,01E-01 | 1,15E+00 |
| Rpl27a | 4,77E-01 | 4,77E-01 | 4,34E-07 | 6,02E-01 | 3,40E-01 | 4,34E-07 | 1,15E+00 |
| Ist1 | 3,01E-01 | 4,77E-01 | 4,77E-01 | 4,77E-01 | 3,32E-01 | 4,34E-07 | 1,15E+00 |
| Antxr2 | 4,77E-01 | 3,01E-01 | 4,77E-01 | 4,77E-01 | 3,25E-01 | 4,34E-07 | 1,15E+00 |
| Sh3gl1 | 6,02E-01 | 4,77E-01 | 3,01E-01 | 3,01E-01 | 3,18E-01 | 4,34E-07 | 1,15E+00 |
| Emb | 4,77E-01 | 3,01E-01 | 3,01E-01 | 4,77E-01 | 4,34E-07 | 4,77E-01 | 1,15E+00 |
| Psma3 | 4,77E-01 | 4,77E-01 | 3,01E-01 | 4,77E-01 | 4,34E-07 | 3,01E-01 | 1,15E+00 |
| S100a11 | 3,01E-01 | 3,01E-01 | 4,77E-01 | 4,77E-01 | 3,01E-01 | 3,01E-01 | 1,15E+00 |
| Pdcd6 | 3,01E-01 | 6,02E-01 | 4,34E-07 | 6,02E-01 | 2,96E-01 | 4,34E-07 | 1,15E+00 |
| Adipoq | 3,01E-01 | 3,01E-01 | 6,02E-01 | 4,77E-01 | 2,91E-01 | 4,34E-07 | 1,14E+00 |
| Tns2 | 4,77E-01 | 4,77E-01 | 3,01E-01 | 4,34E-07 | 3,79E-01 | 3,70E-01 | 1,14E+00 |
| Fdps | 4,77E-01 | 4,77E-01 | 4,34E-07 | 3,01E-01 | 3,75E-01 | 3,44E-01 | 1,13E+00 |
| Rpl23a | 4,77E-01 | 3,01E-01 | 3,55E-01 | 3,01E-01 | 3,31E-01 | 3,34E-01 | 1,13E+00 |
| Ddah2 | 4,34E-07 | 4,77E-01 | 3,01E-01 | 4,77E-01 | 3,57E-01 | 3,56E-01 | 1,13E+00 |
| Ptma | 3,01E-01 | 3,01E-01 | 4,77E-01 | 3,01E-01 | 3,44E-01 | 3,67E-01 | 1,13E+00 |
| H1f2 | 3,56E-01 | 3,01E-01 | 3,01E-01 | 4,77E-01 | 3,01E-01 | 3,37E-01 | 1,13E+00 |
| Cab39l | 3,01E-01 | 4,77E-01 | 3,01E-01 | 3,01E-01 | 3,33E-01 | 3,38E-01 | 1,12E+00 |
| Ank | 3,01E-01 | 4,77E-01 | 3,01E-01 | 3,01E-01 | 3,33E-01 | 3,37E-01 | 1,12E+00 |
| Actr3 | 3,01E-01 | 3,01E-01 | 3,01E-01 | 4,77E-01 | 3,29E-01 | 3,36E-01 | 1,12E+00 |
| Pdcd10 | 3,01E-01 | 4,77E-01 | 3,01E-01 | 3,01E-01 | 3,28E-01 | 3,33E-01 | 1,12E+00 |
| Rpl35 | 3,01E-01 | 3,01E-01 | 3,01E-01 | 4,77E-01 | 3,23E-01 | 3,34E-01 | 1,12E+00 |
| Zmpste24 | 4,77E-01 | 3,01E-01 | 3,01E-01 | 3,01E-01 | 3,26E-01 | 3,24E-01 | 1,12E+00 |
| Phb2 | 3,01E-01 | 3,28E-01 | 4,77E-01 | 4,77E-01 | 4,34E-07 | 3,01E-01 | 1,12E+00 |
| Sntb2 | 3,01E-01 | 4,34E-07 | 4,34E-07 | 6,99E-01 | 2,91E-01 | 3,33E-01 | 1,12E+00 |
| Mapk1 | 4,77E-01 | 3,01E-01 | 3,14E-01 | 4,77E-01 | 3,06E-01 | 4,34E-07 | 1,12E+00 |
| Rab18 | 4,77E-01 | 3,01E-01 | 4,34E-07 | 4,77E-01 | 3,16E-01 | 3,01E-01 | 1,12E+00 |
| Rpl27 | 6,99E-01 | 3,01E-01 | 4,34E-07 | 3,01E-01 | 3,05E-01 | 4,34E-07 | 1,11E+00 |
| Slc38a4 | 4,34E-07 | 6,02E-01 | 4,34E-07 | 4,77E-01 | 3,01E-01 | 3,01E-01 | 1,11E+00 |
| Ide | 6,02E-01 | 6,99E-01 | 4,34E-07 | 4,34E-07 | 4,34E-07 | 4,34E-07 | 1,11E+00 |
| Mdh1 | 4,77E-01 | 4,77E-01 | 3,01E-01 | 3,01E-01 | 3,01E-01 | 4,34E-07 | 1,11E+00 |
| Tmx1 | 4,77E-01 | 4,77E-01 | 3,01E-01 | 3,01E-01 | 4,34E-07 | 3,01E-01 | 1,11E+00 |
| 9530068E07Rik | 3,01E-01 | 4,77E-01 | 3,01E-01 | 3,01E-01 | 3,01E-01 | 3,01E-01 | 1,11E+00 |
| Zdhhc20 | 4,77E-01 | 4,77E-01 | 3,01E-01 | 3,01E-01 | 2,73E-01 | 4,34E-07 | 1,11E+00 |
| Ssr1 | 4,77E-01 | 4,77E-01 | 3,01E-01 | 3,01E-01 | 2,73E-01 | 4,34E-07 | 1,11E+00 |
| Ror2 | 4,77E-01 | 3,01E-01 | 4,34E-07 | 6,02E-01 | 2,69E-01 | 4,34E-07 | 1,11E+00 |
| Sec11a | 4,77E-01 | 3,01E-01 | 3,01E-01 | 4,77E-01 | 2,56E-01 | 4,34E-07 | 1,11E+00 |
| Slc7a6 | 3,01E-01 | 4,77E-01 | 3,01E-01 | 4,77E-01 | 2,52E-01 | 4,34E-07 | 1,11E+00 |
| Rplp2 | 4,77E-01 | 6,02E-01 | 4,34E-07 | 3,01E-01 | 2,40E-01 | 4,34E-07 | 1,11E+00 |
| Parva | 3,01E-01 | 4,77E-01 | 3,18E-01 | 4,34E-07 | 3,16E-01 | 3,06E-01 | 1,09E+00 |
| Slc7a1 | 4,34E-07 | 3,01E-01 | 4,34E-07 | 4,77E-01 | 3,37E-01 | 4,77E-01 | 1,09E+00 |
| Tpm4 | 3,01E-01 | 4,34E-07 | 3,06E-01 | 4,77E-01 | 3,12E-01 | 3,08E-01 | 1,08E+00 |
| Lpar1 | 3,01E-01 | 4,77E-01 | 3,01E-01 | 4,34E-07 | 3,11E-01 | 3,01E-01 | 1,08E+00 |
| Vtn | 4,34E-07 | 3,01E-01 | 3,05E-01 | 4,77E-01 | 2,98E-01 | 3,10E-01 | 1,08E+00 |
| Slc7a2 | 4,34E-07 | 3,01E-01 | 4,77E-01 | 3,01E-01 | 2,92E-01 | 3,17E-01 | 1,08E+00 |
| Rpl35a | 4,77E-01 | 3,01E-01 | 3,01E-01 | 4,34E-07 | 3,06E-01 | 3,01E-01 | 1,08E+00 |
| Emc1 | 3,01E-01 | 4,34E-07 | 3,04E-01 | 4,77E-01 | 3,02E-01 | 3,00E-01 | 1,08E+00 |
| Sec61b | 4,77E-01 | 3,01E-01 | 3,01E-01 | 3,01E-01 | 4,34E-07 | 3,01E-01 | 1,08E+00 |
| Cd44 | 4,77E-01 | 4,77E-01 | 3,01E-01 | 3,01E-01 | 4,34E-07 | 4,34E-07 | 1,08E+00 |
| Golm1 | 3,01E-01 | 3,01E-01 | 3,01E-01 | 3,01E-01 | 3,01E-01 | 3,01E-01 | 1,08E+00 |
| Slc38a2 | 3,01E-01 | 3,01E-01 | 3,01E-01 | 3,01E-01 | 4,34E-07 | 4,77E-01 | 1,08E+00 |
| Serinc5 | 3,01E-01 | 3,01E-01 | 3,01E-01 | 3,01E-01 | 4,34E-07 | 4,77E-01 | 1,08E+00 |
| Ephb3 | 4,77E-01 | 6,02E-01 | 3,01E-01 | 4,34E-07 | 4,34E-07 | 4,34E-07 | 1,08E+00 |
| Gsdma | 4,77E-01 | 3,01E-01 | 4,77E-01 | 4,34E-07 | 4,34E-07 | 3,01E-01 | 1,08E+00 |
| Uqcrfs1 | 3,01E-01 | 3,01E-01 | 3,01E-01 | 3,01E-01 | 3,01E-01 | 3,01E-01 | 1,08E+00 |
| Hrc | 3,01E-01 | 3,01E-01 | 3,01E-01 | 3,01E-01 | 3,01E-01 | 3,01E-01 | 1,08E+00 |
| Psmb1 | 3,01E-01 | 3,01E-01 | 3,01E-01 | 3,01E-01 | 3,01E-01 | 3,01E-01 | 1,08E+00 |
| M6pr | 3,01E-01 | 4,77E-01 | 4,34E-07 | 4,77E-01 | 4,34E-07 | 3,01E-01 | 1,08E+00 |
| Clic1 | 3,01E-01 | 4,77E-01 | 4,34E-07 | 6,02E-01 | 4,34E-07 | 4,34E-07 | 1,08E+00 |
| Slc1a4 | 3,01E-01 | 4,77E-01 | 3,01E-01 | 3,01E-01 | 4,34E-07 | 3,01E-01 | 1,08E+00 |
| Evpl | 3,01E-01 | 3,01E-01 | 3,01E-01 | 3,01E-01 | 3,01E-01 | 3,01E-01 | 1,08E+00 |
| S100a14 | 3,01E-01 | 3,01E-01 | 3,01E-01 | 3,01E-01 | 3,01E-01 | 3,01E-01 | 1,08E+00 |
| Ncstn | 3,01E-01 | 3,01E-01 | 3,01E-01 | 3,01E-01 | 3,01E-01 | 3,01E-01 | 1,08E+00 |
| Gm49369 | 3,01E-01 | 3,01E-01 | 3,01E-01 | 3,01E-01 | 3,01E-01 | 3,01E-01 | 1,08E+00 |
| Serpina3k | 3,01E-01 | 3,01E-01 | 3,01E-01 | 3,01E-01 | 3,01E-01 | 3,01E-01 | 1,08E+00 |
| Tsn | 3,01E-01 | 3,01E-01 | 3,01E-01 | 3,01E-01 | 3,01E-01 | 3,01E-01 | 1,08E+00 |
| Fam234a | 3,01E-01 | 4,77E-01 | 3,01E-01 | 3,01E-01 | 4,34E-07 | 3,01E-01 | 1,08E+00 |
| Acp5 | 3,01E-01 | 3,01E-01 | 3,01E-01 | 3,01E-01 | 3,01E-01 | 3,01E-01 | 1,08E+00 |
| Atp5f1d | 3,01E-01 | 3,01E-01 | 3,01E-01 | 3,01E-01 | 3,01E-01 | 3,01E-01 | 1,08E+00 |
| Rpl19 | 3,01E-01 | 3,01E-01 | 3,01E-01 | 3,01E-01 | 3,01E-01 | 3,01E-01 | 1,08E+00 |
| Ndrg2 | 3,01E-01 | 3,01E-01 | 3,01E-01 | 4,77E-01 | 4,34E-07 | 3,01E-01 | 1,08E+00 |
| Chmp4b | 4,34E-07 | 4,34E-07 | 4,77E-01 | 4,77E-01 | 2,96E-01 | 3,01E-01 | 1,08E+00 |
| Kctd4 | 4,34E-07 | 3,01E-01 | 2,92E-01 | 4,77E-01 | 2,99E-01 | 3,07E-01 | 1,08E+00 |
| Mtap | 4,34E-07 | 3,01E-01 | 4,77E-01 | 3,01E-01 | 2,95E-01 | 3,01E-01 | 1,08E+00 |
| Capza1 | 3,01E-01 | 4,77E-01 | 4,34E-07 | 3,01E-01 | 2,96E-01 | 2,83E-01 | 1,08E+00 |
| Dnajb11 | 4,77E-01 | 4,77E-01 | 4,34E-07 | 4,34E-07 | 2,98E-01 | 2,79E-01 | 1,08E+00 |
| Atp5f1c | 3,01E-01 | 4,77E-01 | 3,01E-01 | 3,01E-01 | 2,75E-01 | 4,34E-07 | 1,08E+00 |
| Eef1g | 3,01E-01 | 4,77E-01 | 3,01E-01 | 4,34E-07 | 2,92E-01 | 2,84E-01 | 1,07E+00 |
| Ndufa13 | 3,01E-01 | 3,01E-01 | 4,34E-07 | 4,77E-01 | 2,92E-01 | 2,81E-01 | 1,07E+00 |
| Entpd3 | 4,77E-01 | 3,01E-01 | 4,34E-07 | 3,01E-01 | 3,07E-01 | 2,62E-01 | 1,07E+00 |
| Tmem181a | 3,01E-01 | 3,01E-01 | 4,34E-07 | 4,77E-01 | 2,80E-01 | 2,87E-01 | 1,07E+00 |
| Cst3 | 3,01E-01 | 3,01E-01 | 4,77E-01 | 3,01E-01 | 2,64E-01 | 4,34E-07 | 1,07E+00 |
| Ahcyl2 | 4,77E-01 | 4,34E-07 | 2,95E-01 | 3,01E-01 | 2,86E-01 | 2,83E-01 | 1,07E+00 |
| Neo1 | 4,77E-01 | 3,01E-01 | 3,01E-01 | 3,01E-01 | 2,60E-01 | 4,34E-07 | 1,07E+00 |
| Slc31a1 | 4,77E-01 | 3,01E-01 | 2,84E-01 | 4,34E-07 | 2,88E-01 | 2,89E-01 | 1,07E+00 |
| Txndc5 | 3,01E-01 | 3,01E-01 | 4,34E-07 | 4,77E-01 | 2,82E-01 | 2,76E-01 | 1,07E+00 |
| Ranbp1 | 3,01E-01 | 6,02E-01 | 4,34E-07 | 4,34E-07 | 2,88E-01 | 2,68E-01 | 1,07E+00 |
| Pon3 | 3,01E-01 | 3,01E-01 | 3,01E-01 | 4,77E-01 | 2,49E-01 | 4,34E-07 | 1,07E+00 |
| Mcfd2 | 3,01E-01 | 3,01E-01 | 4,34E-07 | 4,77E-01 | 2,89E-01 | 2,61E-01 | 1,07E+00 |
| Atp6v0a2 | 6,02E-01 | 3,01E-01 | 3,01E-01 | 4,34E-07 | 2,41E-01 | 4,34E-07 | 1,07E+00 |
| Impa1 | 3,01E-01 | 4,77E-01 | 4,34E-07 | 3,01E-01 | 2,80E-01 | 2,64E-01 | 1,07E+00 |
| Chst11 | 3,05E-01 | 3,01E-01 | 3,01E-01 | 4,77E-01 | 2,31E-01 | 4,34E-07 | 1,07E+00 |
| S100a4 | 4,77E-01 | 3,01E-01 | 2,61E-01 | 4,34E-07 | 2,77E-01 | 2,81E-01 | 1,07E+00 |
| Glt8d1 | 6,02E-01 | 4,34E-07 | 4,34E-07 | 3,01E-01 | 2,46E-01 | 2,44E-01 | 1,06E+00 |
| Hars1 | 4,34E-07 | 6,99E-01 | 4,34E-07 | 4,34E-07 | 2,33E-01 | 2,39E-01 | 1,06E+00 |
| Slc35b2 | 3,01E-01 | 3,01E-01 | 3,01E-01 | 3,01E-01 | 4,34E-07 | 3,01E-01 | 1,04E+00 |
| Tmed3 | 3,01E-01 | 3,01E-01 | 3,01E-01 | 3,01E-01 | 4,34E-07 | 3,01E-01 | 1,04E+00 |
| Capza2 | 4,77E-01 | 4,77E-01 | 3,01E-01 | 4,34E-07 | 4,34E-07 | 4,34E-07 | 1,04E+00 |
| Nccrp1 | 3,01E-01 | 3,01E-01 | 3,01E-01 | 3,01E-01 | 4,34E-07 | 3,01E-01 | 1,04E+00 |
| Cap1 | 3,01E-01 | 3,01E-01 | 3,01E-01 | 4,77E-01 | 4,34E-07 | 4,34E-07 | 1,04E+00 |
| Gstp1 | 3,01E-01 | 3,01E-01 | 3,01E-01 | 3,01E-01 | 4,34E-07 | 3,01E-01 | 1,04E+00 |
| Lyz1 | 4,34E-07 | 4,77E-01 | 4,34E-07 | 3,01E-01 | 2,92E-01 | 3,01E-01 | 1,04E+00 |
| Tmed7 | 3,01E-01 | 3,01E-01 | 3,01E-01 | 4,34E-07 | 2,76E-01 | 3,01E-01 | 1,04E+00 |
| Tgm3 | 3,01E-01 | 4,34E-07 | 3,01E-01 | 3,01E-01 | 2,71E-01 | 3,01E-01 | 1,04E+00 |
| Cox6c | 3,01E-01 | 2,69E-01 | 4,77E-01 | 4,34E-07 | 4,34E-07 | 3,01E-01 | 1,04E+00 |
| Plod2 | 3,01E-01 | 4,77E-01 | 4,34E-07 | 3,01E-01 | 2,46E-01 | 4,34E-07 | 1,03E+00 |
| Ccdc47 | 3,01E-01 | 3,01E-01 | 4,34E-07 | 4,77E-01 | 2,46E-01 | 4,34E-07 | 1,03E+00 |
| Rpl28 | 4,77E-01 | 3,01E-01 | 4,34E-07 | 3,01E-01 | 2,43E-01 | 4,34E-07 | 1,03E+00 |
| St13 | 3,01E-01 | 4,34E-07 | 3,01E-01 | 3,01E-01 | 2,74E-01 | 2,71E-01 | 1,03E+00 |
| Cyb5b | 3,01E-01 | 4,77E-01 | 2,41E-01 | 3,01E-01 | 4,34E-07 | 4,34E-07 | 1,03E+00 |
| Dbi | 3,01E-01 | 3,01E-01 | 3,01E-01 | 3,01E-01 | 2,40E-01 | 4,34E-07 | 1,03E+00 |
| Rab23 | 4,34E-07 | 2,30E-01 | 4,34E-07 | 6,02E-01 | 1,81E-01 | 1,83E-01 | 1,03E+00 |
| Nomo1 | 3,01E-01 | 3,01E-01 | 3,01E-01 | 4,34E-07 | 2,77E-01 | 2,64E-01 | 1,03E+00 |
| Cox5a | 3,01E-01 | 3,01E-01 | 3,01E-01 | 3,01E-01 | 2,32E-01 | 4,34E-07 | 1,03E+00 |
| Prdx4 | 3,01E-01 | 3,01E-01 | 3,01E-01 | 3,01E-01 | 4,34E-07 | 2,30E-01 | 1,03E+00 |
| Rps6 | 3,01E-01 | 3,01E-01 | 3,01E-01 | 3,01E-01 | 2,29E-01 | 4,34E-07 | 1,03E+00 |
| Impa2 | 3,01E-01 | 3,01E-01 | 4,34E-07 | 3,01E-01 | 2,77E-01 | 2,46E-01 | 1,03E+00 |
| Slc6a9 | 4,77E-01 | 4,34E-07 | 3,01E-01 | 3,01E-01 | 2,15E-01 | 4,34E-07 | 1,03E+00 |
| Cnpy2 | 3,01E-01 | 3,01E-01 | 4,34E-07 | 3,01E-01 | 2,72E-01 | 2,38E-01 | 1,03E+00 |
| Glrx3 | 3,01E-01 | 3,01E-01 | 4,34E-07 | 3,01E-01 | 2,62E-01 | 2,45E-01 | 1,02E+00 |
| Camp | 3,01E-01 | 3,01E-01 | 4,34E-07 | 3,01E-01 | 2,66E-01 | 2,39E-01 | 1,02E+00 |
| Atp6v1a | 3,01E-01 | 3,01E-01 | 2,46E-01 | 4,34E-07 | 2,80E-01 | 2,78E-01 | 1,02E+00 |
| Aars1 | 3,01E-01 | 4,34E-07 | 3,01E-01 | 4,77E-01 | 1,86E-01 | 4,34E-07 | 1,02E+00 |
| Galnt1 | 3,01E-01 | 3,01E-01 | 4,34E-07 | 3,01E-01 | 2,60E-01 | 2,26E-01 | 1,02E+00 |
| Prnp | 3,01E-01 | 3,01E-01 | 2,51E-01 | 4,34E-07 | 2,75E-01 | 2,64E-01 | 1,02E+00 |
| Gale | 3,01E-01 | 3,01E-01 | 4,34E-07 | 3,01E-01 | 2,55E-01 | 2,31E-01 | 1,02E+00 |
| Sdc2 | 3,01E-01 | 2,61E-01 | 3,01E-01 | 4,34E-07 | 2,67E-01 | 2,58E-01 | 1,02E+00 |
| Scarb1 | 3,01E-01 | 3,01E-01 | 3,01E-01 | 2,42E-01 | 2,35E-01 | 4,34E-07 | 1,02E+00 |
| Igsf8 | 3,01E-01 | 3,01E-01 | 2,47E-01 | 4,34E-07 | 2,70E-01 | 2,61E-01 | 1,02E+00 |
| Cyp20a1 | 4,34E-07 | 3,01E-01 | 2,55E-01 | 3,01E-01 | 2,64E-01 | 2,59E-01 | 1,02E+00 |
| Cldnd1 | 3,01E-01 | 4,34E-07 | 2,56E-01 | 2,41E-01 | 2,77E-01 | 3,01E-01 | 1,02E+00 |
| Skp1 | 3,01E-01 | 4,34E-07 | 2,51E-01 | 3,01E-01 | 2,67E-01 | 2,55E-01 | 1,02E+00 |
| Fbn1 | 3,01E-01 | 3,01E-01 | 2,11E-01 | 2,54E-01 | 3,01E-01 | 4,34E-07 | 1,02E+00 |
| Lrp1b | 3,01E-01 | 4,34E-07 | 2,50E-01 | 3,01E-01 | 2,64E-01 | 2,54E-01 | 1,02E+00 |
| Asph | 3,01E-01 | 3,01E-01 | 2,25E-01 | 3,01E-01 | 2,34E-01 | 4,34E-07 | 1,02E+00 |
| Rps13 | 3,01E-01 | 4,34E-07 | 2,41E-01 | 3,01E-01 | 2,64E-01 | 2,56E-01 | 1,02E+00 |
| Vps4b | 4,34E-07 | 3,01E-01 | 2,44E-01 | 3,01E-01 | 2,58E-01 | 2,55E-01 | 1,02E+00 |
| Srprb | 3,01E-01 | 4,34E-07 | 4,77E-01 | 4,34E-07 | 2,21E-01 | 2,21E-01 | 1,01E+00 |
| Man1a2 | 3,01E-01 | 3,01E-01 | 2,07E-01 | 3,01E-01 | 2,32E-01 | 4,34E-07 | 1,01E+00 |
| Cacna1s | 2,38E-01 | 4,34E-07 | 3,01E-01 | 3,01E-01 | 2,54E-01 | 2,53E-01 | 1,01E+00 |
| Sdha | 2,56E-01 | 3,01E-01 | 4,34E-07 | 3,01E-01 | 2,49E-01 | 2,40E-01 | 1,01E+00 |
| Pbxip1 | 2,46E-01 | 3,01E-01 | 4,34E-07 | 3,01E-01 | 2,59E-01 | 2,35E-01 | 1,01E+00 |
| Hyou1 | 4,34E-07 | 2,21E-01 | 3,01E-01 | 3,01E-01 | 2,56E-01 | 2,53E-01 | 1,01E+00 |
| Ube2v2 | 3,01E-01 | 2,42E-01 | 4,34E-07 | 3,01E-01 | 2,53E-01 | 2,34E-01 | 1,01E+00 |
| Ftl1 | 3,01E-01 | 4,34E-07 | 2,46E-01 | 4,77E-01 | 4,34E-07 | 1,68E-01 | 1,01E+00 |
| Cdc37 | 4,34E-07 | 3,01E-01 | 4,34E-07 | 4,77E-01 | 1,98E-01 | 2,17E-01 | 1,01E+00 |
| Serpina1c | 3,01E-01 | 2,44E-01 | 4,34E-07 | 3,01E-01 | 2,50E-01 | 2,28E-01 | 1,01E+00 |
| Cand1 | 2,69E-01 | 3,01E-01 | 2,29E-01 | 3,01E-01 | 2,21E-01 | 4,34E-07 | 1,01E+00 |
| Sec62 | 4,77E-01 | 3,01E-01 | 4,34E-07 | 4,34E-07 | 2,00E-01 | 2,09E-01 | 1,01E+00 |
| Cpne2 | 3,01E-01 | 2,33E-01 | 4,34E-07 | 3,01E-01 | 2,51E-01 | 2,22E-01 | 1,01E+00 |
| Txn1 | 4,77E-01 | 4,34E-07 | 4,34E-07 | 3,01E-01 | 1,93E-01 | 2,01E-01 | 1,01E+00 |
| Galk1 | 4,77E-01 | 4,34E-07 | 4,34E-07 | 3,01E-01 | 1,82E-01 | 1,77E-01 | 1,00E+00 |
| Itga11 | 4,77E-01 | 3,01E-01 | 3,01E-01 | 4,34E-07 | 4,34E-07 | 4,34E-07 | 1,00E+00 |
| Kdelr1 | 4,34E-07 | 4,34E-07 | 3,01E-01 | 4,77E-01 | 4,34E-07 | 3,01E-01 | 1,00E+00 |
| Casq2 | 2,36E-01 | 4,34E-07 | 4,34E-07 | 6,02E-01 | 9,35E-02 | 4,34E-07 | 9,98E-01 |
| Sort1 | 4,34E-07 | 3,01E-01 | 3,01E-01 | 4,34E-07 | 2,55E-01 | 3,01E-01 | 9,91E-01 |
| C1qtnf3 | 4,77E-01 | 2,16E-01 | 4,34E-07 | 2,53E-01 | 1,30E-01 | 4,34E-07 | 9,90E-01 |
| Tada2b | 3,01E-01 | 3,01E-01 | 4,34E-07 | 4,34E-07 | 2,47E-01 | 3,01E-01 | 9,90E-01 |
| Cr1l | 4,34E-07 | 4,34E-07 | 3,01E-01 | 3,01E-01 | 2,38E-01 | 3,01E-01 | 9,88E-01 |
| Gpx8 | 4,34E-07 | 3,01E-01 | 4,34E-07 | 3,01E-01 | 2,38E-01 | 3,01E-01 | 9,88E-01 |
| Rps20 | 3,01E-01 | 3,01E-01 | 4,34E-07 | 3,01E-01 | 2,14E-01 | 4,34E-07 | 9,84E-01 |
| S100a6 | 4,34E-07 | 2,05E-01 | 3,01E-01 | 4,34E-07 | 3,01E-01 | 3,01E-01 | 9,82E-01 |
| Prdx5 | 3,01E-01 | 3,01E-01 | 4,34E-07 | 3,01E-01 | 1,88E-01 | 4,34E-07 | 9,80E-01 |
| Bcap31 | 3,01E-01 | 3,01E-01 | 4,34E-07 | 3,01E-01 | 1,87E-01 | 4,34E-07 | 9,80E-01 |
| Sparc | 3,01E-01 | 4,34E-07 | 3,01E-01 | 3,01E-01 | 1,84E-01 | 4,34E-07 | 9,79E-01 |
| Serpinb6a | 4,34E-07 | 3,01E-01 | 3,01E-01 | 3,01E-01 | 1,76E-01 | 4,34E-07 | 9,78E-01 |
| Manf | 3,01E-01 | 4,34E-07 | 3,01E-01 | 3,01E-01 | 1,75E-01 | 4,34E-07 | 9,78E-01 |
| Thy1 | 3,01E-01 | 4,34E-07 | 3,01E-01 | 3,01E-01 | 1,63E-01 | 4,34E-07 | 9,76E-01 |
| Chmp2a | 3,01E-01 | 3,01E-01 | 3,01E-01 | 4,34E-07 | 1,62E-01 | 4,34E-07 | 9,75E-01 |
| Rpl22 | 3,01E-01 | 4,34E-07 | 3,01E-01 | 3,01E-01 | 1,61E-01 | 4,34E-07 | 9,75E-01 |
| Flvcr2 | 3,01E-01 | 4,77E-01 | 4,34E-07 | 4,34E-07 | 1,40E-01 | 4,34E-07 | 9,72E-01 |
| Rpl10 | 4,77E-01 | 4,34E-07 | 4,34E-07 | 3,01E-01 | 1,38E-01 | 4,34E-07 | 9,72E-01 |
| Eif6 | 3,01E-01 | 4,77E-01 | 4,34E-07 | 4,34E-07 | 1,35E-01 | 4,34E-07 | 9,71E-01 |
| Ldhb | 4,77E-01 | 3,01E-01 | 4,34E-07 | 4,34E-07 | 1,23E-01 | 4,34E-07 | 9,70E-01 |
| Cisd1 | 4,77E-01 | 4,34E-07 | 4,34E-07 | 3,01E-01 | 1,19E-01 | 4,34E-07 | 9,69E-01 |
| Rpl34 | 4,77E-01 | 3,01E-01 | 4,34E-07 | 4,34E-07 | 1,11E-01 | 4,34E-07 | 9,68E-01 |
| Actr2 | 2,10E-01 | 4,34E-07 | 1,63E-01 | 4,77E-01 | 7,81E-02 | 4,34E-07 | 9,67E-01 |
| Agpat3 | 4,34E-07 | 4,77E-01 | 1,87E-01 | 4,34E-07 | 1,26E-01 | 1,46E-01 | 9,67E-01 |
| Esd | 4,77E-01 | 3,01E-01 | 4,34E-07 | 4,34E-07 | 1,04E-01 | 4,34E-07 | 9,67E-01 |
| Ube2n | 4,34E-07 | 3,01E-01 | 4,34E-07 | 4,77E-01 | 1,02E-01 | 4,34E-07 | 9,67E-01 |
| Rab6a | 1,77E-01 | 4,34E-07 | 1,44E-01 | 4,77E-01 | 4,34E-07 | 1,29E-01 | 9,66E-01 |
| Akr1b1 | 4,34E-07 | 4,77E-01 | 1,82E-01 | 4,34E-07 | 1,21E-01 | 1,37E-01 | 9,65E-01 |
| Rheb | 1,42E-01 | 4,34E-07 | 4,34E-07 | 4,77E-01 | 1,38E-01 | 1,45E-01 | 9,62E-01 |
| Rsu1 | 4,34E-07 | 4,34E-07 | 3,01E-01 | 3,01E-01 | 1,87E-01 | 2,03E-01 | 9,61E-01 |
| Tspan14 | 3,01E-01 | 3,01E-01 | 4,34E-07 | 1,99E-01 | 1,77E-01 | 4,34E-07 | 9,58E-01 |
| Tgoln1 | 4,77E-01 | 4,34E-07 | 1,43E-01 | 4,34E-07 | 1,30E-01 | 1,27E-01 | 9,58E-01 |
| Pfn1 | 3,01E-01 | 4,34E-07 | 4,34E-07 | 4,77E-01 | 4,34E-07 | 4,34E-07 | 9,54E-01 |
| Psmb7 | 4,77E-01 | 3,01E-01 | 4,34E-07 | 4,34E-07 | 4,34E-07 | 4,34E-07 | 9,54E-01 |
| Tmem119 | 3,01E-01 | 4,34E-07 | 4,77E-01 | 4,34E-07 | 4,34E-07 | 4,34E-07 | 9,54E-01 |
| Ddost | 3,01E-01 | 3,01E-01 | 3,01E-01 | 4,34E-07 | 4,34E-07 | 4,34E-07 | 9,54E-01 |
| Txnl1 | 4,34E-07 | 6,02E-01 | 4,34E-07 | 4,34E-07 | 4,34E-07 | 4,34E-07 | 9,54E-01 |
| Mapk3 | 3,01E-01 | 3,01E-01 | 3,01E-01 | 4,34E-07 | 4,34E-07 | 4,34E-07 | 9,54E-01 |
| Rtn3 | 4,34E-07 | 3,01E-01 | 3,01E-01 | 3,01E-01 | 4,34E-07 | 4,34E-07 | 9,54E-01 |
| Lgals3 | 3,01E-01 | 3,01E-01 | 4,34E-07 | 4,34E-07 | 4,34E-07 | 3,01E-01 | 9,54E-01 |
| Otub1 | 4,77E-01 | 4,34E-07 | 1,41E-01 | 4,34E-07 | 1,20E-01 | 1,06E-01 | 9,53E-01 |
| Tmem87a | 3,01E-01 | 4,34E-07 | 4,34E-07 | 3,01E-01 | 1,67E-01 | 1,60E-01 | 9,50E-01 |
| Chmp3 | 3,01E-01 | 3,01E-01 | 4,34E-07 | 4,34E-07 | 1,65E-01 | 1,52E-01 | 9,48E-01 |
| Mogs | 4,34E-07 | 3,01E-01 | 4,34E-07 | 3,01E-01 | 1,61E-01 | 1,53E-01 | 9,48E-01 |
| Pef1 | 3,01E-01 | 4,34E-07 | 4,34E-07 | 3,01E-01 | 1,63E-01 | 1,49E-01 | 9,48E-01 |
| Psmb3 | 3,01E-01 | 3,01E-01 | 4,34E-07 | 4,34E-07 | 1,57E-01 | 1,54E-01 | 9,47E-01 |
| Cnmd | 4,34E-07 | 3,01E-01 | 4,34E-07 | 3,01E-01 | 1,51E-01 | 1,56E-01 | 9,47E-01 |
| Mmp9 | 4,34E-07 | 3,01E-01 | 4,34E-07 | 3,01E-01 | 1,47E-01 | 1,50E-01 | 9,45E-01 |
| Slc4a1 | 3,01E-01 | 4,34E-07 | 4,34E-07 | 3,01E-01 | 1,43E-01 | 1,50E-01 | 9,45E-01 |
| Fam20b | 3,01E-01 | 3,01E-01 | 4,34E-07 | 4,34E-07 | 1,45E-01 | 1,44E-01 | 9,44E-01 |
| Cdc42 | 3,01E-01 | 4,34E-07 | 1,67E-01 | 3,01E-01 | 4,34E-07 | 1,15E-01 | 9,43E-01 |
| Bst2 | 3,01E-01 | 4,34E-07 | 4,34E-07 | 3,01E-01 | 1,38E-01 | 1,42E-01 | 9,42E-01 |
| Slc43a3 | 3,01E-01 | 3,01E-01 | 1,45E-01 | 4,34E-07 | 1,23E-01 | 4,34E-07 | 9,41E-01 |
| Ubtd1 | 3,01E-01 | 3,01E-01 | 4,34E-07 | 4,34E-07 | 1,34E-01 | 1,34E-01 | 9,41E-01 |
| Cavin1 | 4,34E-07 | 4,34E-07 | 4,34E-07 | 4,77E-01 | 1,03E-01 | 1,35E-01 | 9,36E-01 |
| Atp1b1 | 4,34E-07 | 4,34E-07 | 4,34E-07 | 4,77E-01 | 1,16E-01 | 1,22E-01 | 9,36E-01 |
| Prkca | 4,77E-01 | 4,34E-07 | 1,27E-01 | 4,34E-07 | 8,41E-02 | 4,34E-07 | 9,32E-01 |
| Tmem109 | 4,34E-07 | 4,34E-07 | 3,01E-01 | 4,34E-07 | 1,77E-01 | 3,01E-01 | 9,30E-01 |
| Tspan4 | 4,34E-07 | 4,34E-07 | 3,01E-01 | 4,34E-07 | 1,50E-01 | 3,01E-01 | 9,25E-01 |
| Fxyd1 | 4,34E-07 | 4,34E-07 | 3,01E-01 | 4,34E-07 | 1,50E-01 | 3,01E-01 | 9,25E-01 |
| Kcnma1 | 4,77E-01 | 4,34E-07 | 4,34E-07 | 4,34E-07 | 7,99E-02 | 7,35E-02 | 9,24E-01 |
| Cd276 | 4,34E-07 | 4,34E-07 | 3,01E-01 | 3,01E-01 | 1,08E-01 | 4,34E-07 | 9,18E-01 |
| Col1a2 | 3,01E-01 | 3,01E-01 | 4,34E-07 | 4,34E-07 | 1,03E-01 | 4,34E-07 | 9,17E-01 |
| Pof1b | 3,01E-01 | 3,01E-01 | 9,95E-02 | 4,34E-07 | 4,34E-07 | 4,34E-07 | 9,17E-01 |
| Hint1 | 4,34E-07 | 9,72E-02 | 3,01E-01 | 3,01E-01 | 4,34E-07 | 4,34E-07 | 9,16E-01 |
| Rps8 | 3,01E-01 | 4,34E-07 | 4,34E-07 | 3,01E-01 | 9,59E-02 | 4,34E-07 | 9,16E-01 |
| Psma1 | 3,01E-01 | 3,01E-01 | 4,34E-07 | 4,34E-07 | 9,26E-02 | 4,34E-07 | 9,16E-01 |
| Psmb6 | 3,01E-01 | 3,01E-01 | 4,34E-07 | 4,34E-07 | 9,12E-02 | 4,34E-07 | 9,16E-01 |
| Rps25 | 4,34E-07 | 3,01E-01 | 4,34E-07 | 3,01E-01 | 8,69E-02 | 4,34E-07 | 9,15E-01 |
| Paics | 3,01E-01 | 4,34E-07 | 4,34E-07 | 3,01E-01 | 8,67E-02 | 4,34E-07 | 9,15E-01 |
| Mdh2 | 3,01E-01 | 3,01E-01 | 4,34E-07 | 4,34E-07 | 8,61E-02 | 4,34E-07 | 9,15E-01 |
| Pcbp1 | 3,01E-01 | 3,01E-01 | 4,34E-07 | 4,34E-07 | 7,90E-02 | 4,34E-07 | 9,14E-01 |
| S100a13 | 3,01E-01 | 3,01E-01 | 4,34E-07 | 4,34E-07 | 7,45E-02 | 4,34E-07 | 9,13E-01 |
| Chst12 | 3,01E-01 | 3,01E-01 | 4,34E-07 | 4,34E-07 | 7,18E-02 | 4,34E-07 | 9,13E-01 |
| Hs2st1 | 3,01E-01 | 4,34E-07 | 4,34E-07 | 3,01E-01 | 7,04E-02 | 4,34E-07 | 9,13E-01 |
| Clptm1 | 3,01E-01 | 4,34E-07 | 3,01E-01 | 4,34E-07 | 4,34E-07 | 4,34E-07 | 9,03E-01 |
| Tmed9 | 4,34E-07 | 4,34E-07 | 3,01E-01 | 3,01E-01 | 4,34E-07 | 4,34E-07 | 9,03E-01 |
| Akt2 | 4,34E-07 | 4,77E-01 | 4,34E-07 | 4,34E-07 | 4,34E-07 | 4,34E-07 | 9,03E-01 |
| Mpzl1 | 3,01E-01 | 4,34E-07 | 3,01E-01 | 4,34E-07 | 4,34E-07 | 4,34E-07 | 9,03E-01 |
| Marcksl1 | 4,34E-07 | 4,34E-07 | 3,01E-01 | 4,34E-07 | 4,34E-07 | 3,01E-01 | 9,03E-01 |
| Eif5a | 4,34E-07 | 1,01E-01 | 3,01E-01 | 4,34E-07 | 8,88E-02 | 1,19E-01 | 8,92E-01 |
| Slc2a1 | 1,26E-01 | 1,05E-01 | 4,34E-07 | 3,01E-01 | 6,57E-02 | 4,34E-07 | 8,91E-01 |
| Gng5 | 4,34E-07 | 1,20E-01 | 3,01E-01 | 1,12E-01 | 6,24E-02 | 4,34E-07 | 8,90E-01 |
| Hapln3 | 7,39E-02 | 1,01E-01 | 4,34E-07 | 4,34E-07 | 1,14E-01 | 3,01E-01 | 8,89E-01 |
| Rpl17 | 1,28E-01 | 4,34E-07 | 4,34E-07 | 3,01E-01 | 8,08E-02 | 7,86E-02 | 8,89E-01 |
| Adss2 | 4,34E-07 | 4,34E-07 | 1,16E-01 | 3,01E-01 | 7,78E-02 | 8,25E-02 | 8,87E-01 |
| Amy2a2 | 4,34E-07 | 3,01E-01 | 9,95E-02 | 9,05E-02 | 4,34E-07 | 8,20E-02 | 8,86E-01 |
| Rhbdl2 | 4,34E-07 | 4,34E-07 | 1,40E-01 | 4,34E-07 | 1,16E-01 | 3,01E-01 | 8,86E-01 |
| Hmgcs1 | 4,34E-07 | 8,72E-02 | 3,01E-01 | 4,34E-07 | 7,61E-02 | 1,04E-01 | 8,86E-01 |
| Cox5b-ps | 9,50E-02 | 9,94E-02 | 3,01E-01 | 4,34E-07 | 7,12E-02 | 4,34E-07 | 8,85E-01 |
| Rtn2 | 1,05E-01 | 3,01E-01 | 9,48E-02 | 4,34E-07 | 5,51E-02 | 4,34E-07 | 8,84E-01 |
| Hapln4 | 7,80E-02 | 4,34E-07 | 3,01E-01 | 4,34E-07 | 6,95E-02 | 1,07E-01 | 8,84E-01 |
| Entpd1 | 3,01E-01 | 1,22E-01 | 4,34E-07 | 4,34E-07 | 6,37E-02 | 6,26E-02 | 8,83E-01 |
| Acp1 | 1,02E-01 | 4,34E-07 | 4,34E-07 | 3,01E-01 | 7,65E-02 | 6,62E-02 | 8,82E-01 |
| Gstm5 | 4,34E-07 | 4,34E-07 | 8,70E-02 | 3,01E-01 | 7,25E-02 | 8,18E-02 | 8,81E-01 |
| Itm2a | 3,01E-01 | 4,34E-07 | 1,02E-01 | 4,34E-07 | 6,15E-02 | 7,17E-02 | 8,81E-01 |
| Steap4 | 4,34E-07 | 4,34E-07 | 9,63E-02 | 3,01E-01 | 5,82E-02 | 7,32E-02 | 8,79E-01 |
| Rps27l | 3,01E-01 | 4,34E-07 | 9,96E-02 | 4,34E-07 | 6,06E-02 | 6,14E-02 | 8,78E-01 |
| Rac3 | 3,01E-01 | 4,34E-07 | 8,51E-02 | 4,34E-07 | 7,24E-02 | 6,31E-02 | 8,78E-01 |
| Spcs3 | 3,01E-01 | 4,34E-07 | 7,28E-02 | 4,34E-07 | 7,29E-02 | 7,24E-02 | 8,78E-01 |
| Chpf2 | 4,34E-07 | 3,01E-01 | 8,44E-02 | 4,34E-07 | 6,12E-02 | 7,22E-02 | 8,78E-01 |
| Golga7 | 3,01E-01 | 9,78E-02 | 4,34E-07 | 4,34E-07 | 6,07E-02 | 5,83E-02 | 8,78E-01 |
| Uqcrb | 9,61E-02 | 4,34E-07 | 4,34E-07 | 3,01E-01 | 5,41E-02 | 5,58E-02 | 8,76E-01 |
| B4galt1 | 4,34E-07 | 7,44E-02 | 4,34E-07 | 3,01E-01 | 5,99E-02 | 6,70E-02 | 8,75E-01 |
| Reep5 | 3,01E-01 | 4,34E-07 | 4,34E-07 | 1,32E-01 | 5,78E-02 | 4,34E-07 | 8,75E-01 |
| Myl10 | 4,34E-07 | 3,01E-01 | 8,26E-02 | 4,34E-07 | 5,61E-02 | 5,43E-02 | 8,74E-01 |
| Enpep | 4,34E-07 | 4,34E-07 | 7,67E-02 | 3,01E-01 | 5,12E-02 | 6,28E-02 | 8,74E-01 |
| Ppp3r1 | 4,34E-07 | 8,16E-02 | 4,34E-07 | 3,01E-01 | 5,31E-02 | 5,31E-02 | 8,73E-01 |
| Pcyox1 | 4,34E-07 | 4,34E-07 | 3,01E-01 | 4,34E-07 | 7,34E-02 | 1,07E-01 | 8,73E-01 |
| Mrc2 | 3,01E-01 | 4,34E-07 | 7,24E-02 | 4,34E-07 | 4,77E-02 | 5,18E-02 | 8,71E-01 |
| Cpt1b | 7,95E-02 | 4,34E-07 | 4,34E-07 | 3,01E-01 | 4,59E-02 | 4,47E-02 | 8,70E-01 |
| Adh5 | 1,14E-01 | 3,01E-01 | 4,34E-07 | 4,34E-07 | 4,69E-02 | 4,34E-07 | 8,70E-01 |
| Psen1 | 4,34E-07 | 4,34E-07 | 3,01E-01 | 4,34E-07 | 4,29E-02 | 1,04E-01 | 8,68E-01 |
| Fkbp1a | 4,34E-07 | 4,34E-07 | 7,85E-02 | 3,01E-01 | 6,72E-02 | 4,34E-07 | 8,67E-01 |
| Pth1r | 4,34E-07 | 3,01E-01 | 8,11E-02 | 4,34E-07 | 6,31E-02 | 4,34E-07 | 8,67E-01 |
| Rps27 | 3,01E-01 | 4,34E-07 | 8,49E-02 | 4,34E-07 | 3,76E-02 | 4,34E-07 | 8,64E-01 |
| Psma4 | 4,34E-07 | 3,01E-01 | 4,34E-07 | 4,34E-07 | 6,14E-02 | 6,05E-02 | 8,63E-01 |
| Vps37b | 4,34E-07 | 4,34E-07 | 4,34E-07 | 3,01E-01 | 5,39E-02 | 6,77E-02 | 8,63E-01 |
| Praf2 | 4,34E-07 | 4,34E-07 | 4,34E-07 | 4,34E-07 | 1,12E-01 | 3,01E-01 | 8,63E-01 |
| Tgm5 | 4,34E-07 | 3,01E-01 | 4,34E-07 | 4,34E-07 | 5,23E-02 | 6,21E-02 | 8,62E-01 |
| Mras | 4,34E-07 | 3,01E-01 | 4,34E-07 | 4,34E-07 | 5,12E-02 | 6,18E-02 | 8,62E-01 |
| Adam10 | 3,01E-01 | 4,34E-07 | 4,34E-07 | 4,34E-07 | 5,74E-02 | 5,54E-02 | 8,62E-01 |
| Endod1 | 3,01E-01 | 4,34E-07 | 4,34E-07 | 4,34E-07 | 6,07E-02 | 5,15E-02 | 8,62E-01 |
| Plpp1 | 4,34E-07 | 8,06E-02 | 4,34E-07 | 3,01E-01 | 2,29E-02 | 4,34E-07 | 8,61E-01 |
| St3gal6 | 4,34E-07 | 4,34E-07 | 4,34E-07 | 3,01E-01 | 4,41E-02 | 5,92E-02 | 8,61E-01 |
| Apoc1 | 4,34E-07 | 3,01E-01 | 4,34E-07 | 4,34E-07 | 4,67E-02 | 5,43E-02 | 8,60E-01 |
| Fkbp11 | 4,34E-07 | 4,34E-07 | 4,34E-07 | 4,34E-07 | 9,56E-02 | 3,01E-01 | 8,60E-01 |
| Rack1 | 4,34E-07 | 3,01E-01 | 4,34E-07 | 4,34E-07 | 4,23E-02 | 4,87E-02 | 8,59E-01 |
| Glud1 | 3,01E-01 | 4,34E-07 | 4,34E-07 | 4,34E-07 | 3,93E-02 | 4,61E-02 | 8,58E-01 |
| Capg | 4,34E-07 | 4,34E-07 | 4,34E-07 | 3,01E-01 | 3,77E-02 | 4,15E-02 | 8,57E-01 |
| Bax | 4,34E-07 | 3,01E-01 | 4,34E-07 | 4,34E-07 | 4,31E-02 | 3,28E-02 | 8,56E-01 |
| Vamp3 | 4,34E-07 | 4,34E-07 | 4,34E-07 | 3,01E-01 | 4,66E-02 | 4,34E-07 | 8,52E-01 |
| Mtpn | 4,34E-07 | 3,01E-01 | 4,34E-07 | 4,34E-07 | 4,64E-02 | 4,34E-07 | 8,52E-01 |
| Scarb2 | 4,34E-07 | 4,34E-07 | 4,34E-07 | 3,01E-01 | 4,46E-02 | 4,34E-07 | 8,52E-01 |
| Tmed1 | 4,34E-07 | 4,34E-07 | 4,34E-07 | 3,01E-01 | 4,20E-02 | 4,34E-07 | 8,51E-01 |
| Atp5mk | 4,34E-07 | 4,34E-07 | 4,34E-07 | 3,01E-01 | 3,69E-02 | 4,34E-07 | 8,51E-01 |
| Arf6 | 4,34E-07 | 3,01E-01 | 4,34E-07 | 4,34E-07 | 3,67E-02 | 4,34E-07 | 8,51E-01 |
| Sh3bgrl | 3,01E-01 | 4,34E-07 | 4,34E-07 | 4,34E-07 | 3,50E-02 | 4,34E-07 | 8,50E-01 |
| Yif1a | 4,34E-07 | 3,01E-01 | 4,34E-07 | 4,34E-07 | 3,37E-02 | 4,34E-07 | 8,50E-01 |
| Rpl36 | 3,01E-01 | 4,34E-07 | 4,34E-07 | 4,34E-07 | 2,61E-02 | 4,34E-07 | 8,49E-01 |
| Fxyd2 | 4,34E-07 | 3,01E-01 | 4,34E-07 | 4,34E-07 | 4,34E-07 | 4,34E-07 | 8,45E-01 |
| Cd151 | 4,34E-07 | 4,34E-07 | 4,34E-07 | 3,01E-01 | 4,34E-07 | 4,34E-07 | 8,45E-01 |
| Myo1b | 4,34E-07 | 4,34E-07 | 3,01E-01 | 4,34E-07 | 4,34E-07 | 4,34E-07 | 8,45E-01 |
| Slc39a7 | 4,34E-07 | 4,34E-07 | 3,01E-01 | 4,34E-07 | 4,34E-07 | 4,34E-07 | 8,45E-01 |
| Rcn2 | 4,34E-07 | 4,34E-07 | 3,01E-01 | 4,34E-07 | 4,34E-07 | 4,34E-07 | 8,45E-01 |
| Lims1 | 4,34E-07 | 4,34E-07 | 4,34E-07 | 4,34E-07 | 4,34E-07 | 4,34E-07 | 7,78E-01 |
| Sh3bgrl3 | 4,34E-07 | 4,34E-07 | 4,34E-07 | 4,34E-07 | 4,34E-07 | 4,34E-07 | 7,78E-01 |
| Bzw1 | 4,34E-07 | 4,34E-07 | 4,34E-07 | 4,34E-07 | 4,34E-07 | 4,34E-07 | 7,78E-01 |
| Gars1 | 4,34E-07 | 4,34E-07 | 4,34E-07 | 4,34E-07 | 4,34E-07 | 4,34E-07 | 7,78E-01 |
| Phpt1 | 4,34E-07 | 4,34E-07 | 4,34E-07 | 4,34E-07 | 4,34E-07 | 4,34E-07 | 7,78E-01 |
| Cpa4 | 4,34E-07 | 4,34E-07 | 4,34E-07 | 4,34E-07 | 4,34E-07 | 4,34E-07 | 7,78E-01 |
| Dynll1 | 4,34E-07 | 4,34E-07 | 4,34E-07 | 4,34E-07 | 4,34E-07 | 4,34E-07 | 7,78E-01 |
| Aloxe3 | 4,34E-07 | 4,34E-07 | 4,34E-07 | 4,34E-07 | 4,34E-07 | 4,34E-07 | 7,78E-01 |
| Tram2 | 4,34E-07 | 4,34E-07 | 4,34E-07 | 4,34E-07 | 4,34E-07 | 4,34E-07 | 7,78E-01 |
| Ahnak2 | 4,34E-07 | 4,34E-07 | 4,34E-07 | 4,34E-07 | 4,34E-07 | 4,34E-07 | 7,78E-01 |
| Tram1 | 4,34E-07 | 4,34E-07 | 4,34E-07 | 4,34E-07 | 4,34E-07 | 4,34E-07 | 7,78E-01 |
| Kdelr3 | 4,34E-07 | 4,34E-07 | 4,34E-07 | 4,34E-07 | 4,34E-07 | 4,34E-07 | 7,78E-01 |
| Fmod | 4,34E-07 | 4,34E-07 | 4,34E-07 | 4,34E-07 | 4,34E-07 | 4,34E-07 | 7,78E-01 |
| Slc39a10 | 4,34E-07 | 4,34E-07 | 4,34E-07 | 4,34E-07 | 4,34E-07 | 4,34E-07 | 7,78E-01 |
| Arl6ip5 | 4,34E-07 | 4,34E-07 | 4,34E-07 | 4,34E-07 | 4,34E-07 | 4,34E-07 | 7,78E-01 |
| Ap2b1 | 4,34E-07 | 4,34E-07 | 4,34E-07 | 4,34E-07 | 4,34E-07 | 4,34E-07 | 7,78E-01 |
| Nudt5 | 4,34E-07 | 4,34E-07 | 4,34E-07 | 4,34E-07 | 4,34E-07 | 4,34E-07 | 7,78E-01 |
| Immt | 4,34E-07 | 4,34E-07 | 4,34E-07 | 4,34E-07 | 4,34E-07 | 4,34E-07 | 7,78E-01 |
| Ap2m1 | 4,34E-07 | 4,34E-07 | 4,34E-07 | 4,34E-07 | 4,34E-07 | 4,34E-07 | 7,78E-01 |
| Sypl2 | 4,34E-07 | 4,34E-07 | 4,34E-07 | 4,34E-07 | 4,34E-07 | 4,34E-07 | 7,78E-01 |
| Ap2a1 | 4,34E-07 | 4,34E-07 | 4,34E-07 | 4,34E-07 | 4,34E-07 | 4,34E-07 | 7,78E-01 |
| Tmem167 | 4,34E-07 | 4,34E-07 | 4,34E-07 | 4,34E-07 | 4,34E-07 | 4,34E-07 | 7,78E-01 |
| Abhd12 | 4,34E-07 | 4,34E-07 | 4,34E-07 | 4,34E-07 | 4,34E-07 | 4,34E-07 | 7,78E-01 |
| Rpl9 | 4,34E-07 | 4,34E-07 | 4,34E-07 | 4,34E-07 | 4,34E-07 | 4,34E-07 | 7,78E-01 |
| Gk | 4,34E-07 | 4,34E-07 | 4,34E-07 | 4,34E-07 | 4,34E-07 | 4,34E-07 | 7,78E-01 |
| Atp6v1e1 | 4,34E-07 | 4,34E-07 | 4,34E-07 | 4,34E-07 | 4,34E-07 | 4,34E-07 | 7,78E-01 |
| Uqcr10 | 4,34E-07 | 4,34E-07 | 4,34E-07 | 4,34E-07 | 4,34E-07 | 4,34E-07 | 7,78E-01 |
| Prxl2b | 4,34E-07 | 4,34E-07 | 4,34E-07 | 4,34E-07 | 4,34E-07 | 4,34E-07 | 7,78E-01 |
| Gng11 | 4,34E-07 | 4,34E-07 | 4,34E-07 | 4,34E-07 | 4,34E-07 | 4,34E-07 | 7,78E-01 |
| Yipf6 | 4,34E-07 | 4,34E-07 | 4,34E-07 | 4,34E-07 | 4,34E-07 | 4,34E-07 | 7,78E-01 |
| Aldh9a1 | 4,34E-07 | 4,34E-07 | 4,34E-07 | 4,34E-07 | 4,34E-07 | 4,34E-07 | 7,78E-01 |
| Npm1 | 4,34E-07 | 4,34E-07 | 4,34E-07 | 4,34E-07 | 4,34E-07 | 4,34E-07 | 7,78E-01 |
| Spcs1 | 4,34E-07 | 4,34E-07 | 4,34E-07 | 4,34E-07 | 4,34E-07 | 4,34E-07 | 7,78E-01 |
| Cd68 | 4,34E-07 | 4,34E-07 | 4,34E-07 | 4,34E-07 | 4,34E-07 | 4,34E-07 | 7,78E-01 |
| Itgb6 | 4,34E-07 | 4,34E-07 | 4,34E-07 | 4,34E-07 | 4,34E-07 | 4,34E-07 | 7,78E-01 |
| Brox | 4,34E-07 | 4,34E-07 | 4,34E-07 | 4,34E-07 | 4,34E-07 | 4,34E-07 | 7,78E-01 |
| S100a16 | 4,34E-07 | 4,34E-07 | 4,34E-07 | 4,34E-07 | 4,34E-07 | 4,34E-07 | 7,78E-01 |
| Cd1d1 | 4,34E-07 | 4,34E-07 | 4,34E-07 | 4,34E-07 | 4,34E-07 | 4,34E-07 | 7,78E-01 |
| Sec22b | 4,34E-07 | 4,34E-07 | 4,34E-07 | 4,34E-07 | 4,34E-07 | 4,34E-07 | 7,78E-01 |
| Slc30a7 | 4,34E-07 | 4,34E-07 | 4,34E-07 | 4,34E-07 | 4,34E-07 | 4,34E-07 | 7,78E-01 |
| H2-Aa | 4,34E-07 | 4,34E-07 | 4,34E-07 | 4,34E-07 | 4,34E-07 | 4,34E-07 | 7,78E-01 |
| Ptpra | 4,34E-07 | 4,34E-07 | 4,34E-07 | 4,34E-07 | 4,34E-07 | 4,34E-07 | 7,78E-01 |
| Fcgrt | 4,34E-07 | 4,34E-07 | 4,34E-07 | 4,34E-07 | 4,34E-07 | 4,34E-07 | 7,78E-01 |
| Rabac1 | 4,34E-07 | 4,34E-07 | 4,34E-07 | 4,34E-07 | 4,34E-07 | 4,34E-07 | 7,78E-01 |
| Arpc3 | 4,34E-07 | 4,34E-07 | 4,34E-07 | 4,34E-07 | 4,34E-07 | 4,34E-07 | 7,78E-01 |
| Dad1 | 4,34E-07 | 4,34E-07 | 4,34E-07 | 4,34E-07 | 4,34E-07 | 4,34E-07 | 7,78E-01 |
| Apoh | 4,34E-07 | 4,34E-07 | 4,34E-07 | 4,34E-07 | 4,34E-07 | 4,34E-07 | 7,78E-01 |
| Ckmt2 | 4,34E-07 | 4,34E-07 | 4,34E-07 | 4,34E-07 | 4,34E-07 | 4,34E-07 | 7,78E-01 |
| Ralb | 4,34E-07 | 4,34E-07 | 4,34E-07 | 4,34E-07 | 4,34E-07 | 4,34E-07 | 7,78E-01 |
| Emc7 | 4,34E-07 | 4,34E-07 | 4,34E-07 | 4,34E-07 | 4,34E-07 | 4,34E-07 | 7,78E-01 |
| Vasn | 4,34E-07 | 4,34E-07 | 4,34E-07 | 4,34E-07 | 4,34E-07 | 4,34E-07 | 7,78E-01 |
| Ak1 | 4,34E-07 | 4,34E-07 | 4,34E-07 | 4,34E-07 | 4,34E-07 | 4,34E-07 | 7,78E-01 |
| Serpinb13 | 4,34E-07 | 4,34E-07 | 4,34E-07 | 4,34E-07 | 4,34E-07 | 4,34E-07 | 7,78E-01 |
| Entpd2 | 4,34E-07 | 4,34E-07 | 4,34E-07 | 4,34E-07 | 4,34E-07 | 4,34E-07 | 7,78E-01 |
| Rexo2 | 4,34E-07 | 4,34E-07 | 4,34E-07 | 4,34E-07 | 4,34E-07 | 4,34E-07 | 7,78E-01 |
| Txndc12 | 4,34E-07 | 4,34E-07 | 4,34E-07 | 4,34E-07 | 4,34E-07 | 4,34E-07 | 7,78E-01 |
| Tmx3 | 4,34E-07 | 4,34E-07 | 4,34E-07 | 4,34E-07 | 4,34E-07 | 4,34E-07 | 7,78E-01 |
| Vps26a | 4,34E-07 | 4,34E-07 | 4,34E-07 | 4,34E-07 | 4,34E-07 | 4,34E-07 | 7,78E-01 |
| Hspb1 | 4,34E-07 | 4,34E-07 | 4,34E-07 | 4,34E-07 | 4,34E-07 | 4,34E-07 | 7,78E-01 |
| Sppl2a | 4,34E-07 | 4,34E-07 | 4,34E-07 | 4,34E-07 | 4,34E-07 | 4,34E-07 | 7,78E-01 |
| Pvalb | 4,34E-07 | 4,34E-07 | 4,34E-07 | 4,34E-07 | 4,34E-07 | 4,34E-07 | 7,78E-01 |
| Smoc2 | 4,34E-07 | 4,34E-07 | 4,34E-07 | 4,34E-07 | 4,34E-07 | 4,34E-07 | 7,78E-01 |
| Trim11 | 4,34E-07 | 4,34E-07 | 4,34E-07 | 4,34E-07 | 4,34E-07 | 4,34E-07 | 7,78E-01 |
| Syngr2 | 4,34E-07 | 4,34E-07 | 4,34E-07 | 4,34E-07 | 4,34E-07 | 4,34E-07 | 7,78E-01 |
| Arpc4 | 4,34E-07 | 4,34E-07 | 4,34E-07 | 4,34E-07 | 4,34E-07 | 4,34E-07 | 7,78E-01 |
| Krt12 | 4,34E-07 | 4,34E-07 | 4,34E-07 | 4,34E-07 | 4,34E-07 | 4,34E-07 | 7,78E-01 |
| Uqcrh | 4,34E-07 | 4,34E-07 | 4,34E-07 | 4,34E-07 | 4,34E-07 | 4,34E-07 | 7,78E-01 |
| Vti1b | 4,34E-07 | 4,34E-07 | 4,34E-07 | 4,34E-07 | 4,34E-07 | 4,34E-07 | 7,78E-01 |
| Ube2d2a | 4,34E-07 | 4,34E-07 | 4,34E-07 | 4,34E-07 | 4,34E-07 | 4,34E-07 | 7,78E-01 |
| Mgp | 4,34E-07 | 4,34E-07 | 4,34E-07 | 4,34E-07 | 4,34E-07 | 4,34E-07 | 7,78E-01 |
| Ndufb4 | 4,34E-07 | 4,34E-07 | 4,34E-07 | 4,34E-07 | 4,34E-07 | 4,34E-07 | 7,78E-01 |
| Ebp | 4,34E-07 | 4,34E-07 | 4,34E-07 | 4,34E-07 | 4,34E-07 | 4,34E-07 | 7,78E-01 |
| Tspan18 | 4,34E-07 | 4,34E-07 | 4,34E-07 | 4,34E-07 | 4,34E-07 | 4,34E-07 | 7,78E-01 |
| Tgfbi | 4,34E-07 | 4,34E-07 | 4,34E-07 | 4,34E-07 | 4,34E-07 | 4,34E-07 | 7,78E-01 |
| Phf3 | 4,34E-07 | 4,34E-07 | 4,34E-07 | 4,34E-07 | 4,34E-07 | 4,34E-07 | 7,78E-01 |
| Naca | 4,34E-07 | 4,34E-07 | 4,34E-07 | 4,34E-07 | 4,34E-07 | 4,34E-07 | 7,78E-01 |
| Fabp5 | 4,34E-07 | 4,34E-07 | 4,34E-07 | 4,34E-07 | 4,34E-07 | 4,34E-07 | 7,78E-01 |
| Try10 | 4,34E-07 | 4,34E-07 | 4,34E-07 | 4,34E-07 | 4,34E-07 | 4,34E-07 | 7,78E-01 |
| Serpinc1 | 4,34E-07 | 4,34E-07 | 4,34E-07 | 4,34E-07 | 4,34E-07 | 4,34E-07 | 7,78E-01 |
| Blvrb | 4,34E-07 | 4,34E-07 | 4,34E-07 | 4,34E-07 | 4,34E-07 | 4,34E-07 | 7,78E-01 |
| Mgat1 | 4,34E-07 | 4,34E-07 | 4,34E-07 | 4,34E-07 | 4,34E-07 | 4,34E-07 | 7,78E-01 |
| Pon2 | 4,34E-07 | 4,34E-07 | 4,34E-07 | 4,34E-07 | 4,34E-07 | 4,34E-07 | 7,78E-01 |
| Xylt1 | 4,34E-07 | 4,34E-07 | 4,34E-07 | 4,34E-07 | 4,34E-07 | 4,34E-07 | 7,78E-01 |
| Ssr3 | 4,34E-07 | 4,34E-07 | 4,34E-07 | 4,34E-07 | 4,34E-07 | 4,34E-07 | 7,78E-01 |
| Sspn | 4,34E-07 | 4,34E-07 | 4,34E-07 | 4,34E-07 | 4,34E-07 | 4,34E-07 | 7,78E-01 |
| Cthrc1 | 4,34E-07 | 4,34E-07 | 4,34E-07 | 4,34E-07 | 4,34E-07 | 4,34E-07 | 7,78E-01 |
| Atp1b2 | 4,34E-07 | 4,34E-07 | 4,34E-07 | 4,34E-07 | 4,34E-07 | 4,34E-07 | 7,78E-01 |
| Tmem33 | 4,34E-07 | 4,34E-07 | 4,34E-07 | 4,34E-07 | 4,34E-07 | 4,34E-07 | 7,78E-01 |
| Arhgap1 | 4,34E-07 | 4,34E-07 | 4,34E-07 | 4,34E-07 | 4,34E-07 | 4,34E-07 | 7,78E-01 |
| Rpl22l1 | 4,34E-07 | 4,34E-07 | 4,34E-07 | 4,34E-07 | 4,34E-07 | 4,34E-07 | 7,78E-01 |
| Cct3 | 4,34E-07 | 4,34E-07 | 4,34E-07 | 4,34E-07 | 4,34E-07 | 4,34E-07 | 7,78E-01 |
| Ggct | 4,34E-07 | 4,34E-07 | 4,34E-07 | 4,34E-07 | 4,34E-07 | 4,34E-07 | 7,78E-01 |
| Lrpap1 | 4,34E-07 | 4,34E-07 | 4,34E-07 | 4,34E-07 | 4,34E-07 | 4,34E-07 | 7,78E-01 |
| Plscr1 | 4,34E-07 | 4,34E-07 | 4,34E-07 | 4,34E-07 | 4,34E-07 | 4,34E-07 | 7,78E-01 |
| Marveld1 | 4,34E-07 | 4,34E-07 | 4,34E-07 | 4,34E-07 | 4,34E-07 | 4,34E-07 | 7,78E-01 |
| Vamp8 | 4,34E-07 | 4,34E-07 | 4,34E-07 | 4,34E-07 | 4,34E-07 | 4,34E-07 | 7,78E-01 |
| Eif4h | 4,34E-07 | 4,34E-07 | 4,34E-07 | 4,34E-07 | 4,34E-07 | 4,34E-07 | 7,78E-01 |
| Pgm2 | 4,34E-07 | 4,34E-07 | 4,34E-07 | 4,34E-07 | 4,34E-07 | 4,34E-07 | 7,78E-01 |
| Kpnb1 | 4,34E-07 | 4,34E-07 | 4,34E-07 | 4,34E-07 | 4,34E-07 | 4,34E-07 | 7,78E-01 |
| Gng2 | 4,34E-07 | 4,34E-07 | 4,34E-07 | 4,34E-07 | 4,34E-07 | 4,34E-07 | 7,78E-01 |
| Ipo5 | 4,34E-07 | 4,34E-07 | 4,34E-07 | 4,34E-07 | 4,34E-07 | 4,34E-07 | 7,78E-01 |
| Gabarap | 4,34E-07 | 4,34E-07 | 4,34E-07 | 4,34E-07 | 4,34E-07 | 4,34E-07 | 7,78E-01 |
| Ces1c | 4,34E-07 | 4,34E-07 | 4,34E-07 | 4,34E-07 | 4,34E-07 | 4,34E-07 | 7,78E-01 |
| Myadm | 4,34E-07 | 4,34E-07 | 4,34E-07 | 4,34E-07 | 4,34E-07 | 4,34E-07 | 7,78E-01 |
| Fitm2 | 4,34E-07 | 4,34E-07 | 4,34E-07 | 4,34E-07 | 4,34E-07 | 4,34E-07 | 7,78E-01 |
| Cdsn | 4,34E-07 | 4,34E-07 | 4,34E-07 | 4,34E-07 | 4,34E-07 | 4,34E-07 | 7,78E-01 |
| Adpgk | 4,34E-07 | 4,34E-07 | 4,34E-07 | 4,34E-07 | 4,34E-07 | 4,34E-07 | 7,78E-01 |
| Smim5 | 4,34E-07 | 4,34E-07 | 4,34E-07 | 4,34E-07 | 4,34E-07 | 4,34E-07 | 7,78E-01 |
| Surf4 | 4,34E-07 | 4,34E-07 | 4,34E-07 | 4,34E-07 | 4,34E-07 | 4,34E-07 | 7,78E-01 |
| Atic | 4,34E-07 | 4,34E-07 | 4,34E-07 | 4,34E-07 | 4,34E-07 | 4,34E-07 | 7,78E-01 |
| mt-Co2 | 4,34E-07 | 4,34E-07 | 4,34E-07 | 4,34E-07 | 4,34E-07 | 4,34E-07 | 7,78E-01 |
| Slc35f6 | 4,34E-07 | 4,34E-07 | 4,34E-07 | 4,34E-07 | 4,34E-07 | 4,34E-07 | 7,78E-01 |
| Emp3 | 4,34E-07 | 4,34E-07 | 4,34E-07 | 4,34E-07 | 4,34E-07 | 4,34E-07 | 7,78E-01 |
| Yipf4 | 4,34E-07 | 4,34E-07 | 4,34E-07 | 4,34E-07 | 4,34E-07 | 4,34E-07 | 7,78E-01 |
| Tmem38a | 4,34E-07 | 4,34E-07 | 4,34E-07 | 4,34E-07 | 4,34E-07 | 4,34E-07 | 7,78E-01 |
| Vps28 | 4,34E-07 | 4,34E-07 | 4,34E-07 | 4,34E-07 | 4,34E-07 | 4,34E-07 | 7,78E-01 |
| Aph1a | 4,34E-07 | 4,34E-07 | 4,34E-07 | 4,34E-07 | 4,34E-07 | 4,34E-07 | 7,78E-01 |
| Eif1 | 4,34E-07 | 4,34E-07 | 4,34E-07 | 4,34E-07 | 4,34E-07 | 4,34E-07 | 7,78E-01 |
| Fbln7 | 4,34E-07 | 4,34E-07 | 4,34E-07 | 4,34E-07 | 4,34E-07 | 4,34E-07 | 7,78E-01 |
| Ighm | 4,34E-07 | 4,34E-07 | 4,34E-07 | 4,34E-07 | 4,34E-07 | 4,34E-07 | 7,78E-01 |
| Fkbp2 | 4,34E-07 | 4,34E-07 | 4,34E-07 | 4,34E-07 | 4,34E-07 | 4,34E-07 | 7,78E-01 |
| Rps7 | 4,34E-07 | 4,34E-07 | 4,34E-07 | 4,34E-07 | 4,34E-07 | 4,34E-07 | 7,78E-01 |
| Cpd | 4,34E-07 | 4,34E-07 | 4,34E-07 | 4,34E-07 | 4,34E-07 | 4,34E-07 | 7,78E-01 |
| Col9a3 | 4,34E-07 | 4,34E-07 | 4,34E-07 | 4,34E-07 | 4,34E-07 | 4,34E-07 | 7,78E-01 |

**Table S3. Full list of p-values generated in the statistical analysis of MV mineralization.**

| **Tukey's multiple comparisons test** | | **Adjusted P Value** |
| --- | --- | --- |
| MVs (10e9) + SMS2-IN-2 | MVs (10e9) | <0,0001 |
| MVs (10e9) + SMS2-IN-2 | MVs (10e9) + Levamisole | <0,0001 |
| MVs (10e9) + SMS2-IN-2 | MVs (10e7) + SMS2-IN-2 | 0.9998 |
| MVs (10e9) + SMS2-IN-2 | MVs (10e7) | <0,0001 |
| MVs (10e9) + SMS2-IN-2 | MVs (10e7) + Levamisole | <0,0001 |
| MVs (10e9) + SMS2-IN-2 | Vehicle | <0,0001 |
| MVs (10e9) + SMS2-IN-2 | Vehicle + SMS2-IN-2 | <0,0001 |
| MVs (10e9) + SMS2-IN-2 | Vehicle + Levamisole | <0,0001 |
| MVs (10e9) + SMS2-IN-2 | SCL buffer | <0,0001 |
| MVs (10e9) | MVs (10e9) + Levamisole | <0,0001 |
| MVs (10e9) | MVs (10e7) + SMS2-IN-2 | <0,0001 |
| MVs (10e9) | MVs (10e7) | <0,0001 |
| MVs (10e9) | MVs (10e7) + Levamisole | <0,0001 |
| MVs (10e9) | Vehicle | <0,0001 |
| MVs (10e9) | Vehicle + SMS2-IN-2 | <0,0001 |
| MVs (10e9) | Vehicle + Levamisole | <0,0001 |
| MVs (10e9) | SCL buffer | <0,0001 |
| MVs (10e9) + Levamisole | MVs (10e7) + SMS2-IN-2 | <0,0001 |
| MVs (10e9) + Levamisole | MVs (10e7) | <0,0001 |
| MVs (10e9) + Levamisole | MVs (10e7) + Levamisole | >0,9999 |
| MVs (10e9) + Levamisole | Vehicle | >0,9999 |
| MVs (10e9) + Levamisole | Vehicle + SMS2-IN-2 | >0,9999 |
| MVs (10e9) + Levamisole | Vehicle + Levamisole | 0.9662 |
| MVs (10e9) + Levamisole | SCL buffer | 0.6852 |
| MVs (10e7) + SMS2-IN-2 | MVs only (10e7) | <0,0001 |
| MVs (10e7) + SMS2-IN-2 | MVs (10e7) + Levamisole | <0,0001 |
| MVs (10e7) + SMS2-IN-2 | Vehicle | <0,0001 |
| MVs (10e7) + SMS2-IN-2 | Vehicle + SMS2-IN-2 | <0,0001 |
| MVs (10e7) + SMS2-IN-2 | Vehicle + Levamisole | <0,0001 |
| MVs (10e7) + SMS2-IN-2 | SCL buffer | <0,0001 |
| MVs (10e7) | MVs (10e7) + Levamisole | <0,0001 |
| MVs (10e7) | Vehicle | <0,0001 |
| MVs (10e7) | Vehicle + SMS2-IN-2 | <0,0001 |
| MVs (10e7) | Vehicle + Levamisole | <0,0001 |
| MVs (10e7) | SCL buffer | <0,0001 |
| MVs (10e7) + Levamisole | Vehicle | >0,9999 |
| MVs (10e7) + Levamisole | Vehicle + SMS2-IN-2 | >0,9999 |
| MVs (10e7) + Levamisole | Vehicle + Levamisole | 0.8296 |
| MVs (10e7) + Levamisole | SCL buffer | 0.4325 |
| Vehicle | Vehicle + SMS2-IN-2 | >0,9999 |
| Vehicle | Vehicle + Levamisole | 0.9693 |
| Vehicle only | SCL buffer only | 0.6962 |
| Vehicle + SMS2-IN-2 | Vehicle + Levamisole | 0.9793 |
| Vehicle + SMS2-IN-2 | SCL buffer | 0.7373 |
| Vehicle + Levamisole | SCL buffer | 0.9993 |

Mineralization kinetics by turbidity (absorbance at 340 nm) at the 26 hr timepoint (from Fig. 6e) whereas quantified. Statistical analysis was performed by one-way ANOVA (p-value < 0.0001), with Tukey´s post-hoc test results presented here. Selected significant differences are presented for visualization purposes on Fig. 6f. Note, that the vehicle to MVs was PBS-HAT storage buffer, whereas the vehicle to inhibitors was DMSO. Abbreviations: MVs, matrix vesicles; SMS2-IN-2: sphingomyelin synthase 2 inhibitor.
